# Unveiling New Reactivities in Complex Mixtures: Synthesis of Tricyclic Pyridinium Derivatives

**DOI:** 10.1021/jacs.4c15196

**Published:** 2025-01-30

**Authors:** Johanan Kootstra, Jaya Mehara, Marieke J. Veenstra, Maëlle Le Cacheux, Luca E. Oddone, Aleksandr Y. Pereverzev, Janaa Roithová, Syuzanna R. Harutyunyan

**Affiliations:** † Stratingh Institute for Organic Chemistry, University of Groningen, 9747 AG Groningen ,The Netherlands; ‡ Institute for Molecules and Materials, Radboud University, 6525 AJ Nijmegen, The Netherlands

## Abstract

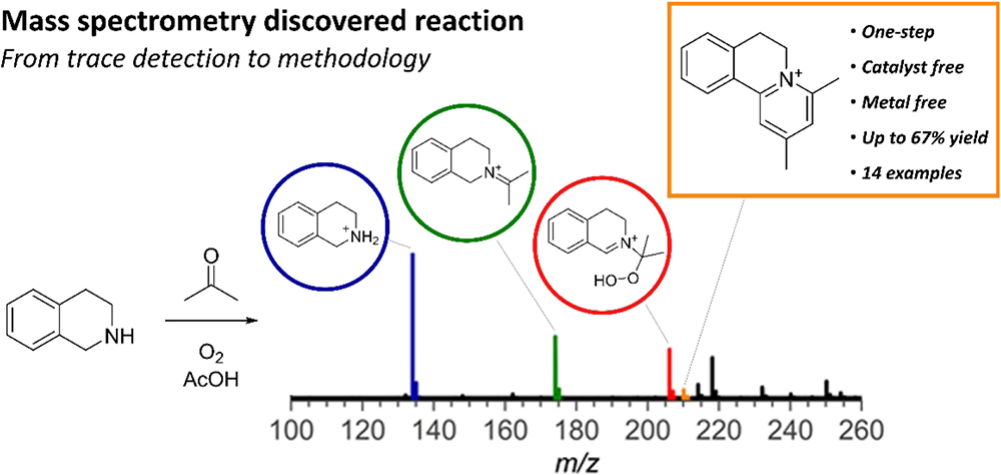

The discovery of new transformations drives the development of synthetic organic chemistry. While the main goal of synthetic chemists is to obtain the maximum yield of a desired product with minimal side product formation, meticulous characterization of the latter offers an opportunity for discovering new reaction pathways, alternative mechanisms, and new products. Herein, we present a case study on the discovery and development of a new chemical transformation using online mass spectrometry. This highly sensitive method enabled the discovery of a new reaction pathway in a catalyst-free cross-dehydrogenative coupling of 1,2,3,4-tetrahydroisoquinoline with acetone via peroxide intermediate, ultimately yielding a tricyclic pyridinium compound. Mass spectrometry was instrumental in detecting and identifying the structure of the pyridinium compound, initially formed as a trace byproduct, which allowed us to develop a general methodology for its exclusive formation.

## Introduction

The advancement of synthetic organic chemistry is fundamentally driven by the discovery of new transformations and reaction pathways. Traditional approaches in this endeavor have primarily focused on combining existing knowledge, applying high-throughput experimentation (HTE), or serendipitous findings. However, augmenting known chemistries builds on established reactivities and, therefore, often misses out on unveiling entirely new reactions, while the practicality of HTE, although offering a broader exploration scope, is hindered by challenges in managing and analyzing vast data sets.^1,2^ On the contrary, serendipity, unpredictable by its nature, has shown potential for acceleration within the HTE context, presenting an interesting blend of intentional search and fortuitous discovery.^3−5^

In contrast, an approach with deliberate examination of all reaction products, especially byproducts, is commonly overlooked. The conventional goal in synthetic chemistry has been to maximize the yield of the desired product, relegating byproducts to a position of lesser importance. However, these byproducts, particularly those formed in trace amounts, along with reactive intermediates, hold untapped potential for discovering new reaction pathways and mechanisms. Complex chemical reaction networks^6,7^ involving multiple components and transformations offer fertile ground for the discovery of unexpected reactions arising from previously unconsidered pathways. However, choosing the right analytical tools is essential for a methodical strategy to identify new reactions and reactivities within complex reaction mixtures. The ideal analytical instrument should not only detect trace elements in a reaction mixture but also offer capabilities for further characterization of these components. Online mass spectrometry meets these requirements, with its remarkable sensitivity for detecting minor components and facilitating direct analysis. Techniques like collision-induced dissociation (CID) and infrared photodissociation (IRPD) spectroscopy have also proven their utility in thoroughly characterizing trace byproducts and intermediates.^8^

In this work, we demonstrate how the detailed study of side reactions using online mass spectrometry can advance synthetic organic chemistry. By closely analyzing a specific reaction, we identified a trace byproduct resembling key scaffolds found in natural products, which guided the selective optimization of this compound’s production. Mechanistic studies further allowed us to propose a novel reaction pathway responsible for generating this product, underscoring the importance of exploring side reactions to uncover new chemistries.

## Results and Discussion

### Discovery of a New Reactivity

**Figure 1:**
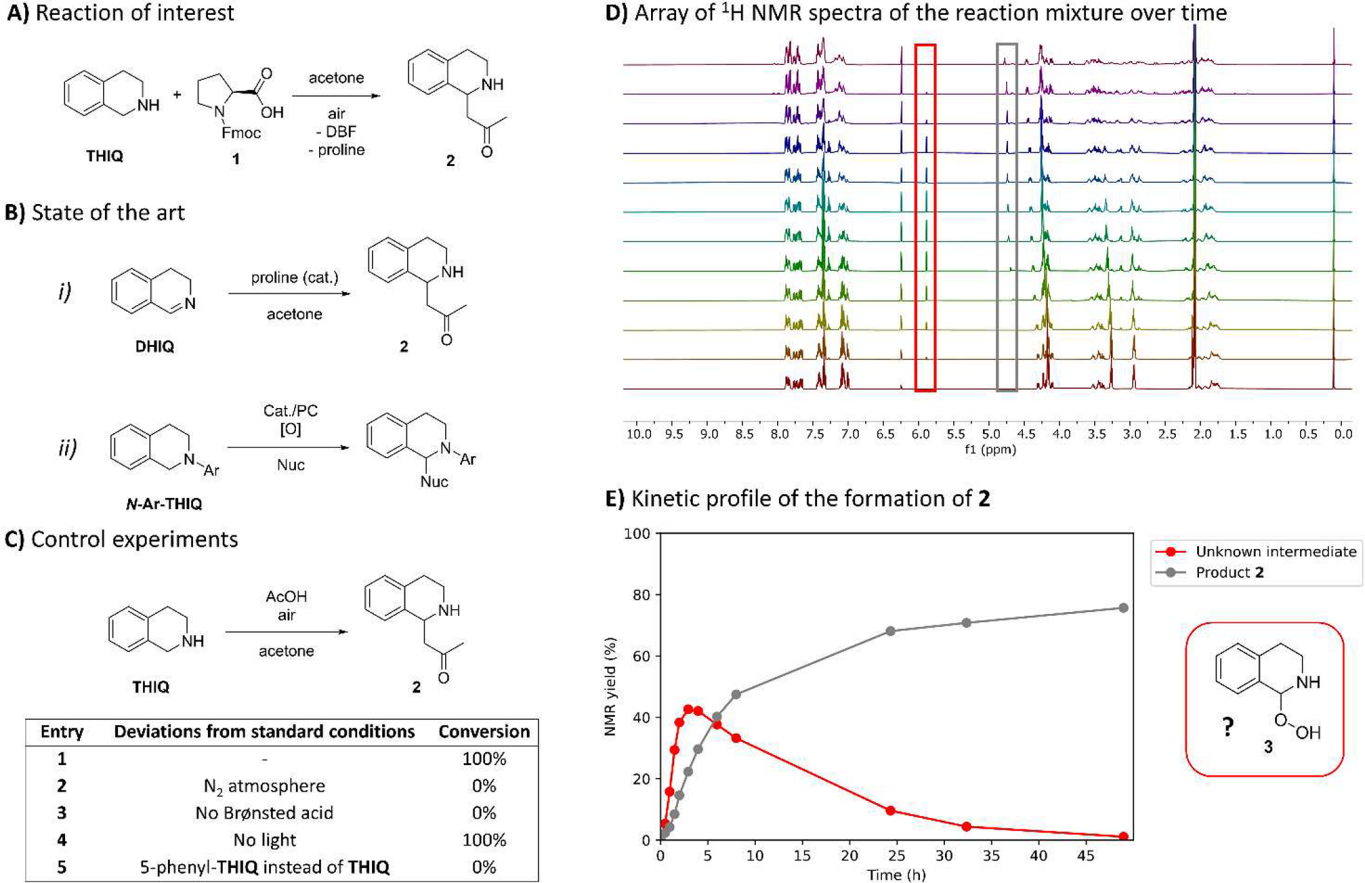
Discovery of unexpected reactivity between **THIQ** and acetone via an unknown intermediate. (A) Reaction of interest between **THIQ** and **1** to form **2**. Reaction conditions: **THIQ** (1 equiv) and **1** (1 equiv) in acetone, 48 h at room temperature. (B) State of the art: (i) proline-catalyzed additions of acetone to **DHIQ** and (ii) cross-dehydrogenative couplings starting from *N*-aryl-**THIQ**s. (C) Control experiments. (D) Array of ^1^H NMR spectra of samples taken from the reaction mixture depicted on A, with an unknown signal appearing and disappearing at 5.86 ppm (red box). (E) Integration results from the ^1^H NMR spectra of the unknown intermediate and product **2** over time plus a possible structure **3** similar to reported structures.

Through analyzing a cross-catalytic system reported by our team^7^ and control experiments, we identified unusual reactivity of 1,2,3,4-tetrahydroisoquinoline (**THIQ**), leading to several side products. The key reaction of interest involves the combination of Fmoc-protected proline **1** and **THIQ** in acetone, forming dibenzofulvene (**DBF**), proline, and unexpected product **2** (Figure 1A). The emergence of **2** is notable, as it typically forms through acetone addition to 3,4-dihydroisoquinoline (**DHIQ**) (Figure 1B, i)^9^ or cross-dehydrogenative coupling (CDC) between *N*-substituted **THIQ** and acetone, which usually requires a metal, a photocatalyst, or an organic oxidant (Figure 1B, ii).^10^ Control experiments showed that acetic acid (AcOH), acetone, and **THIQ** alone produce **2** (Figure 1C, entry 1), excluding the roles of **1** and **DBF** in this unexpected reactivity. Dioxygen (either from air or as O_2_) and a Bro̷nsted acid were crucial for the reaction (entries 2 and 3), while light was not required (entry 4). Interestingly, the secondary amine in **THIQ** was essential, as a substitution at the nitrogen site completely inhibited the reaction (entry 5).

Given the CDC-like transformation, we hypothesized the in situ formation of **DHIQ**, which would undergo nucleophilic addition from enolized acetone. To test this, we monitored the reaction using ^1^H NMR spectroscopy (Figure 1D). Contrary to expectations, we did not detect any peaks indicative of **DHIQ** (∼8.3 ppm).^7^ Instead, a peak at 4.40 ppm appeared, corresponding to the benzylic proton of product **2**, along with a singlet at 5.86 ppm, which diminished as the reaction progressed (Figure 1E). This singlet was attributed to an unknown intermediate. We speculated that the intermediate might be hydroperoxide **3**, similar to the peroxy intermediate proposed in the literature.^11^ This hypothesis was initially supported by increased peroxide levels, detected using peroxide test strips during the reaction. Nonetheless, subsequent control experiments did not confirm the presence of intermediate **3**.

### Identification of the Unknown Intermediate and Discovery of a New Reaction Product via Mass Spectrometry

**Figure 2:**
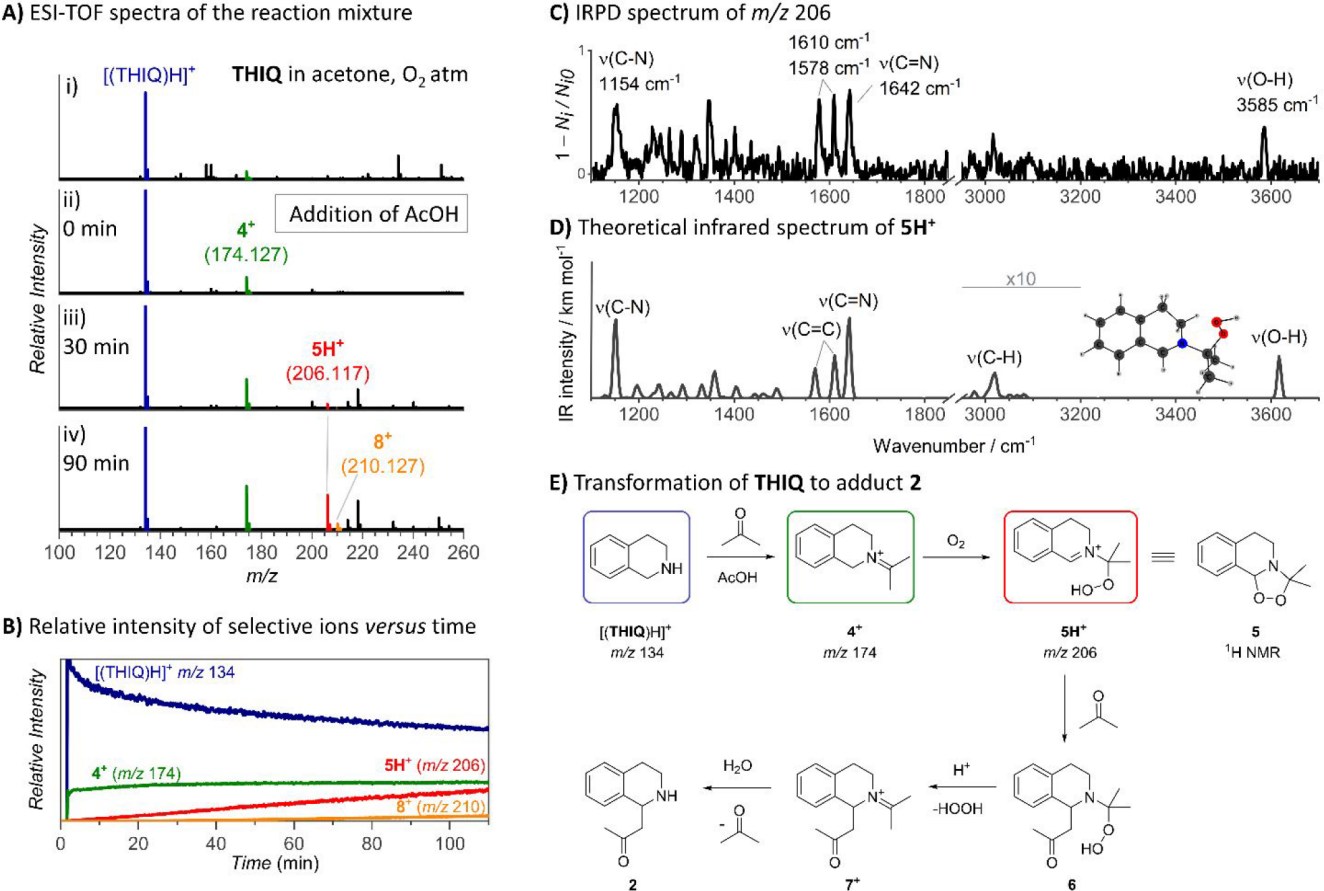
Detection and identification of a peroxide intermediate by mass spectrometry. (A) Snapshots of PSI-ESI-TOF spectra: (i) **THIQ** in acetone under an oxygen atmosphere, (ii) 1 equiv of AcOH added, (iii) after 30 min, and (iv) after 90 min. (B) Plot of the relative intensities of the specified ions versus time. (C) Helium tagging IRPD spectrum of the ions with *m*/*z* 206 measured at 3 K. (D) Theoretical IR spectrum of **5H**^**+**^ calculated using B3LYP/6-311+G**, GD3BJ, and the scaling factor of 0.98 for *v* < 2000 cm^–1^ and 0.96 for *v* > 2000 cm^–1^. (E) Proposed mechanistic pathway from **THIQ** to product **2** via peroxide **5**.

After challenges in characterizing the intermediate via ^1^H NMR spectroscopy, we turned to mass spectrometry for its sensitivity and ability to elucidate unknown structures, especially when coupled with ion spectroscopy.^6,12−16^ Pressurized sample infusion electrospray ionization mass spectrometry (PSI-ESI-MS) enables the sequential addition of components while continuous monitoring of reactions, providing insights into reaction pathways in solution. Using PSI-ESI-MS, we monitored **THIQ** in acetone under oxygen overpressure (Figure 2A, i). Adding AcOH to the solution of **THIQ** in acetone (ii) generated new ions (*m*/*z* 174), identified as iminium **4**^**+**^, the condensate of acetone and **THIQ** (Figure S3). These ions formed immediately upon preparing the reaction mixture (Figure 2B). Over time (iii), we observed an increasing abundance of other ions (*m*/*z* 206.117, red in Figure 2), corresponding to dioxygen addition to iminium ion **4**^**+**^. The structure of the oxygen-iminium adduct (*m*/*z* 206) was assigned using cryogenic ion spectroscopy. IR photodissociation (IRPD) spectra were measured at 3 K using the helium tagging technique.^17−19^

The IRPD spectrum aligned with the theoretical IR spectrum of **5H**^**+**^ (Figure 2C,D). However, the experimental ^1^H NMR spectrum did not match the predicted spectrum of **5H**^**+**^, leading us to propose that **5H**^**+**^ forms by protonating its neutral form in the solution. DFT calculations indicated that deprotonation of **5H**^**+**^ produces a zwitterion, which cyclizes to form neutral **5**. The theoretical ^1^H NMR spectrum of neutral **5** matched well with the experimental spectrum of the unknown intermediate. The structure of peroxide **5** supports its role as a reaction intermediate, as its protonated form can react with acetone to form **6**. Subsequently, elimination of hydrogen peroxide and hydrolysis of the resultant adduct (**7**^**+**^) yields product **2** (Figure 2E).

**Figure 3:**
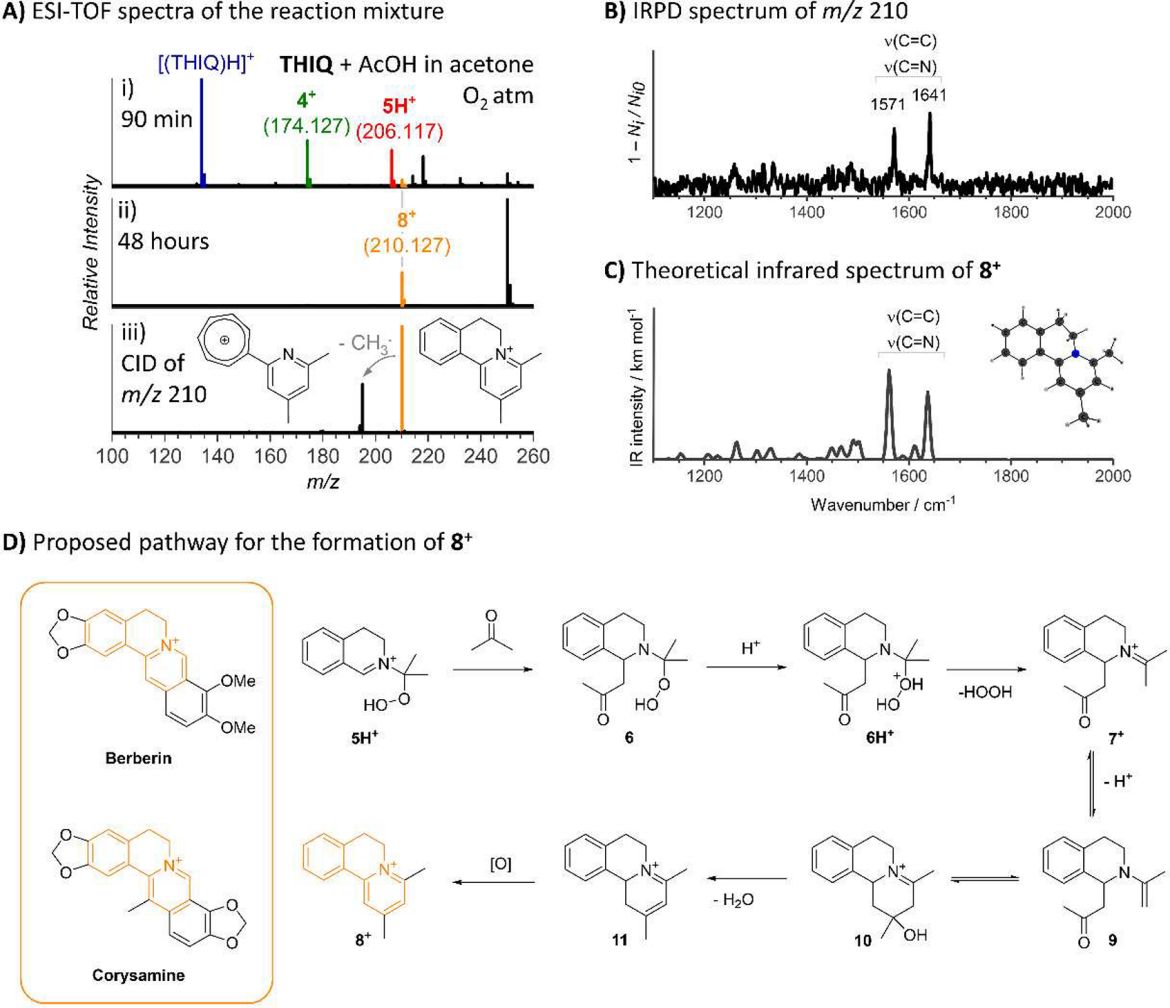
Detection and identification of a new reaction product. (A) Snapshots of PSI-ESI-TOF spectra showing growth of new ions at *m*/*z* 210 in the reaction mixture of **THIQ** (1 equiv) and AcOH (1 equiv) in acetone under an oxygen atmosphere: (i) after 90 min, (ii) after 48 h, and (iii) collision-induced dissociation of *m*/*z* 210, showing the methyl radical loss at the collision energy 30 eV. (B) Helium tagging IRPD spectrum of the ions with *m*/*z* 210 measured at 3 K. (C) Theoretical IR spectrum for **8**^**+**^ calculated using B3LYP/6-311+G**, GD3BJ, and a scaling factor of 0.98. (D) Proposed mechanistic pathway from **5H**^**+**^ to product **8**^**+**^ and natural products containing the structural motif of **8**^**+**^.

Identifying peroxide **5** is significant, as it provides experimental evidence for acyclic analogs that have been proposed in literature many times.^11,22,23^ While the oxidative coupling of *N*-aryl-**THIQ**s using *tert*-butylhydroperoxide (TBHP) produces a similar intermediate,^20,21^ to our knowledge, no analogous product resulting from dioxygen oxidation has been detected before. In addition to hydroperoxide **5H**^**+**^, ions with *m*/*z* 210 were observed, increasing in abundance after 48 h (Figure 3A, i and ii). Collison-induced dissociation (CID) of these ions revealed an exclusive fragmentation by a methyl radical loss at high collision energies (iii). Experiments with acetone-*d6* and phenylacetone showed that the methyl group did not originate from the acetone molecule (Figures S7–10). The IRPD spectrum of the ions with *m*/*z* 210 revealed two major bands at 1641 and 1571 cm^–1^. Screening possible structures by DFT calculations (Figure S11), we identified the ions as pyridinium **8**^**+**^ through the agreement between the observed and the theoretical IR spectrum.

Notably, pyridinium **8**^**+**^ went undetected by ^1^H NMR spectroscopy, underscoring the superior sensitivity of mass spectrometry in discovering new reaction products. The structure of **8**^**+**^ features a scaffold common in numerous natural products (Figure 3D),^24,25^ which explains its high stability and exclusive fragmentation by the CH_3_ radical loss from the **THIQ** backbone. The synthesis of pyridinium **8**^**+**^ under our conditions, albeit in trace amounts, is unprecedented and noteworthy as an entire pyridinium ring is added onto **THIQ**. We hypothesize that its formation starts from the addition of acetone to peroxide **5H**^**+**^ (Figure 3D). The loss of hydroperoxide produced the crucial intermediate, **7**^**+**^, which must tautomerize to enamine **9** to enable cyclization and formation of the new six-membered ring (**10**). This step is crucial, as hydrolysis of **7**^**+**^ would form **2**, and tautomerization of the ketone, instead of the iminium, would yield different products. From iminium **10**, the elimination of water (**11**) and subsequent oxidation complete the formation of the pyridinium product **8**^**+**^.

### Transforming a Trace Byproduct into the Primary Reaction Product

**Figure 4:**
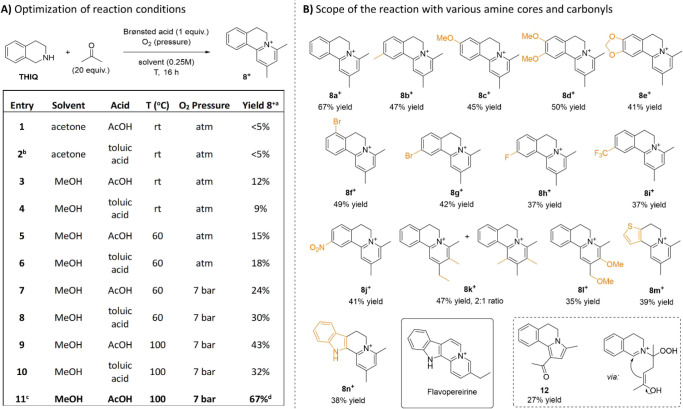
Optimization of reaction conditions toward the formation of **8^+^** and scope of the reaction with various amine cores and carbonyls. (A) Reaction conditions: **THIQ** (1 equiv), acetone (20 equiv), and the Bro̷nsted acid (1 equiv) were dissolved in solvent (0.25 M), and the atmosphere of O_2_ was applied. ^a^The crude mixture was analyzed by ^1^H NMR after 16 h. ^b^0.05 M concentration of the substrate; ^c^2 equiv of AcOH; ^d^Isolated yield. (B) Reaction conditions: The corresponding THIQ (1 equiv), ketone (20 equiv), and AcOH (2 equiv) were dissolved in MeOH (0.25 M), pressurized with 7 bar of O_2_ and heated to 100 °C. The reaction was monitored by GC-MS, until complete consumption of the substrate. Products were obtained as diacetate salts, of which the counterion was omitted for clarity. Formation of product **12** using the same protocol at room temperature.

Given the importance of these scaffolds, we embarked on turning the pyridinium byproduct **8**^**+**^ into the main product and develop a reliable method for its synthesis. We optimized the reaction conditions, starting with **THIQ** in acetone and one equivalent of AcOH. We found that the p*K*_a_ of Bro̷nsted acid was critical: strong acids (p*K*_a_ < 0) precipitated the substrate as the ammonium salt, halting the reaction, while weak acids (p*K*_a_ > 8) led to the slow formation of peroxide **5** without further reactions (Table S3). A weaker acid faces two challenges: (i) it must protonate and open the ring of **5** before acetone adds, and (ii) acetone must be tautomerized before the nucleophilic attack can occur. Acetic and (Figure 4A, entry 1) and toluic acids (entry 2) gave good **THIQ** conversion, although the low solubility of toluic acid in acetone limited its reactivity. These acids displayed increased selectivity for forming **8**^**+**^, although still observed in negligible quantities.

We anticipated that a polar protic solvent could enhance this reaction by stabilizing charged intermediates and facilitating tautomerization.

Switching from acetone to methanol (MeOH, entries 3 and 4) significantly improved selectivity. Raising the temperature to 60 °C further increased selectivity for both acids (entries 5 and 6). It was found that increasing the oxygen pressure was beneficial for both conversion and product formation (entries 7 and 8). Upon further increase of the temperature to 100 °C, full conversion was reached for both acids (entries 9 and 10), where AcOH yielded the better selectivity (43% yield). Optimization with two equivalents of AcOH (entry 11) allowed isolation of pyridinium product **8**^**+**^ with a 67% yield, with diacetate as the counterion.

Next, we explored the reaction scope, starting with substitutions on the **THIQ** core (Figure 4B). Electron-donating groups at the 6- and 7-positions of **THIQ** were well tolerated, leading to products (**8b–e**^**+**^) in 41–50% yields, including pharmaceutically relevant 6,7-dimethoxy (**8d**^**+**^) and 6,7-methylenedioxy (**8e**^**+**^) derivatives. Notably, electron-deficient **THIQ**s, such as those with fluoro- and bromo-substituents at the 5- and 7-positions (**8f–h**^**+**^), and stronger electron-withdrawing groups like nitro (**8i**^**+**^) and trifluoromethyl (**8j**^**+**^), also yielded products. The ketone scope was narrower, with certain substituents disrupting the balance required for product formation (Figure S27 for attempted substrates). Methyl ethyl ketone was tolerated, producing a 2:1 ratio of inseparable regioisomers with a 47% yield (**8k**^**+**^), while methoxyacetone yielded a single isomer (**8l**^**+**^) in 30%. Finally, **THIQ**-like scaffolds yielded thiophene- and indole-based products (**8m**^**+**^ and **8n**^**+**^), with **8n**^**+**^ being a direct analog of the natural product *Flavopereirine*.^26^

Given that two ketone molecules are needed to form the pyridinium core, we hypothesized that diones might also undergo this transformation. Under the optimized conditions at room temperature, 2,5-hexanedione was consumed, yielding pyrrole derivative **12**, although in a low yield. This product, featuring a pharmaceutically relevant scaffold,^27^ forms via intramolecular ring closure following the peroxide intermediate formation. Its structure was confirmed by X-ray crystallography.

### Mechanistic Investigations

**Figure 5:**
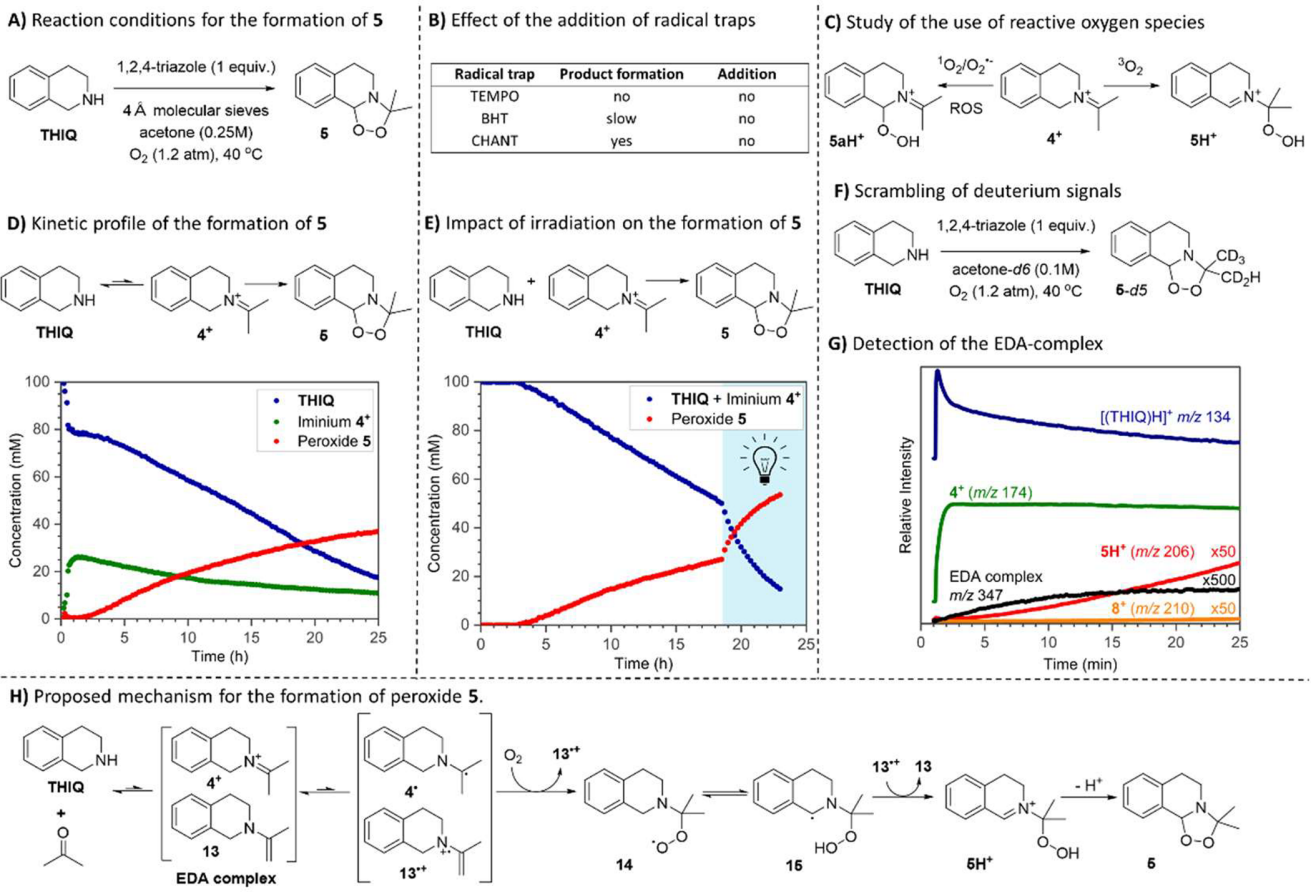
Mechanistic investigations for the formation of peroxide **5**. (A) Optimized conditions for the synthesis of peroxide **5.** (B) Effect of the addition of radical trapping agents (1 equiv) to the reaction mixture. (C) Proposed pathways for the distinct reactions between iminium **4**^+^ and several reactive oxygen species. Reactions with ^1^O_2_ and O_2_^–^ lead to peroxide **5aH**^**+**^, whereas reactions with ^3^O_2_ lead to peroxide **5.** (D) Kinetic profile of the reaction using optimized conditions excluding molecular sieves by ^1^H NMR in flow. (E) Kinetic profile of the reaction with irradiation of 455 nm blue light after 18.5 h, leading to an increase in reaction rate. (F) Scrambling of the deuterium of acetone-*d6* because of iminium–enamine interconversion in the reaction mixture. (G) Detection of the EDA complex in the reaction mixture by online mass spectrometry: Plot of relative ion intensities (corrected by total ion chromatogram) of specified *m*/*z* vs time. (H) Proposed mechanism for the formation of peroxide **5**.

Peroxide **5** is a key intermediate in the formation of pyridinium products. To better understand its formation, we optimized the reaction conditions to exclusively produce and isolate **5**. Using a weak acid (p*K*_a_ > 8), the peroxide was obtained as the sole product. 1,2,4-Triazole (p*K*_a_ = 9.4) proved optimal, achieving full **THIQ** conversion after 48 h, with ^1^H NMR yields of **5** ranging from 50 to 70% (Figure 5A). The reaction was accelerated by adding 4 Å molecular sieves and raising the temperature to 40 °C, as the sieves promoted the irreversible formation of iminium ion **4**^**+**^ by removing water. These conditions enabled the isolation of pure **5** (see Supporting Information). Based on previous reports of aerobic oxidations, we suspected a radical mechanism of the formation of **5**.^28^ To test this, we added radical inhibitors TEMPO and BHT. TEMPO completely halted the **THIQ** conversion, where BHT significantly slowed down the reaction (13% ^1^H NMR yield). No radical trapping products were detected, even with the CHANT radical trap,^29^ leaving the radical nature unknown (Figure 5B). Using mass spectrometry, we examined reactions with ^1^O_2_ and KO_2_, excluding triplet oxygen (Figure S12). In both cases, an intermediate with *m*/*z* 206, the same mass as peroxide **5**, was produced. However, fragmentation of these ions revealed a mixture of **5H**^**+**^ and a new isomer, **5aH**^**+**^ (Figure 5C). Deprotonation of both leads to peroxide **5**, suggesting similar follow-up reactivity. Consistently, pyridinium **8**^**+**^ (*m*/*z* 210) was detected in all experiments.

These results indicate that singlet oxygen or superoxide anions can initiate radical formation of **5**, but the radical species differ from those involved in the presence of triplet oxygen in solution. The question remains: how is radical chemistry initiated without an external radical source? **THIQ**s are known to form electron donor–acceptor (EDA) complexes that can lead to product formation under irradiation.^30−33^ To investigate whether an EDA complex is involved in the formation of peroxide **5**, we studied the reaction kinetics using ^1^H NMR in flow, allowing for both qualitative analysis and quantification (Figure S14 for setup).

Under the optimized conditions for the peroxide **5** formation, iminium **4**^**+**^ was detected within 30 min after adding the Bro̷nsted acid. After 2 h, peroxide **5** began to form, reaching a 40% yield and 70% conversion of **THIQ** after 24 h (Figure 5D). To confirm the role of an EDA complex, we irradiated the reaction mixture with blue LEDs, which significantly increased the reaction rate (Figure 5E).

Notably, in this case, a ground-state EDA complex enabled 70% conversion, whereas in the report by König and Gschwind,^30^ a similar pathway only occurred in trace amounts. This difference is likely due to the thermodynamic stability of peroxide **5**, which shifts the EDA complex equilibrium toward its formation. The complex likely forms before the rate-determining step, combining two **THIQ** derivatives: iminium **4**^**+**^ and enamine **13** (Scheme S2). Iminium **4**^**+**^ is the likely acceptor, being the most electron-poor variant of **THIQ**, while enamine **13**, although not observed by ^1^H NMR, is the likely donor, as reported for similar systems.^34^

To investigate the involvement of the enamine **13**, we performed the reaction in acetone-*d6* (Figure 5F). We observed the scrambling of, on average, one of the six deuterium atoms in the deuterated peroxide, confirming the tautomerization of the iminium **4**^**+**^ to enamine **13** during the reaction. If the EDA complex forms between iminium **4**^**+**^ and enamine **13**, these species could enable electron transfer in both the excited and ground states, forming radicals **4·** and **13·**^**+**^**.** To further support the formation of the EDA complex, we reanalyzed the reaction mixture using mass spectrometry. Upon adding AcOH to **THIQ** in acetone under oxygen, new ions with *m*/*z* 347 were detected (Figure 5G), consistent with the proposed EDA complex of iminium **4**^**+**^ and enamine **13**. Previous work suggests that dioxygen can drive the EDA complex formation by making the electron transfer between the donor and the acceptor irreversible through the reaction with the formed radicals.^30^ In our study, dioxygen may react with radical **4·** to form an oxygen-based radical that abstracts a hydrogen atom intramolecularly from the benzylic position (Figure 5H). Supporting this, we measured a primary kinetic isotope effect (KIE) *k*_H_/*k*_D_ in a direct competition experiment with **THIQ** versus **THIQ**-*d2* (Figure S20). These insights led us to propose the mechanism for the formation of peroxide **5** (Figure 5H). EDA complex between iminium **4**^**+**^ and enamine **13** leads to a single-electron transfer (SET), forming radicals **4·** and **13·**^**+**^. Radical **4·** is subsequently captured by dioxygen, forming the peroxy radical **14**. Intramolecular hydrogen atom abstraction generates benzylic radical **15**, which, through electron transfer with **13·**^**+**^, forms **5H**^**+**^. Deprotonation of **5H**^**+**^ produces **5**. The interaction between the iminium and enamine, along with the involvement of dioxygen and benzylic radical, allows ground-state electron transfer from the EDA complex to be sufficiently thermodynamically favorable to reach full conversion.

## Conclusions

In summary, we demonstrated that online mass spectrometry is an ideal tool for identifying and characterizing new reactivities in complex mixtures. Using this tool, we identified the unstable peroxide **5** derived from **THIQ**, which undergoes subsequent reaction steps to form the novel product, pyridinium **8**^**+**^. We optimized the formation of this species and proposed a mechanistic pathway for peroxide **5** formation, supported by extensive experimental evidence. Crucially, a ground-state EDA complex, supported by mechanistic studies, plays a key role in the process.

## Supporting Information Available

Experimental procedures, characterization data, mass spectrometry and NMR spectra, and crystallographic data in CIF format for **12** (PDF)
