# Supplementary material for: Unveiling New Reactivities in Complex Mixtures: Synthesis of Tricyclic Pyridinium Derivatives
Source: J Am Chem Soc. 2025 Jan 30;147(6):5132–9. doi: 10.1021/jacs.4c15196 (PMC11826991; doi:10.1021/jacs.4c15196)
Supplement: Supplementary file 1 — ja4c15196_si_001.pdf [file ja4c15196_si_001.pdf]

Supplementary Information

Unveiling New Reactivities in Complex Mixtures: Synthesis of Tricyclic Pyridinium Derivatives.

Johanan Kootstra[1‡], Jaya Mehara[2‡], Marieke J. Veenstra[1], Maëlle Le Cacheux[1], Luca E. Oddone[1], Aleksandr Y. Pereverzev[2], Jana[a] Roithová[2*], Syuzanna R. Harutyunyan[1*]

[1]Stratingh Institute for Organic Chemistry, University of Groningen; Groningen, 9747 AG, the Netherlands.

[2]Institute for Molecules and Materials, Radboud University; Nijmegen, 6525 AJ, the Netherlands.

‡These authors contributed equally to this work

*Corresponding authors. Email: jana.roithova@ru.nl, s.harutyunyan@rug.nl.

KEYWORDS pyridinium, online mass spectrometry, organic peroxide, aerobic, catalyst free, cross dehydrogenative coupling .

Contents

General experimental information 1
General chemical information 2
Measurements for the discovery of peroxide **5** and pyridinium **8**$^+$ 3
  Detection of peroxide **5** using ^{1}H NMR 3
  Detection and identification of iminium **4**$^+$ peroxide **5** and **8**$^+$ using mass spectrometry 4
Mechanistic study of the formation of peroxide **5** 10
  Procedure for the synthesis of peroxide **5** 10
  Studying the involvement of the reactive oxygen species using mass spectrometry 10
  Measuring kinetic profiles of the reaction using NMR in flow 11
  General procedure for measuring the kinetics of the formation of peroxide **5** 12
  Irradiation of the reaction mixture with blue light 14
  Determination of the kinetic isotope effect of the benzylic position of **THIQ** 15
  Determination of the reaction order in **THIQ** 16
  Detection of enamine **13** using scrambling experiments 17
  Detection of the EDA complex by mass spectrometry 19
Screening of conditions for the formation of pyridinium **8**$^+$ 20
  Screening of Brønsted acids: 21
  Screening of solvents: 21
Synthesis of substrates 22
  Synthesis of 6,7-dioxymethylene-1,2,3,4-tetrahydroisoquinoline (**THIQe**) 22
  Synthesis of **THIQ**-*d2* 22
  Synthesis of N-cyclohexyl-2-{[(2,2,6,6-tetramethylpiperidin-1-yl)oxy]methyl}acrylamide (CHANT) 23
Attempted ketones 24
Synthesis of pyridinium products **8**$^+$ 24
  General procedure for synthesis of pyridinium products **8**$^+$ 24
  Characterization 24
  Synthesis and characterization of pyrrole product **12** 32
NMR spectra 35
XYZ-coordinates 55
References 61

General experimental information

Mass spectrometric experiments were conducted using a Thermo Scientific LTQ XL(general conditions are in Table S1) and a Bruker tims-TOF, both equipped with electrospray ionization (ESI) sources.[1,2] Mass calibration with a tune mix solution was performed for the TOF analysis to ensure high-resolution *m/z* information. Instrumental parameters were optimized for soft ionization conditions. Aliquots of the reaction mixture were injected for offline analysis or online monitoring using pressurized sample infusion-electrospray ionization-mass spectrometry (PSI-ESI-MS).[3,4] Depending on the experimental requirements, either nitrogen or oxygen overpressure was used for PSI-ESI-MS.

The tims-TOF instrument provides ion-mobility separation of the ions. We have tested the ion mobility resolution of the detected ions. Only single isomers were detected for the iminium ions **4**$^+$ and the pyridinium products **8**$^+$. Importantly, we attempted to resolve the hydroperoxo isomers **5H**$^+$ and **5aH**$^+$. Unfortunately, the switching on the tims separation results in harsher conditions for the ion transfer. Fragile ions do not survive the tims separation, which is often the case for reactive species. This is what happened also with the hydroperoxo ions. They were fragmented in the tims section, and their survival abundance was too low to permit any analysis. This is the reason why we generally worked without the tims separation.

Tuning Parameter/Setting	Experimental range
Sheath gas flow rate	5-40 arbitrary units
Auxiliary gas flow rate	0-10 arbitrary units
Capillary temperature	150-220 ℃
Spray voltage	4-5 kV
Capillary voltage	5-30 V
Tube lens voltage	5-40 V
Activation time in CID experiments	30 ms
Activation parameter q in CID experiments	0.25

Table S1. *LTQ settings during the ESI-MS experiments*.

The IR spectra of the mass-selected complexes were measured on the ISORI instrument equipped with an ESI source using the helium tagging photodissociation method.[5] The ISORI (ion spectroscopy of reactive intermediates) is a homebuilt instrument with a quadrupole trap operated at 3-5 K, where trapped ions are cooled down by helium buffer gas. These cooled ions then attach a helium atom. Upon irradiation with an IR laser, the helium complexes undergo helium detachment, and the IR spectra are constructed as $(1 - N(\nu_i)/N_0)$, where $N(\nu_i)$ and N_0 are the numbers of helium complexes with and without laser irradiation. An OPO/OPA system from LaserVision was used for irradiation.

Geometrical structures were minimized at B3LYP/6–311+G**, GD3BJ using Gaussian 16 code.[6]

Unless otherwise noted, all reaction were carried out either in a 5 ml steel autoclave with Teflon insert (<1 mmol) or in a 25 mL Büchi Tinyclave with a glass reactor tube. Reaction conversions were monitored by GC MS on a Shimadzu Gas Chromatograph GC-2010 with a GCMS-QP2010

mass spectrometer Ultrawidth equipped with a HP-5MS or VF-17MS column and He carrier gas. Purification of the products was performed by extraction or column chromatography using Merck 60 Å 230-400 mesh silica gel or neutral active Al_2O_3 90. Components were visualized by UV irradiation or $KMnO_4$ staining of thin layer chromatography (TLC) plates.

NMR data was collected on a Varian MercuryPlus (^{1}H at 400 MHz; ^{19}F at 376 MHz; ^{13}C at 101 MHz) equipped with a 400 Autosw probe, an Agilent 400MR (^{1}H at 400 MHz; ^{19}F at 376 MHz; ^{13}C at 101 MHz) equipped with a OneNMR probe, a Varian Inova 500 (^{1}H at 500 MHz) equipped with a Varian 5 mm PFG SW probe and a Bruker NEO (^{1}H at 600 MHz; ^{13}C at 151 MHz) equipped with a SmartProbe BBFO or a Prodigy BBO. Chemical shifts are reported in parts per million (ppm) relative to residual solvent peak (chloroform-*d*, ^{1}H: 7.26 ppm; ^{13}C: 77.16 ppm; DMSO-*d6*, ^{1}H: 2.50 ppm, ^{13}C: 39.52 ppm; acetone-*d6*, ^{1}H: 2.05 ppm, ^{13}C: 29.84 ppm). Coupling constants are reported in Hertz (Hz). Multiplicity is reported with the usual abbreviations (s: singlet, d: doublet, dd: doublet of doublets, td: triplet of doublets, t: triplet, dt: doublet of triplets, q: quadruplet, p: quintet, m: multiplet).

Flow NMR studies were performed using the flow tube of a Bruker InsightMr, connected to 1/16” microtubing, driven by a LeadFluid BT100L peristaltic pump. As a reaction flask an ELVEFLOW 4 ports microfluidic reservoir GL45 bottle cap with 100 mL flask was used. ^{1}H NMR spectra were collected on a Varian MercuryPlus (^{1}H at 200 MHz) equipped with a 200 Autosw probe, with WET suppression of the solvent peak. Alternating ^{1}H and ^{2}H NMR spectra were recorded on a Varian MercuryPlus (^{1}H at 200 MHz; ^{2}H at 30.7 MHz) equipped with a Varian broadband probe tuned to the ^{2}H frequency and disabled lock, with PreSat suppression of the solvent peak. Irradiation of the reaction mixture was done using a THORLABS M455L4 LED, in combination with a THORLABS LEDD1B LED-driver.

Exact mass spectra were recorded on a Thermo Scientific LTQ Orbitrap XL with ESI ionization or a Thermo Scientific Orbitrap Exploris 480 with ESI ionization.

General chemical information

Unless otherwise indicated, reagents and substrates were purchased from commercial sources and used as received. Dry solvents were purchased or collected from a solvent purification system. Solvents not required to be dry were purchased as technical grade and used as received. Commercial **THIQ**s were purchased either in pure form or as hydrochloride salts (Table S1). The latter were extracted with DCM from NaOH (1M in H_2O) and used as such. **THIQe**[7], **THIQ**-*d2*[8] and CHANT[9] were prepared according to literature procedures and were characterized by ^{1}H NMR. **THIQ**-*d2* was also characterized by ^{13}C NMR to confirm deuterium incorporation. New compounds were characterized by ^{1}H NMR, ^{13}C NMR and HRMS.

Table S2. *Source of* ***THIQ*** *substrates*

THIQ	Name	CAS	Source
**a**	1,2,3,4-tetrahydroisoquinoline	91-21-4	Sigma Aldrich
**b**	7-methyl-1,2,3,4-tetrahydrosioquinoline	207451-81-8	BLDpharm
**c**	6-methoxy-1,2,3,4-tetrahydroisoquinoline hydrochloride salt	57196-62-0	BLDpharm
**d**	6,7-dimethoxy-1,2,3,4-tetrahydroisoquinoline	1745-07-9	BLDpharm
**f**	5-bromo-1,2,3,4-tetrahydroisoquinoline	81237-69-6	BLDpharm
**g**	7-bromo-1,2,3,4- tetrahydroisoquinoline	17680-55-6	Sigma Aldrich
**h**	7-fluoro-1,2,3,4-tetrahydroisoquinoline hydrochloride salt	799274-06-9	Sigma Aldrich
**i**	7-trifluoromethyl-1,2,3,4-tetrahydroisoquinoline hydrochloride salt	220247-87-0	BLDpharm
**j**	7-nitro-1,2,3,4-tetrahydroisoquinoline	42923-79-5	BLDpharm
**m**	4,5,6,7-tetrahydrothieno[3,2-*c*]pyridine	54903-50-3	TCI Chemicals
**n**	1,2,3,4-Tetrahydro-9*H*-pyrido[3,4-*b*]indole	16502-01-5	Sigma Aldrich

Measurements for the discovery of peroxide 5 and pyridinium 8^+

Detection of peroxide **5** using ^{1}H NMR

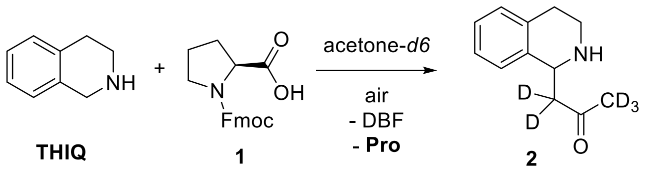

To a Schlenk tube equipped with a Teflon coated stirring bar, **THIQ** (178 mg, 1.33 mmol, 1.0 equiv.), Fmoc-proline (**1**, 449 mg, 1.33 mmol, 1.0 equiv.), HMDSO (internal standard, 13 mg, 0.08 mmol, 6 mol%) and acetone-*d6* (10.0 mL, 133mM) were added. The reaction mixture was placed under an oxygen atmosphere using a rubber balloon and stirred at room temperature. Samples were taken from the reaction mixture and a ^{1}H NMR spectrum was recorded directly after. The kinetic profile is plotted in Figure S1.

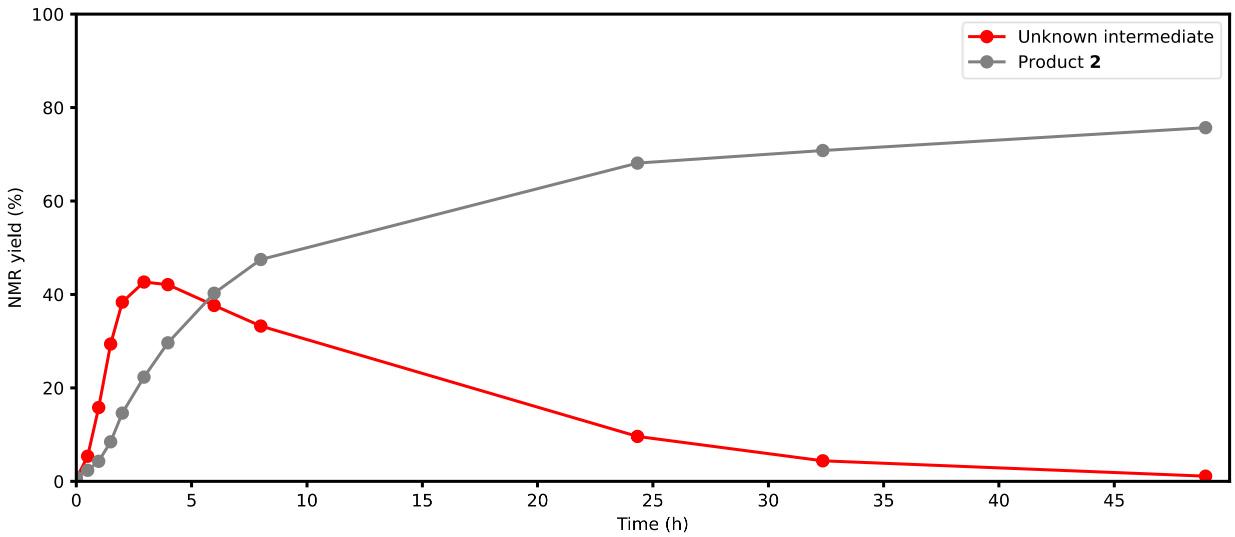

Figure S1. Kinetic profile of the reaction between **THIQ** and **1** to form adduct **2** and an unknown intermediate

Detection and identification of iminium **4**$^+$ peroxide **5** and **8**$^+$ using mass spectrometry

Using online mass spectrometry on LTQ XL equipped with ESI, we tracked the reaction changes over time and by adding different reagents. To the stirred solution of **THIQ** (500 µM) in acetone, 1 equivalent of AcOH was added, and then the oxygen atmosphere was introduced, and mass spectra were recorded for 3 hours. This solution was kept overnight under the oxygen atmosphere and analyzed after 12 hours (Figure S2).

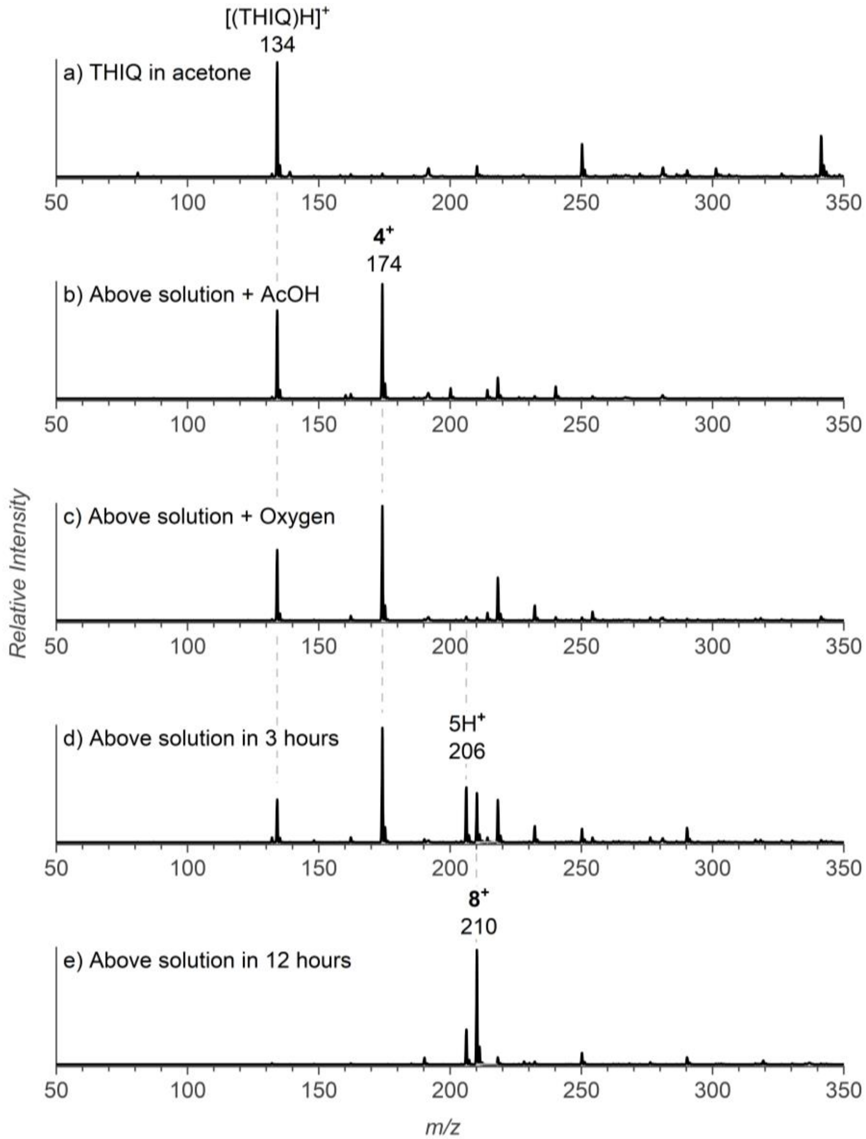

Figure S2. ESI-MS spectra of the reaction mixture: a) **THIQ** (500 uM) in acetone, b) 1 eq AcOH added, c) oxygen balloon added, d) after 3 hours, and e) after 12 hours. *m/z* 134 = [(**THIQ**)H]$^+$, *m/z* 174 = **4**$^+$, *m/z* 206 = **5H**$^+$, *m/z* 210 = **8**$^+$

The ions with *m/z* 174 were characterized as the condensate of acetone with **THIQ**, iminium **4**$^+$, using IRPD (Figure S3). The ions with *m/z* 206 were characterized as the addition of dioxygen to iminium **4**$^+$, peroxide **5H**$^+$, using CID (Figure S4) and IRPD (Figure S5).

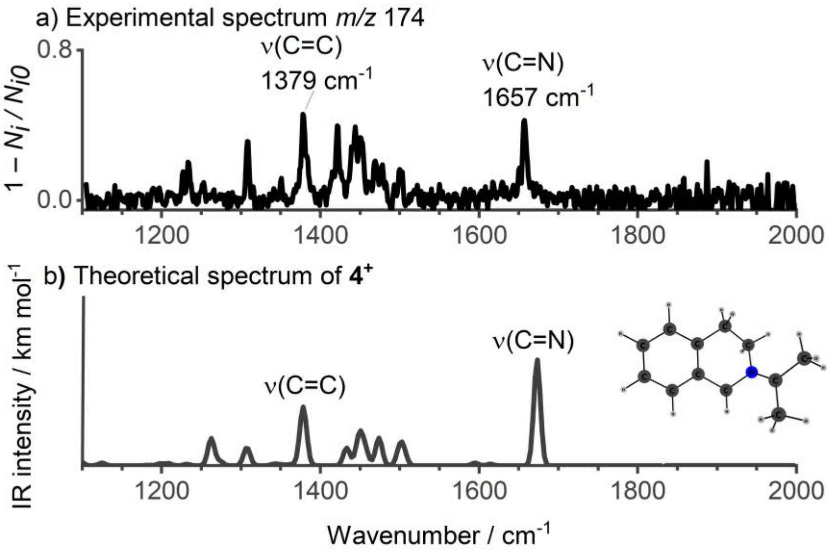

Figure S3. a) Helium tagging IRPD spectrum of the ions with *m/z* 174 measured at 3 K, and b) theoretical IR spectrum for **4**$^+$ calculated using B3LYP/6–311+G**, GD3BJ, and the scaling factor of 0.98.

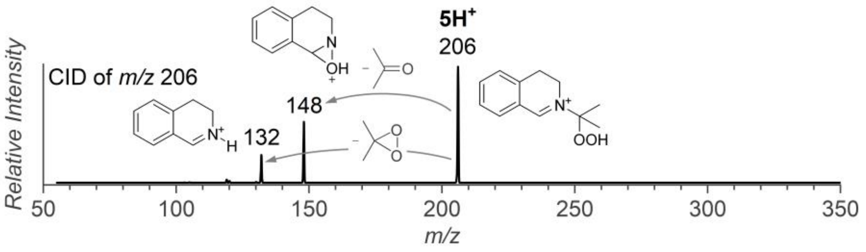

Figure S4. CID spectrum of **5H**$^+$ (*m/z* 206) at the normalized collision energy 12%.

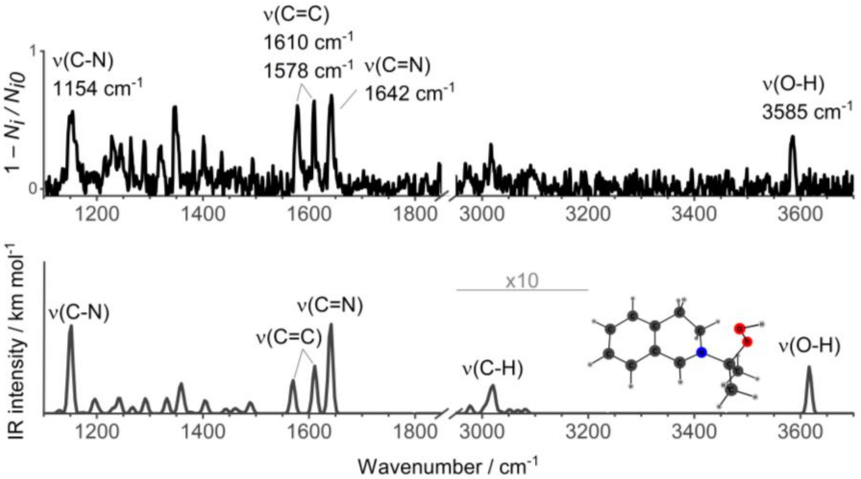

Figure S5. Helium tagging IRPD spectrum of **5H**$^+$ (*m/z* 206) measured at 3 K (black, top), and theoretical IR spectrum of **5H**$^+$ (grey, bottom) calculated using B3LYP/6–311+G**, GD3BJ, and the scaling factor of 0.98 for $v < 2000\ cm^{-1}$ and 0.96 for $v > 2000\ cm^{-1}$.

The unknown side product, which shows a characteristic loss of the methyl radical, exhibits different mass-to-charge (*m/z*) depending on the solvent/ketone used: *m/z* 210 in acetone (Figure S6), *m/z* 218 in acetone-*d6* (Figure S7 and S8), and *m/z* 362 in phenylacetone (Figure S9 and S10). These observations suggest that two ketone molecules are involved in the formation process, as evidenced by the incorporation of eight deuterium atoms in acetone-*d6* compared to regular acetone. The masses of the ions suggested that the reaction sequence leading to the unknown side products involved twice a ketone addition followed by the loss of a water molecule and once H_2 elimination (i.e., + 2 ketones – 2 H_2O – H_2).

Furthermore, the consistent fragmentation pattern showing the exclusive elimination of the CH_3 radical indicates that this radical is derived from the **THIQ** backbone rather than from the acetone.

This pattern aligns with a reaction mechanism involving the second ketone reacting with the corresponding iminium ion, followed by dehydration and oxidation/dehydrogenation. Based on this information, we calculated the theoretical IR spectra of possible isomers for possible structures of the ions with *m/z* 210, where structure **8**$^+$ matched the experimental spectrum (Figure S11).

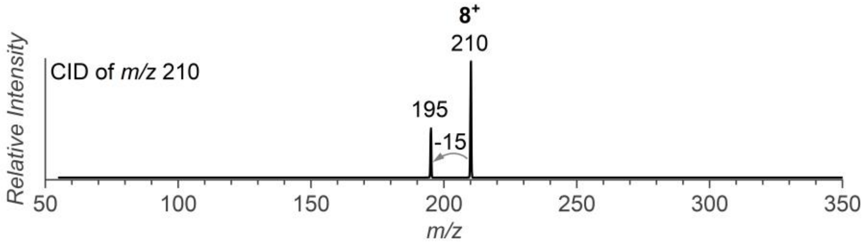

Figure S6. CID spectrum of **8**$^+$ (*m/z* 210) at the normalized collision energy 30%, the only fragmentation is the loss of the methyl radical.

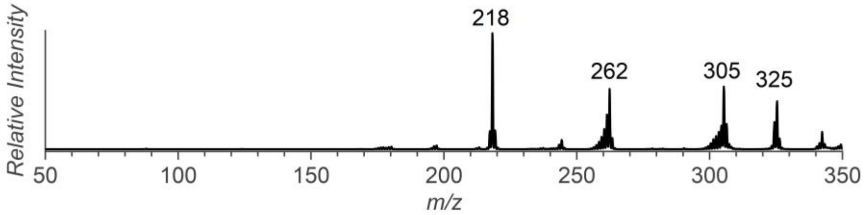

Figure S7. ESI-MS spectrum of the reaction mixture of THIQ (500 uM) in acetone-*d6*, 1 eq. AcOH, under the oxygen atmosphere, after 1 hour reaction time.

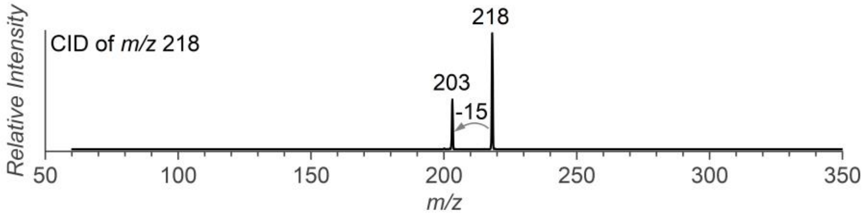

Figure S8. CID spectrum of the ions with *m/z* 218 at the normalized collision energy 30%, the only fragmentation was the loss of the methyl radical.

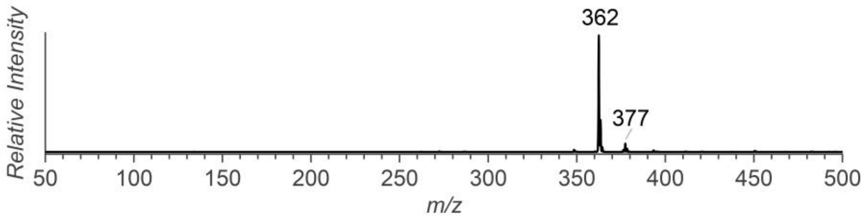

Figure S9. ESI-MS spectrum of the reaction mixture of THIQ (500 uM) in phenylacetone, 1 eq AcOH, under the oxygen atmosphere after 48 hours of reaction time. Aliquot of the reaction mixture was diluted with acetonitrile for the analysis.

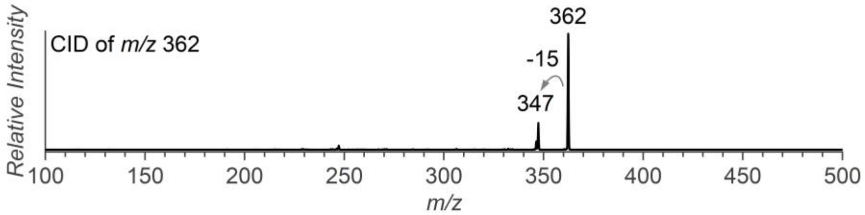

Figure S10. CID spectrum of the ions with *m/z* 362 at the normalized collision energy 28%.

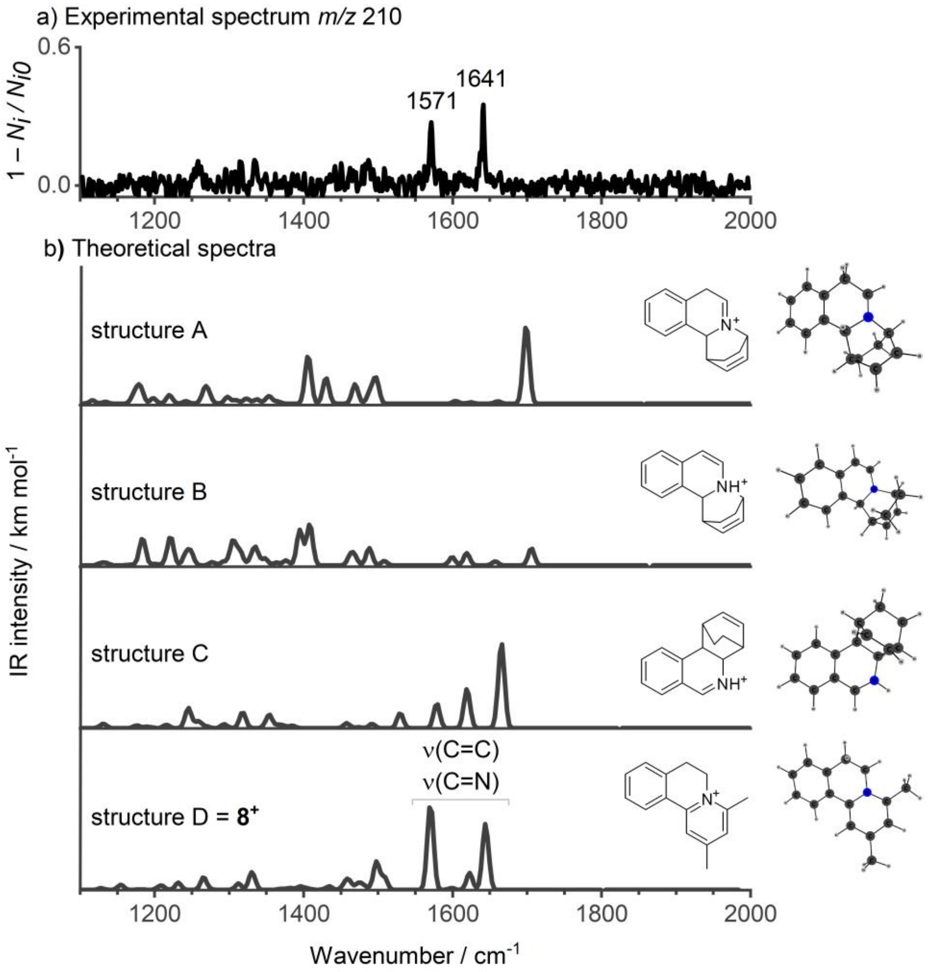

Figure S11. a) Helium tagging IRPD spectrum of the ion with *m/z* 210 measured at 3 K. b) Theoretical IR spectra calculated for different ions with *m/z* 210 (structure **A**, **B**, **C**, and $\mathbf{8}^{+}$), using B3LYP/6–31G**, and the scaling factor of 0.98.

Mechanistic study of the formation of peroxide 5

Procedure for the synthesis of peroxide **5**

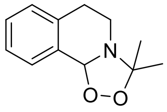

5

In a pressure tube equipped with a Teflon coated stirring bar, **THIQ** (67 mg, 0.5 mmol, 1.0 equiv.) and 1,2,4-triazole (35 mg, 0.5 mmol, 1.0 equiv.) were dissolved in acetone (5 mL, 0.1M) and 4Å molecular sieves (1.0 g) were added. The tube was charged with 300 mbar oxygen overpressure and stirred overnight. After releasing the pressure, the reaction volume was reduced using a continuous flow of air to 2 mL. The crude reaction mixture was then quickly filtered over a small plug of neutral AlO_x (packing the column was done with Et_2O, because if you use acetone it reacts with the stationary phase) to remove the acid. The filtrate was carefully dried under reduced pressure at ambient temperature. Product (0.048 g, 0.234 mmol) was obtained as a yellow oil in 47% yield.

^{1}H NMR (400 MHz, Acetone-*d6*) δ 7.36 – 7.29 (m, 2H, CH_{Ar}), 7.29 – 7.22 (m, 2H, CH_{Ar}), 5.86 (s, 1H, C$\underline{H}$OO), 3.16 – 3.10 (m, 1H, $CH_2CH\underline{H}N$), 2.97 – 2.76 (m, 3H, $C\underline{H}_2CH\underline{H}N$), 1.53 (s, 6H, CH_3).

^{13}C NMR (101 MHz, Acetone-*d6*) δ 136.9, 130.2, 129.8, 129.4, 128.8, 127.1, 100.8, 90.5, 41.5, 30.2, 28.1, 21.8.

HRMS (ESI+, *m/z*): calcd for $C_{12}H_{15}NO_2H^+$ $[M+H]^+$: 206.1176, found: 206.1163

Studying the involvement of the reactive oxygen species using mass spectrometry

In addition to the triplet oxygen used in the standard reaction conditions, we tested the involvement of various oxygen species by studying the reaction in the presence of KO_2 and $^1O_2$. The KO_2 is dissolved in acetonitrile and the reaction was performed similarly in the mixture of acetone and acetonitrile (9:1) under nitrogen. For the $^1O_2$ experiment, 1,4-dimethylnaphthalene-1,4-endoperoxide (NEP) was dissolved in methanol and the reaction mixture was heated to 60 ^{o}C under the nitrogen atmosphere.

The CID experiments demonstrate that under the standard reaction conditions with triplet oxygen, only the species **5H$^+$** is present (Figure S12a). For the experiment using KO_2 as oxygen source, a mixture of species **5H$^+$** and **5aH$^+$** is observed (Figure S12b). In addition, the predominant formation of the isomer **5aH$^+$** is observed when singlet oxygen is generated *in situ* by the thermal decomposition of 1,4-dimethylnaphthalene 1,4-endoperoxide. Please refer to the Scheme S1 for details on different fragmentation observed for **5H$^+$** and **5aH$^+$**.

This means that those reactive oxygen species are not responsible for the reactivity observed.

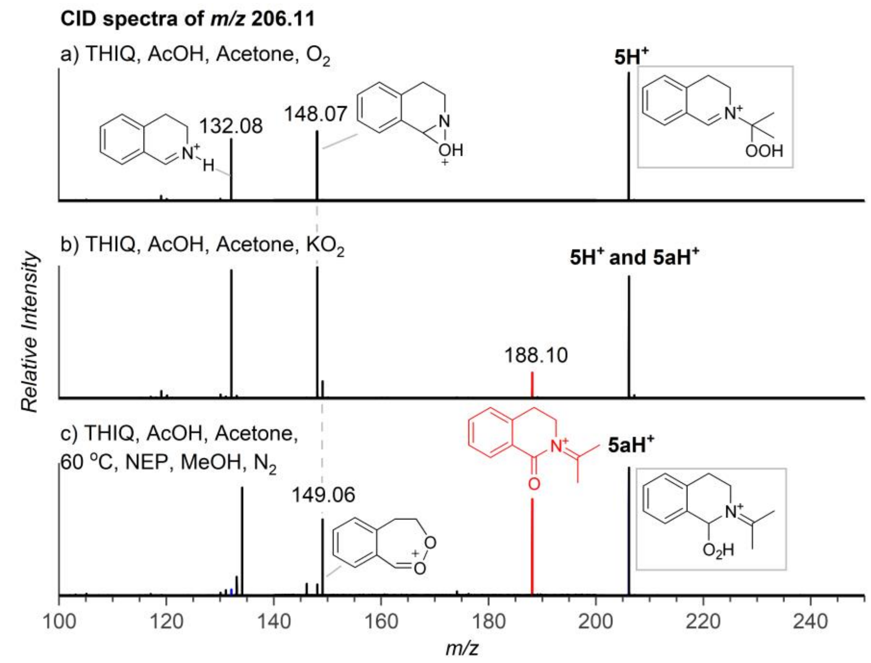

Figure S12. High-resolution CID spectra of *m/z* 206.11 under various reaction conditions: a) **THIQ**, AcOH in acetone under oxygen for 1 hr; b) **THIQ**, AcOH, KO_2 in the mixture of acetone and acetonitrile (9:1) under the nitrogen atmosphere; c) **THIQ**, AcOH, 1,4-dimethylnaphthalene-1,4-endoperoxide (NEP), acetone in methanol, 60 ^oC under the nitrogen atmosphere.

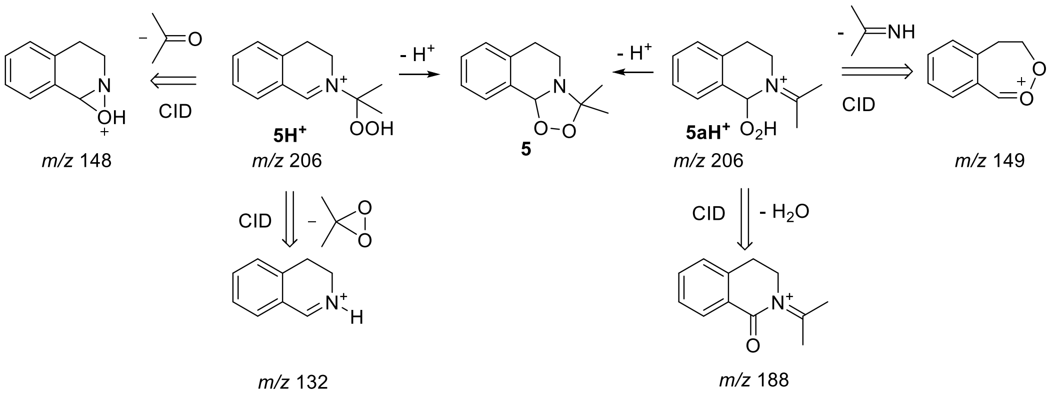

Scheme S1. Isomers of the ions with *m/z* 206 and the suggested fragmentation patterns.

<u>Measuring kinetic profiles of the reaction using NMR in flow</u>

To measure kinetics using NMR in flow, an InsightMR flowcell was combined with a peristaltic pump and a gas tight reaction flask (Figure S14). The flow rate of the peristaltic pump was set to 1 mL/min.

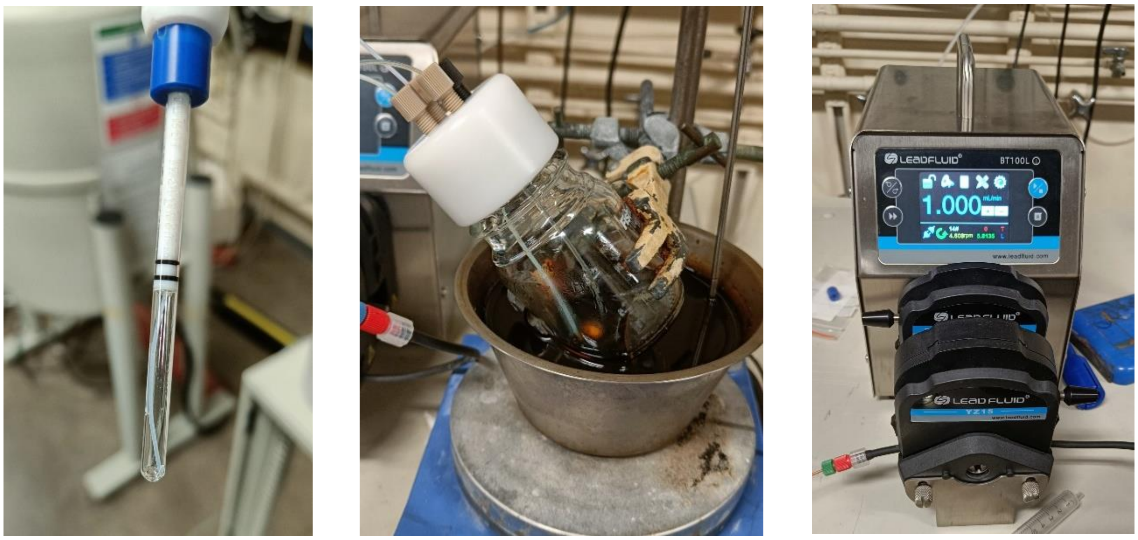

Figure S14. Set-up for NMR in flow. From left to right: NMR flow tube, reaction flask and peristaltic pump connected *via* microtubing.

General procedure for measuring the kinetics of the formation of peroxide **5**

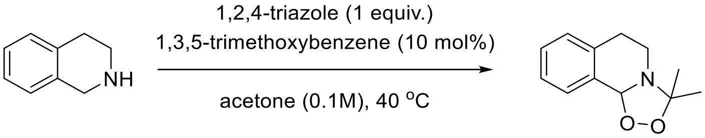

THIQ (133 mg, 1.0 mmol, 1.0 equiv.) and 1,3,5-trimethoxybenzene (internal standard, 16.8 mg, 0.1 mmol, 10 mol%) were dissolved in acetone (7.0 ml) and added to the reaction flask, which was equipped with a Teflon coated stirring bar. The flow (1 mL/min) was started and a ^{1}H NMR spectrum was recorded. The signal of acetone was set to ~2.2 ppm, after which the signal was suppressed using WET suppression. Then, a solution of 1,2,4-triazole (69 mg, 1.0 mmol, 1.0 equiv.) in acetone (3.0 mL) was added to the reaction flask, oxygen overpressure (300 mbar) was applied and the reaction was heated to 40 °C. From that moment, ^{1}H NMR spectra were recorded at set intervals (first two hours every 5 minutes, then every 15 minutes). Using MestReNova, the spectra were stacked, aligned, normalized over the internal standard and characteristic bands were integrated (Figure S15-S16):

- Y (red): Benzylic CH peroxide **5**: 5.90-5.97 ppm;
- Y1 (yellow): Benzylic CH_2 **THIQ** + iminium **4**$^{+}$: 3.92-4.09 ppm;
- Y2 (green): CH_3 iminium **4**$^{+}$: 1.93-1.98 ppm;
- Y3 (blue): CH_3 peroxide **5**: 1.54-1.65 ppm.

The amount of **THIQ** was determined by subtracting Y2 from Y1 and the data was plotted (Figure S17).

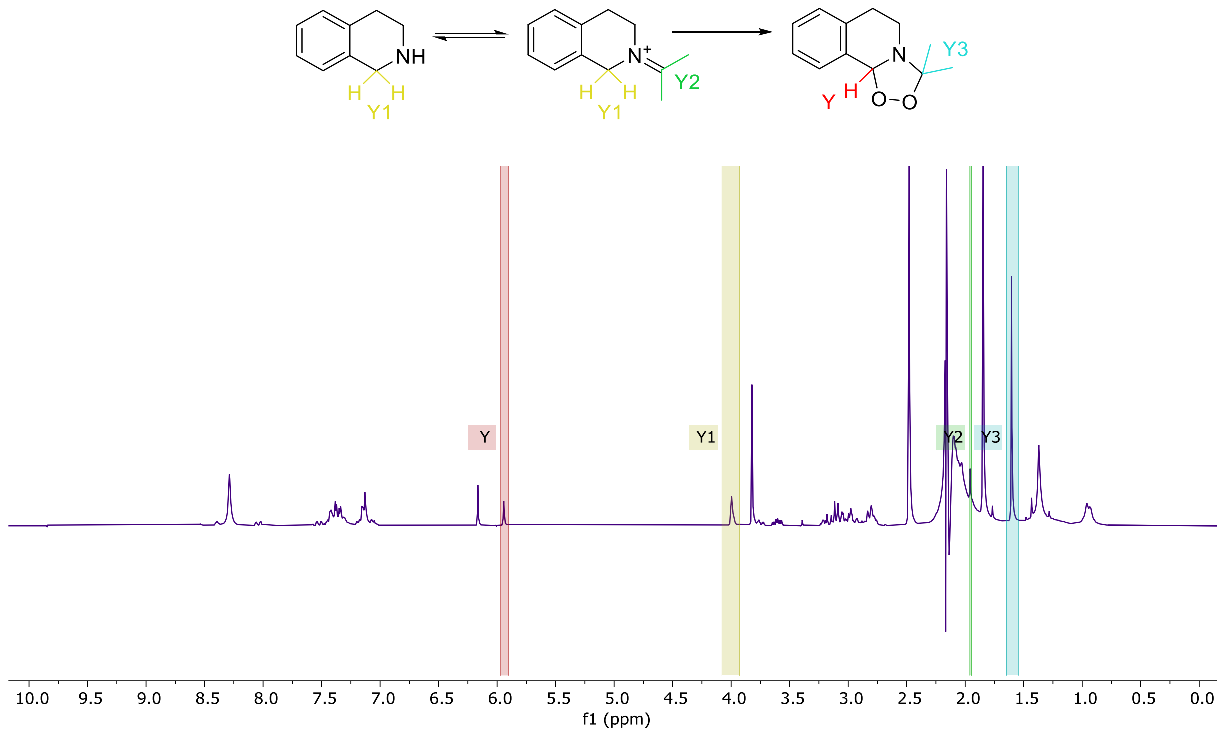

Figure S15. A typical ^{1}H NMR spectrum of the reaction mixture, with the characteristic peaks of **THIQ**, iminium **4**$^{+}$ and peroxide **5** highlighted.

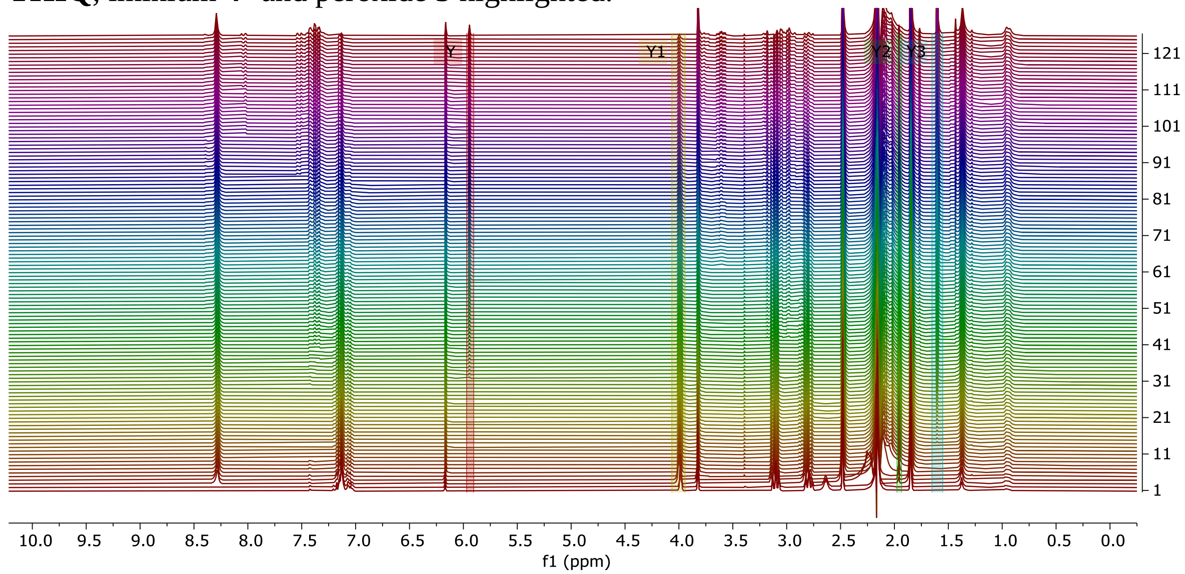

Figure S16. Array of ^{1}H NMR spectra after monitoring the kinetic profile of the reaction for 24 hours.

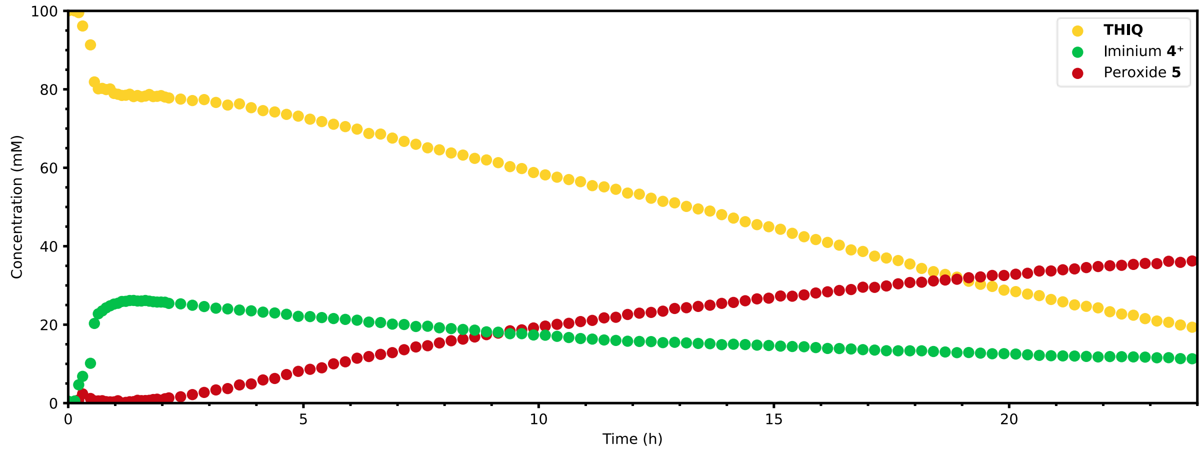

Figure S17. Plot of the conversion of starting materials to products from the integration of the array.

Irradiation of the reaction mixture with blue light

Following the general procedure for measuring kinetics, a reaction mixture was monitored over time using ^{1}H NMR in flow. After 18.5 hours, the reaction mixture was irradiated by placing a 455 nm blue LED close to the reaction flask. This resulted in a rapid increase in the rate of formation of peroxide **5** (Figure S18).

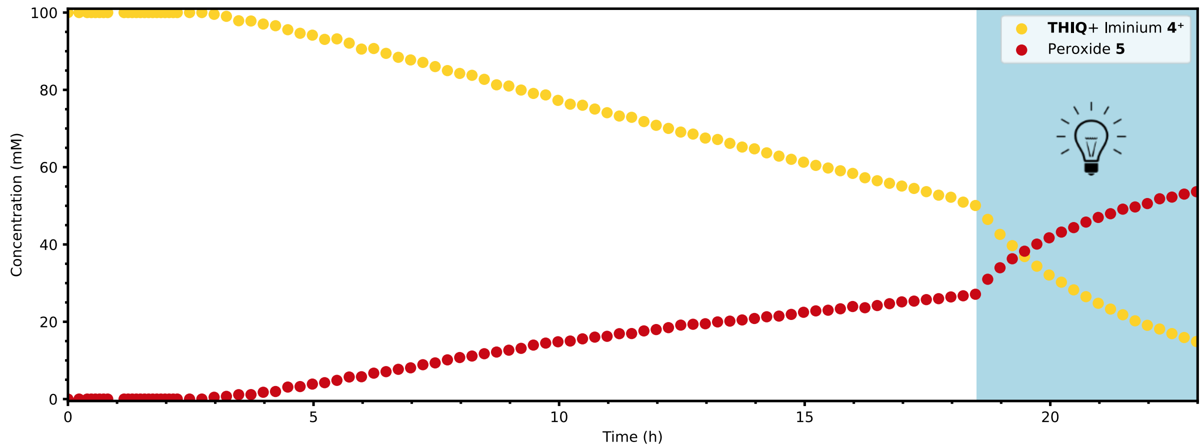

Figure S18. Plot of the conversion of starting materials to products from the integration of the array, featuring an increase in rate of formation of **5** upon irradiation with 455 nm LED.

Determination of the kinetic isotope effect of the benzylic position of **THIQ**

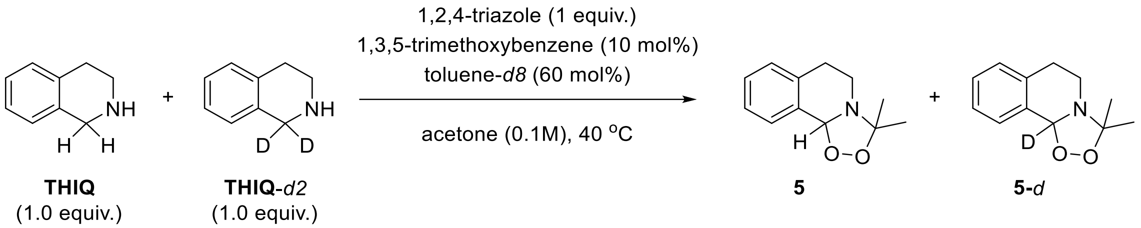

Following the general procedure for measuring kinetics, a reaction mixture was monitored over time using ^{1}H and ^{2}H NMR. In addition to **THIQ**, **THIQ**-*d2* (1.0 equiv.) was added to the reaction mixture as well as toluene-*d8* (60 mol%) as internal standard for the ^{2}H NMR spectra. The rate of consumption of substrates was measured as the combined integration of **THIQ** + iminium **4**$^+$ and **THIQ**-*d2* + iminium-*d2* **4**$^+$ respectively (Figure S19 and S20).

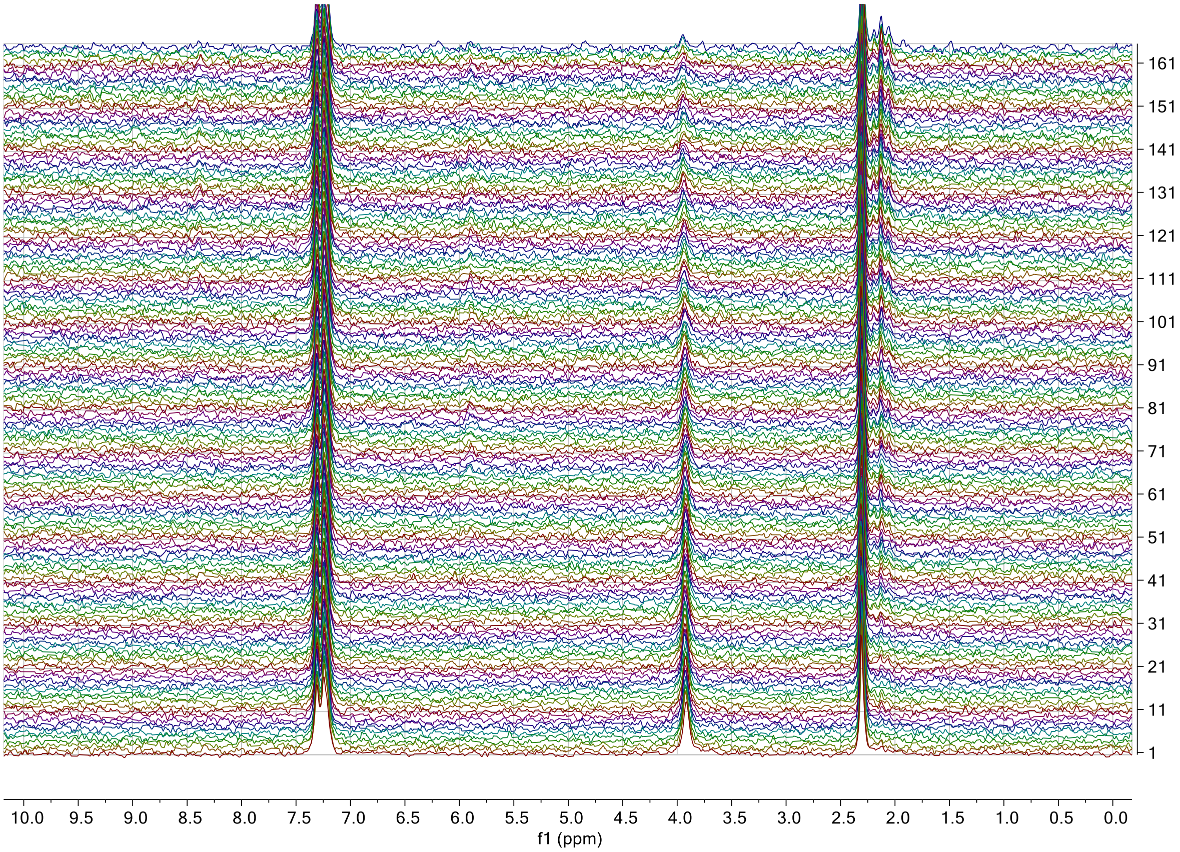

Figure S19. Array of ^{2}H NMR spectra after monitoring the kinetic profile of the reaction for 40 hours.

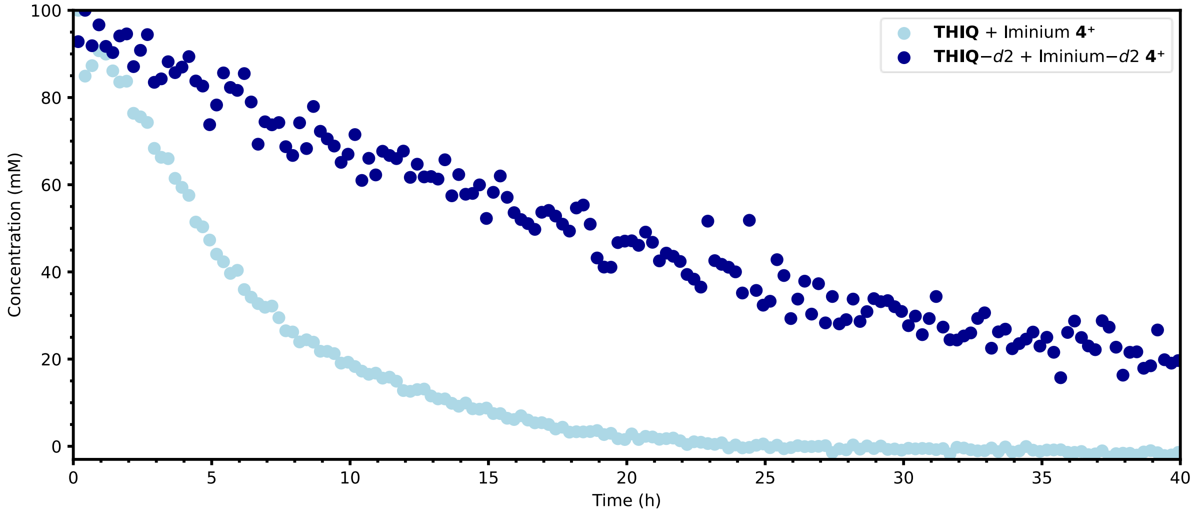

Figure S20. Plot of the rate of consumption of substrates **THIQ** and **THIQ**-*d2* over time.

After taking the natural logarithm of the two data sets, linear functions were observed (Figure S21), and the slopes were divided (-0.1761/-0.0358). This led to a k_H/k_D of 4.9.

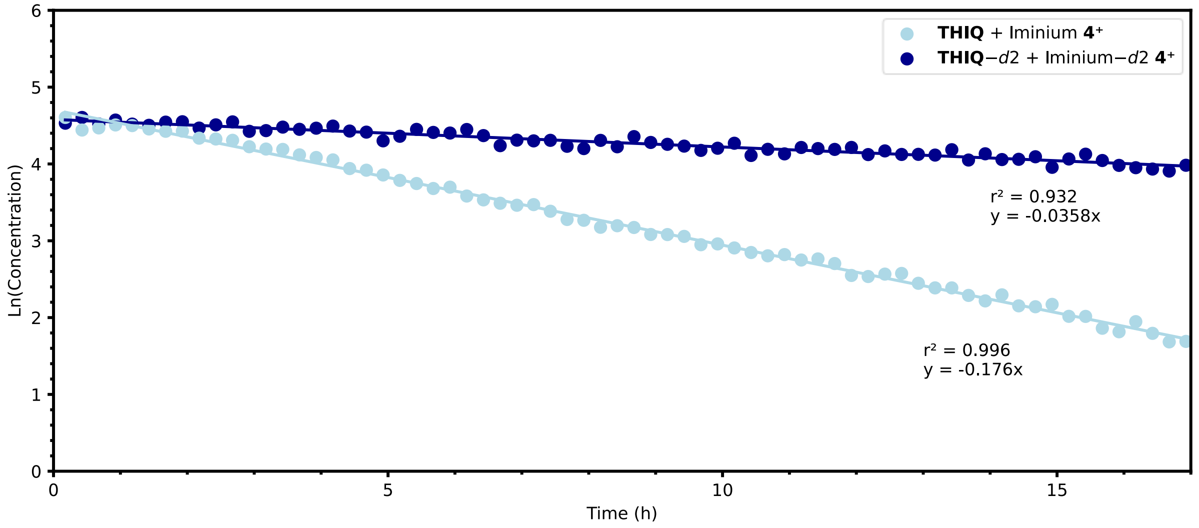

Figure S21. Plot of the logarithmic rate of consumption of substrates **THIQ** and **THIQ**-*d2* over time plus trendline.

Determination of the reaction order in **THIQ**

Following the general procedure for measuring kinetics, a reaction mixture was monitored over time using ^{1}H NMR in flow. A second run was then recorded, using 0.5 equivalent of **THIQ** instead of 1.0 equivalent. Using variable time normalization analysis (VTNA),[10] the two curves were plotted, and the rate order was adjusted until a good overlap was observed. The best overlap was observed when the order of **THIQ** was set to 0.5 (Figure S22).

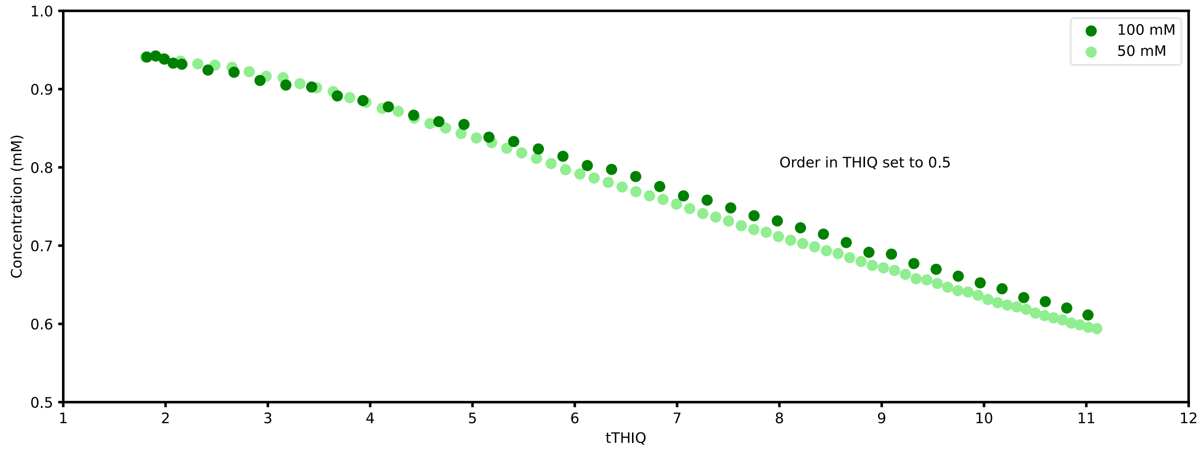

Figure S22. Plot of the overlap of two experiments using VTNA when the rate order was set to 0.5.

Detection of enamine **13** using scrambling experiments

To probe for an EDA complex consisting of iminium **4**$^+$ and enamine **13** (Scheme S2), we first set out to detect enamine **13**.

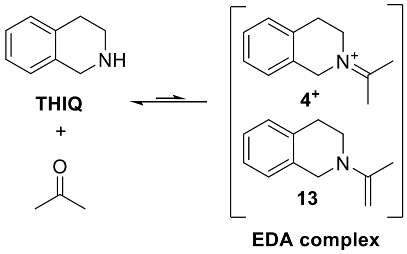

Scheme S2. Proposed nature of the EDA complex.

Following the general procedure for measuring kinetics, a reaction mixture was monitored over time using ^{1}H NMR in flow. Instead of acetone as a solvent, acetone-*d6* was employed. If enamine **13** is present in the reaction mixture, this would lead to scrambling of deuterium and incorporation of protons both in iminium-*d6* **4**$^+$ and the resulting product peroxide **5**-*d6* (Scheme S3). Indeed, over the course of the reaction scrambling of the deuterium signals was observed, confirming the presence of enamine **13** in the reaction mixture (Figure S23).

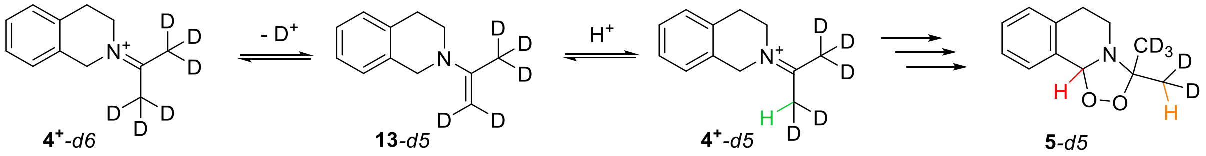

Scheme S3. Scrambling of deuteriums due to an iminium-enamine tautomerization.

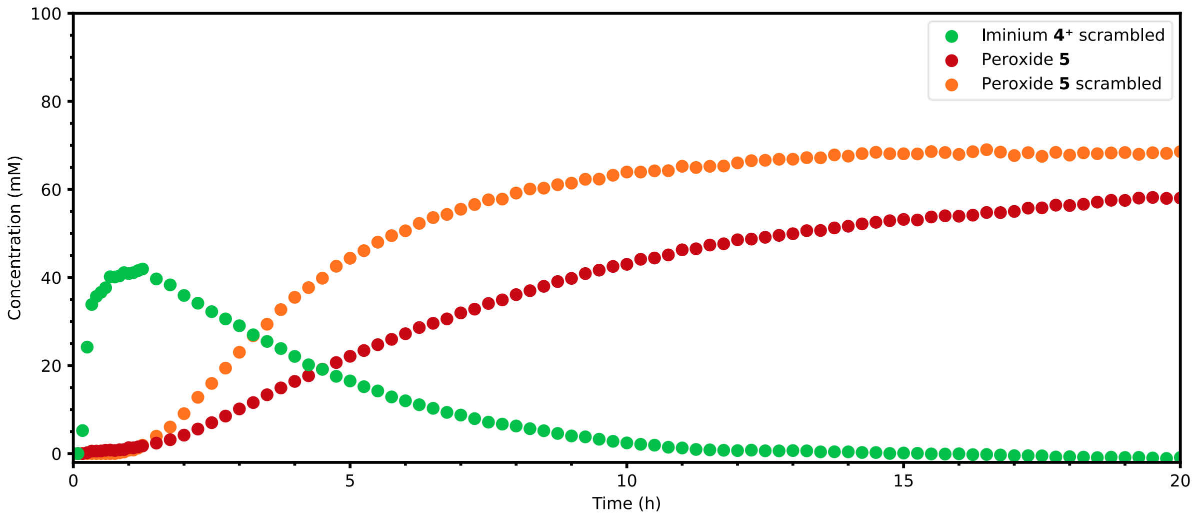

Figure S23. Plot of the formation of scrambled species in the reaction mixture. Iminium **4**$^+$ scrambled indicates the observation of a proton in the methyl group of the species as a result of scrambling. Peroxide **5** indicates the benzylic proton of the species, which is independent of scrambling. Peroxide **5** scrambled indicates the observation of protons in the methyl groups of the species as a result of scrambling.

This finding could also be corroborated by mass spectrometric measurements, where the formation of pyridinium **8**$^+$ in acetone-*d6* yielded a distribution of products with different levels of H-atom incorporation. This is also consistent with D/H scrambling and, thus the formation of enamine **13** under the reaction conditions. The Figure S24a-d shows a zoomed section of the high-resolution mass spectra of reaction mixtures after 24 hours under the oxygen atmosphere, while Figure S24e shows the calculated isotopic pattern for **8**$^+$ and its completely deuterated analogue.

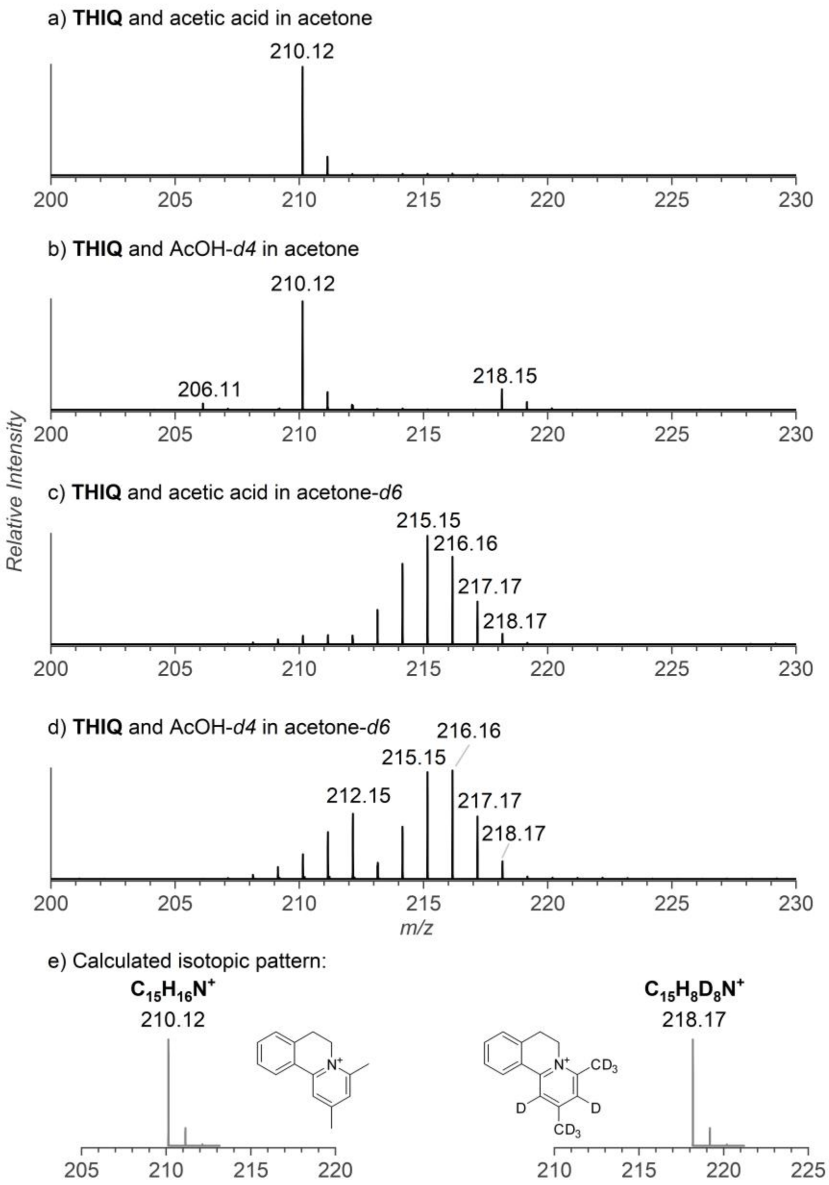

Figure S24. ESI-TOF spectra (zoomed section at around *m/z* 210, for the **8**$^+$) of the specified reaction mixture after 24 hours under the oxygen atmosphere: a) **THIQ** and acetic acid in acetone, b) **THIQ** and AcOH-*d4* in acetone, c) **THIQ** and acetic acid in acetone-*d6*, and d) **THIQ** and AcOH-*d4* in acetone-*d6*.

<u>Detection of the EDA complex by mass spectrometry</u>

If the EDA complex consisting of iminium **4**$^+$ and enamine **13** were formed in the solution, we expected it to be detectable by mass spectrometry. Indeed, we could detect the corresponding ions, albeit their abundance was very low (Figure S25). The top panel shows **THIQ** (2 mM) in acetone under an oxygen atmosphere followed by 1 equivalent of AcOH addition, revealing the appearance of *m/z* 347.2486. The bottom panel shows the calculated isotopic pattern of EDA complex with composition $C_{24}H_{31}N_2^+$ at *m/z* 347.2482.

The relative intensity of specified ions corrected by the total ion chromatogram was plotted over time. Due to the very low intensity of the EDA complex, the ion trace has been magnified 500 times. This plot demonstrates that the EDA complex (black) appears slightly before **5H⁺** (red), indicating its intermediacy in the formation of **5**.

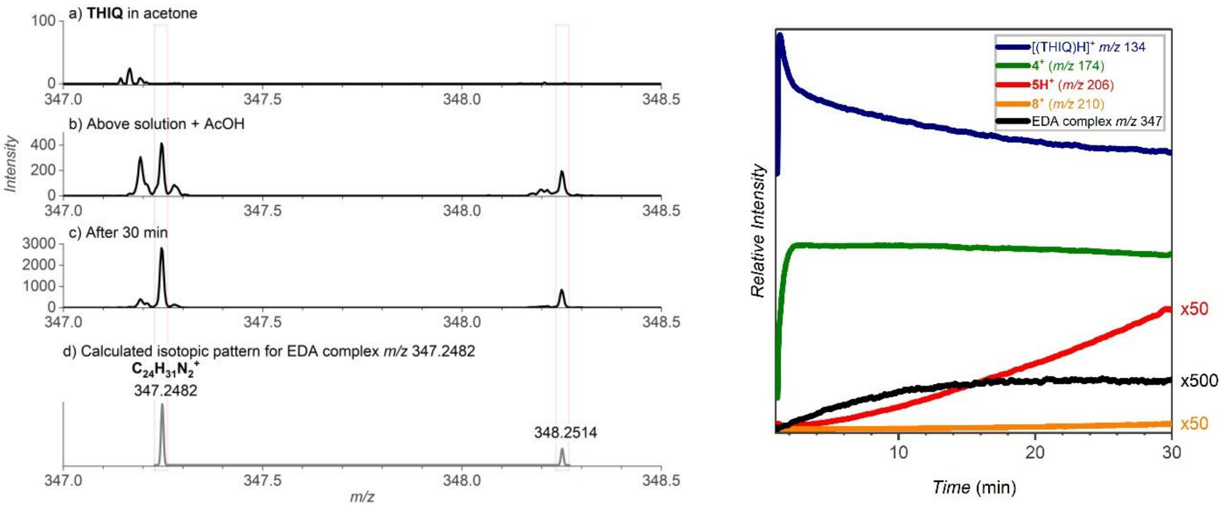

Figure S25. Left: ESI-MS spectra (zoomed section at *m/z* 347, for the EDA complex) of the reaction mixture under the dark conditions: a) **THIQ** in acetone (2 mM), b) 1 equivalent AcOH added, c) after 30 min, d) simulated isotopic pattern; Right: Plot of the specified ion relative intensity over time during the reaction. Traces are as follows- blue: [(**THIQ**)H]$^+$, green: **4**$^+$, red: **5H**$^+$, orange: **8**$^+$ and black: EDA complex.

CID of the selected ions with *m/z* 347 revealed the loss of enamine **13** at low collision energy (Figure S26). This is consistent with a non-covalent interaction between the two species of the EDA-complex.

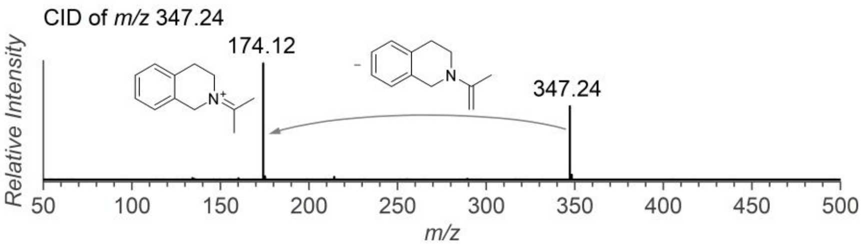

Figure S26. CID spectrum of *m/z* 347.24 at collision energy 4 eV, showing the loss of enamine **13** to iminium **4**$^+$.

Screening of conditions for the formation of pyridinium 8$^+$

For optimization of the reaction conditions, **THIQ** (27 mg, 0.2 mmol, 1.0 equiv.), acetone (0.29 mL, 4.0 mmol, 20 equiv.) the corresponding acid (0.2 mmol, 1.0 equiv.) and solvent (2.0 mL, 0.1M) were stirred overnight at room temperature under one atmosphere of oxygen in the presence of 1,3,5-trimethoxybenzene (internal standard, 3.4 mg, 0.02 mmol, 10 mol%). After 16 hours, a

sample was taken and the conversion and yield of the reaction components were determined by ^{1}H NMR.

It was found that the acid and solvent were leading in determining product distributions and conversions.

Screening of Brønsted acids:

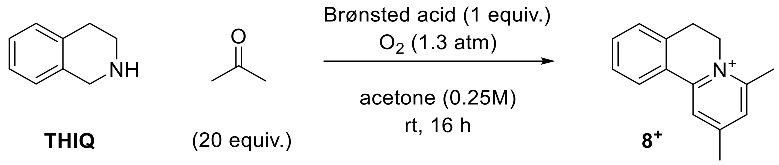

Entry	Acid	pK_a	Conversion	Peroxide 5	Adduct 2	Pyridinium 8^+	Remarks
**1**	1,2,4-Triazole	10.4	61%	23%	-	**-**	
**2**	Phenol	10	31%	3%	-	**1%**	
**3**	1,2,3-Triazole	9.4	58%	17%	-	**-**	
**4**	p-NO_2-PhOH	7.2	100%	-	6%	**7%**	
**5**	Tetrazole	4.9	93%	-	9%	**10%**	
**6**	AcOH	4.8	85%	-	11%	**-**	
**7**	Toluic acid	4.2	40%	-	4%	**-**	Poor solubility of acid
**8**	Toluic acid	4.2	92%	20%	6%	**-**	0.05M
**9**	Formic acid	3.8	88%	-	7%	**1%**	
**10**	HCl	-5.9	-	-	-	**-**	Precipitation of substrate

Table S3. Screening of the Brønsted acids for the formation of pyridinium **8**$^+$

Screening of solvents:

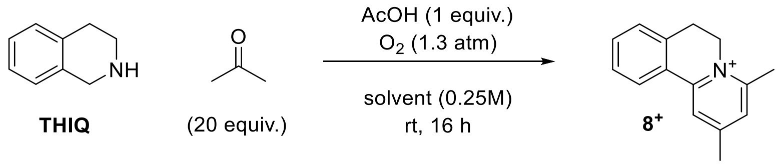

Entry	Solvent	Conversion	Peroxide 5	Adduct 2	Pyridinium 8^+	Remarks
**1**	Acetone	85%	-	11%	**-**	
**2**	THF	0	-	-	**-**	
**3**	DCM	38%	-	-	**-**	
**4**	Dioxane	70%	-	-	**-**	
**5**	MeOH	58%	-	-	**12%**	
**6**	DMF	>95%	4	-	**-**	22% 3,4-dihydroisoquinolin-1(2*H*)-one

Table S4. Screening of solvents for the formation of pyridinium **8**$^+$

Synthesis of substrates

Synthesis of 6,7-dioxymethylene-1,2,3,4-tetrahydroisoquinoline (**THIQe**)

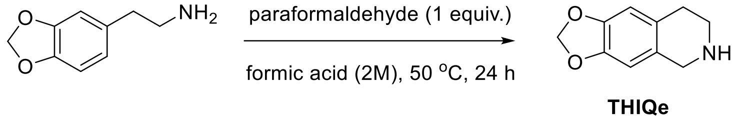

Product was synthesized according to a literature procedure.[7] To a reaction flask equipped with a Teflon coated stirring egg, 1,3-benzodioxole-5-ethanamine (608 mg, 3.68 mmol, 1.0 equiv.) and formic acid (1.8 mL, 2M) were added at 0 °C until complete dissolution of the amine. Paraformaldehyde (111 mg, 3.68 mmol, 1.0 equiv.) was added and the reaction was heated to 50 °C for 24 hours. The solution was cooled to 0 °C before an aqueous NaOH solution (saturated, 5 mL) was added dropwise. The aqueous layer was extracted with DCM (3x5 mL) and the combined organic layers were dried ($MgSO_4$), filtered and concentrated. The crude product was purified by column chromatography (SiO_2, DCM/MeOH, 30:1). Product (254 mg, 1.43 mmol) was obtained as a white solid in 39% yield.

^{1}H NMR (400 MHz, Chloroform-*d*) δ 6.56 (s, 1H, CH_{Ar}), 6.48 (s, 1H, CH_{Ar}), 5.89 (s, 2H, OCH_2O), 3.94 (s, 2H, CH_2), 3.12 (t, J = 6.0 Hz, 2H, CH_2), 2.73 (t, J = 6.0 Hz, 2H, CH_2), 1.70 (s, 1H, NH).

Synthesis of **THIQ**-*d2*

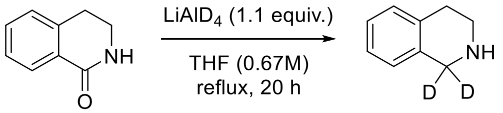

Product was synthesized according to a literature procedure.[8] To a heat dried reaction flask equipped with a Teflon coated stirring egg, $LiAlD_4$ (229 mg, 5.45 mmol, 1.1 equiv.) and dry THF (1.5 mL) were added and the resulting suspension was cooled to 0 °C. A solution of 3,4-dihydroisoquinolin-1(2*H*)-one (729 mg, 4.95 mmol, 1.0 equiv.) in dry THF (5 mL, 1M) was added and the mixture was heated to reflux. After 20 hours, the mixture was cooled to 0 °C. Water (20 mL) was then slowly added, together with an aqueous NaOH solution (10-15 mL, 1M). The product was extracted with Et_2O (3x30 mL) and the combined organic layers were dried (Na_2SO_4). After evaporation of the solvent, product (568 mg, 4.2 mmol, >95% D-incorporation) was obtained as a yellow oil in 85% yield.

^{1}H NMR (600 MHz, Chloroform-*d*) δ 7.16-7.06 (m, 3H, CH_{Ar}), 7.03-6.99 (m, 1H, CH_{Ar}), 3.14 (t, J = 6.0 Hz, 2H, CH_2), 2.8 (t, J = 6.0 Hz, 2H, CH_2), 1.69 (bs, 1H, NH).

^{13}C NMR (151 MHz, Chloroform-*d*): δ 136.1, 135.0, 129.5, 126.4, 126.2, 125.9, 47.8 (p, J = 20.7 Hz), 44.0, 29.4.

Synthesis of N-cyclohexyl-2-{[(2,2,6,6-tetramethylpiperidin-1-yl)oxy]methyl}acrylamide (CHANT)

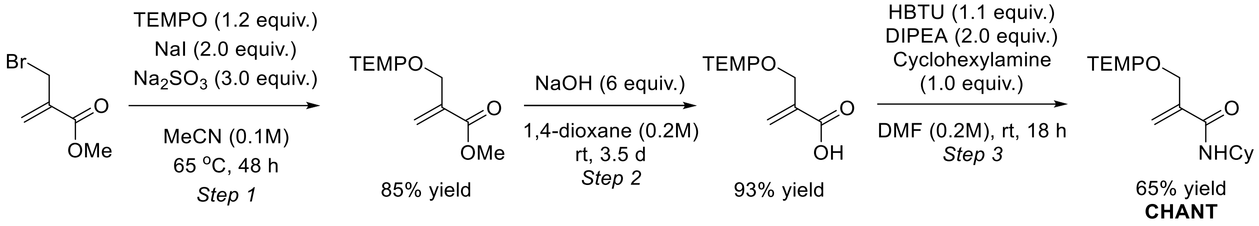

Product was synthesized according to a literature procedure.[9]

Step 1:
To a reaction flask equipped with a Teflon coated stirring egg, methyl 2-(bromomethyl)acrylate (1.43 g, 8.0 mmol, 1.0 equiv.), (2,2,6,6-tetramethylpiperidin-1-yl)oxyl (TEMPO, 1.51 g, 9.6 mmol, 1.2 equiv.), NaI (2.40 g, 16.0 mmol, 2.0 equiv.) and Na_2SO_3 (3.03 g, 24.0 mmol, 3.0 equiv.) and dry MeCN (80 mL, 0.1 M) were added under an inert atmosphere. The mixture was heated to 65 °C and stirred for 48 hours. After the solvent was removed, H_2O (100 mL) was added and the aqueous phase was extracted with EtOAc (3×100 mL). The combined organic phases were dried ($MgSO_4$), filtered and concentrated. The crude product was purified using column chromatography (SiO_2, DCM/Et_2O, 50:1). Product (1.73 g, 6.8 mmol) was obtained as a yellow oil in 85% yield.

^{1}H NMR (400 MHz, Chloroform-*d*) δ 6.28 (dt, *J* = 1.8, 1.6 Hz, 1H, C=C<u>H</u>H), 5.91 (dt, *J* = 2.0, 1.8 Hz, 1H, C=CH<u>H</u>), 4.50 (dd, *J* = 2.0, 1.6 Hz, 2H, C<u>H</u>$_2$C=CH$_2$), 3.75 (s, 3H, OCH$_3$), 1.66 – 1.50 (m, 1H, C<u>H</u>H), 1.50 – 1.40 (m, 4H, CH$_2$), 1.39 – 1.26 (m, 1H, CH<u>H</u>), 1.17 (s, 6H, CH$_3$), 1.12 (s, 6H, CH$_3$).

Step 2:
The ester obtained in the first step (1.73 g, 6.78 mmol, 1.0 equiv.) was added to a reaction flask equipped with a Teflon coated stirring egg. 1,4-Dioxane (40 mL, ~0.2M) and aqueous NaOH (40 mL, 1M, 6 equiv.) were added and the solution was stirred for 3.5 days. The mixture was then acidified with aqueous HCl (20 mL, 2.0M, 6.5 equiv.) and the aqueous phase was extracted with EtOAc (3×40 mL). The combined organic phases were dried ($MgSO_4$), filtered and concentrated. Product (1.52 g, 6.3 mmol) was obtained as a yellow oil in 93% yield.

^{1}H NMR (400 MHz, Chloroform-*d*) *δ* 6.40 (s, 1H, C=CH<u>H</u>), 5.96 (s, 1H, C=C<u>H</u>H), 4.52 (s, 2H, C<u>H</u>$_2$C=CH$_2$), 1.67 – 1.41 (m, 6H, CH$_2$), 1.19 (s, 6H, CH$_3$), 1.14 (s, 6H, CH$_3$).

Step 3:
The carboxylic acid obtained in the second step (200.8 mg, 0.83 mmol, 1.0 equiv.) was added to a reaction flask equipped with a Teflon coated stirring egg. HBTU (347.1 mg, 0.915 mmol, 1.1 equiv.), DIPEA (290 µL, 1.66 mmol, 2.0 equiv.) and cyclohexylamine (95 µL, 0.83 mmol, 1.0 equiv.) and dry DMF (4.2 mL, 0.2M) were added and the reaction mixture was stirred for 18 hours. After the solvent was removed, aqueous $NaHCO_3$ (saturated, 8 mL) was added and the aqueous phase was extracted with EtOAc (3×8 mL). The combined organic layers dried with $MgSO_4$, filtered and concentrated. The crude product was purified using column chromatography (SiO_2, pentane/EtOAc, 8:1). Product (174 mg, 0.54 mmol) was obtained as a white solid in 65% yield.

^{1}H NMR (Chloroform-*d*, 400 MHz) δ 6.61 – 6.57 (m, 1H, OCNH), 6.08 (d, J = 1.8 Hz, 1H, C=C<u>H</u>H), 5.48 (s, 1H, C=CH<u>H</u>), 4.48 (s, 2H, CH_2O), 3.87 – 3.81 (m, 1H, C<u>H</u>NH), 2.09 – 1.94 (m, 2H, CH), 1.78 – 1.70 (m, 2H, CH), 1.68 – 1.60 (m, 1H, CH), 1.51 – 1.44 (m, 5H, CH), 1.43 – 1.32 (m, 3H, CH), 1.19 (s, 6H, CH_3), 1.17 – 1.14 (m, 3H, CH), 1.12 (s, 6H, CH_3).

Attempted ketones

Numerous ketones have been tried in the reaction protocol. The ketones highlighted in green led to desired product formation.

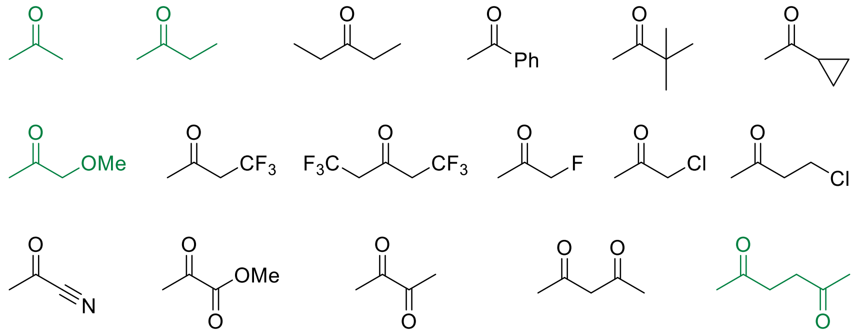

Figure S27. Ketones that were tested in the reaction protocol

Synthesis of pyridinium products 8$^+$

<u>General procedure for synthesis of pyridinium products **8**$^+$</u>

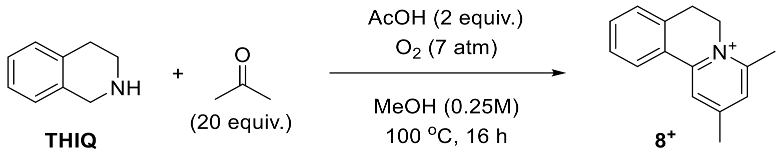

In a pressure vessel equipped with a Teflon coated stirring bar, the corresponding **THIQ** (1.0 equiv.), the corresponding ketone (20 equiv.) and AcOH (2 equiv.) were dissolved in anhydrous methanol (0.25M). The vessel was charged with 7 bars of oxygen overpressure and heated to 100 °C. The reaction was monitored by GC MS and after full consumption of the substrate, the reaction was allowed to reach ambient pressure and temperature (typically within 24 hours). After the volatiles were removed under vacuum, the crude was dissolved in DCM (~15 mL) and the organic layer was extracted with water (3x 10 mL). The combined aqueous layers were concentrated under vacuum at 65 °C. DCM was added and the solution was filtered to remove final impurities. The product was obtained after concentration.

<u>Characterization</u>

2,4-Dimethyl-6,7-dihydropyrido[2,1-a]isoquinolin-5-ium diacetate (**8a**$^+$)

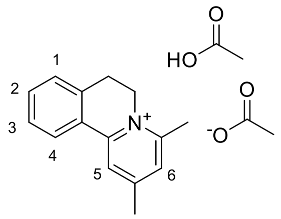

8a+

Reaction was performed starting from **THIQ** (266 mg, 2.0 mmol) and acetone (2.9 mL, 40.0 mmol). Product **8a**$^+$ (439 mg, 1.34 mmol) was obtained as a brown oil in 67% yield.

^{1}H NMR (400 MHz, Chloroform-*d*) δ 7.90 (s, 1H, $CH_{Ar(5)}$), 7.88 (d, *J* = 7.5 Hz, 1H, $CH_{Ar(4)}$), 7.63 – 7.51 (m, 2H, $CH_{Ar(3,6)}$), 7.47 (t, *J* = 7.5 Hz, 1H, $CH_{Ar(2)}$), 7.40 (d, *J* = 7.5 Hz, 1H, $CH_{Ar(1)}$), 4.80 (t, *J* = 6.7 Hz, 2H, CH_2), 3.34 (t, *J* = 6.7 Hz, 2H, CH_2), 3.03 (s, 3H, CH_3), 2.63 (s, 3H, CH_3), 1.93 (s, 6H, CH_3).

^{13}C NMR (101 MHz, Chloroform-*d*) δ 176.3, 157.7, 155.1, 149.1, 136.1, 133.5, 128.7 (2x), 128.5, 126.9, 126.7, 122.8, 49.0, 26.9, 23.2, 22.2, 22.1.

HRMS (ESI+, *m/z*): calcd for $C_{15}H_{16}N^+$ [M$^+$]: 210.1277, found: 210.1272.

2,4,10-Trimethyl-6,7-dihydropyrido[2,1-a]isoquinolin-5-ium diacetate (**8b**$^+$)

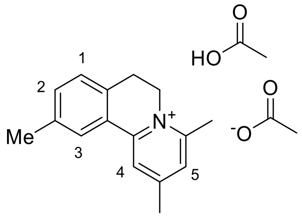

8b+

Reaction was performed starting from 7-methyl-**THIQ** (74 mg, 0.5 mmol) and acetone (0.74 mL, 10.0 mmol). Product (81 mg, 0.237 mmol) was obtained as a brown oil in 47% yield.

^{1}H NMR (400 MHz, Chloroform-*d*) δ 7.89 (s, 1H, $CH_{Ar(4)}$), 7.66 (s, 1H, $CH_{Ar(3)}$), 7.54 (s, 1H, $CH_{Ar(5)}$), 7.36 (d, *J* = 7.5 Hz, 1H, $CH_{Ar(1)}$), 7.27 (d, *J* = 7.5 Hz, 1H, $CH_{Ar(2)}$), 4.80 (t, *J* = 6.5 Hz, 2H, CH_2), 3.29 (t, *J* = 6.5 Hz, 2H, CH_2), 3.06 (s, 3H, CH_3), 2.62 (s, 3H, CH_3), 2.43 (s, 3H, CH_3), 1.91 (s, 6H, CH_3).

^{13}C NMR (101 MHz, Chloroform-*d*) δ 175.8, 157.7, 154.8, 149.2, 138.6, 134.3, 132.9, 128.6, 128.3, 127.3, 126.5, 122.7, 49.1, 26.4, 22.9, 22.1, 22.0, 21.4.

HRMS (ESI+, *m/z*): calcd for $C_{16}H_{18}N^+$ [M$^+$]: 224.1434, found: 224.1434.

9-Methoxy-2,4-dimethyl-6,7-dihydropyrido[2,1-a]isoquinolin-5-ium diacetate (**8c**$^+$)

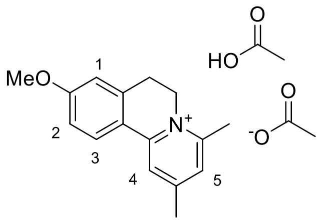

8c⁺

Reaction was performed starting from 6-methoxy-**THIQ** (103 mg, 0.63 mmol) and acetone (0.93 mL, 12.62 mmol). Product (102 mg, 0.285 mmol) was obtained as a brown oil in 45% yield.

^{1}H NMR (400 MHz, Chloroform-*d*) δ 7.84 – 7.78 (m, 2H, $CH_{Ar(3+4)}$), 7.40 (s, 1H, $CH_{Ar(5)}$), 6.96 (d, *J* = 8.8 Hz, 1H, $CH_{Ar(2)}$), 6.88 (s, 1H, $CH_{Ar(1)}$), 4.81 (t, *J* = 6.6 Hz, 2H, CH_2), 4.76 (s, 3H, CH_3), 3.90 (bs, 1H, O<u>H</u>O), 3.33 (t, *J* = 6.4 Hz, 2H, CH_2), 3.03 (s, 3H, CH_3), 2.59 (s, 3H, CH_3), 1.93 (s, 6H, CH_3).

^{13}C NMR (151 MHz, Chloroform-*d*) δ 176.4, 163.8, 157.0, 154.4, 149.4, 138.6, 129.0, 127.3, 121.7, 119.1, 115.2, 113.0, 55.9, 48.9, 27.3, 23.6, 22.2, 22.1.

HRMS (ESI+, *m/z*): calcd for $C_{16}H_{18}NO^+$ [M^+]: 240.1383, found: 240.1381.

9,10-Dimethoxy-2,4-dimethyl-6,7-dihydropyrido[2,1-a]isoquinolin-5-ium diacetate (**8d⁺**)

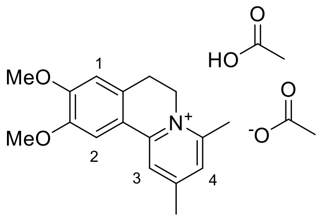

8d⁺

Reaction was performed starting from 6,7-dimethoxy-**THIQ** (97 mg, 0.5 mmol) and acetone (0.74 mL, 10.0 mmol). Product (97 mg, 0.250 mmol) was obtained as a brown oil in 50% yield.

^{1}H NMR (400 MHz, Chloroform-*d*) δ 8.04 (s, 1H, $CH_{Ar(3)}$), 7.58 (bs, 1H, O<u>H</u>O), 7.41 – 7.35 (m, 2H, $CH_{Ar(2+4)}$), 6.80 (s, 1H, $CH_{Ar(1)}$), 4.75 – 4.59 (m, 2H, CH_2), 3.97 (s, 3H, CH_3), 3.93 (s, 3H, CH_3), 3.30 – 3.12 (m, 2H, CH_2), 2.94 (s, 3H, CH_3), 2.58 (s, 3H, CH_3), 1.94 (s, 6H, CH_3).

^{13}C NMR (101 MHz, Chloroform-*d*) δ 176.6, 157.5, 153.8, 153.7, 149.4, 149.0, 130.2, 127.3, 122.4, 118.6, 110.5, 109.6, 56.9, 56.4, 48.8, 26.5, 23.4, 22.1, 22.0.

HRMS (ESI+, *m/z*): calcd for $C_{17}H_{20}NO_2^+$ [M^+]: 270.1489, found: 270.1487.

2,4-Dimethyl-6,7-dihydro-[1,3]dioxolo[4,5-g]pyrido[2,1-a]isoquinolin-5-ium diacetate (**8e⁺**)

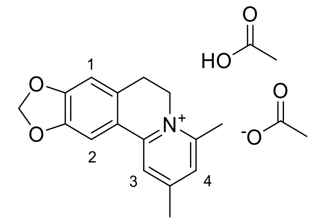

8e⁺

Reaction was performed starting from 6,7-methylenedioxy-**THIQ** (89 mg, 0.5 mmol) and acetone (0.74 mL, 10.0 mmol). Product (78 mg, 0.209 mmol) was obtained as a brown oil in 41% yield.

^{1}H NMR (400 MHz, Chloroform-*d*) δ 7.69 (s, 1H, $CH_{Ar(3)}$), 7.42 (s, 1H, $CH_{Ar(4)}$), 7.27 (s, 1H, $CH_{Ar(2)}$), 6.83 (s, 1H, $CH_{Ar(1)}$), 6.09 (s, 2H, CH_2), 4.80 (t, *J* = 6.5 Hz, 2H, CH_2), 3.26 (t, *J* = 6.5 Hz, 2H, CH_2), 3.11 (bs, 1H, O<u>H</u>O), 3.04 (s, 3H, CH_3), 2.59 (s, 3H, CH_3), 1.92 (s, 6H, CH_3).

^{13}C NMR (101 MHz, Chloroform-*d*) δ 176.6, 157.2, 154.8, 152.2, 149.0, 148.6, 132.8, 127.6, 121.9, 108.5, 106.3, 102.5, 48.9, 29.8, 27.0, 23.9, 22.2 (2x).

HRMS (ESI+, *m/z*): calcd for $C_{16}H_{16}NO_2^+$ [M^+]: 254.1176, found: 254.1173.

8-Bromo-2,4-dimethyl-6,7-dihydropyrido[2,1-a]isoquinolin-5-ium diacetate (**8f⁺**)

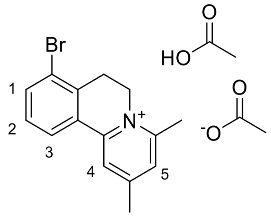

8f⁺

Reaction was performed starting from 5-bromo-**THIQ** (106 mg, 0.5 mmol) and acetone (0.74 mL, 10.0 mmol). Product (100 mg, 0.246 mmol) was obtained as a black oil in 49% yield.

^{1}H NMR (400 MHz, Chloroform-*d*) δ 7.93 (s, 1H, $CH_{Ar(4)}$), 7.90 (d, *J* = 8.4 Hz, 1H, $CH_{Ar(1)}$), 7.79 (d, *J* = 7.9 Hz, 1H, $CH_{Ar(3)}$), 7.58 (s, 1H, $CH_{Ar(5)}$), 7.37 (dd, *J* = 8.4, 7.9 Hz, 1H, $CH_{Ar(2)}$), 4.83 (t, J = 6.5 Hz, 2H, CH_2), 3.45 (t, J = 6.5 Hz, 2H, CH_2), 3.18 (bs, 1H, O<u>H</u>O), 3.06 (s, 3H, CH_3), 2.64 (s, 3H, CH_3), 1.90 (s, 6H, CH_3).

^{13}C NMR (101 MHz, Chloroform-*d*) δ 158.0, 155.4, 148.1, 137.0, 136.0, 129.7, 129.30, 128.8, 126.4, 124.2, 123.4, 48.7, 31.1, 27.1, 22.3 (2x).[1]

HRMS (ESI+, *m/z*): calcd for $C_{15}H_{15}BrN^+$ [M^+]: 288.0382, found: 288.0381.

10-Bromo-2,4-dimethyl-6,7-dihydropyrido[2,1-a]isoquinolin-5-ium diacetate (**8g⁺**)

[1] Carbonyl carbon of acetate counterion (~178 ppm) could not be visualized for this compound on ^{13}C NMR.

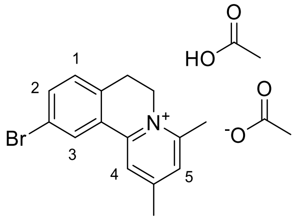

8g⁺

Reaction was performed starting from 7-bromo-**THIQ** (450 mg, 2.12 mmol) and acetone (3.1 mL, 42.44 mmol). Product (360 mg, 0.884 mmol) was obtained as a brown oil in 42% yield.

^{1}H NMR (400 MHz, Chloroform-*d*) δ 7.99 (d, J = 1.9 Hz, 1H, $CH_{Ar(3)}$), 7.86 (s, 1H, $CH_{Ar(4)}$), 7.66 (dd, J = 8.1, 1.9 Hz, 1H, $CH_{Ar(2)}$), 7.56 (s, 1H, $CH_{Ar(5)}$), 7.29 (d, J = 8.1 Hz, 1H, $CH_{Ar(1)}$), 4.79 (t, J = 6.5 Hz, 2H, CH_2), 3.30 (t, J = 6.5 Hz, 2H, CH_2), 3.01 (s, 3H, CH_3), 2.64 (s, 3H, CH_3), 1.90 (s, 6H, CH_3).

^{13}C NMR (101 MHz, Chloroform-*d*) δ 176.0, 158.4, 155.8, 147.8, 136.4, 135.2, 130.4, 129.9, 129.6, 128.9, 123.4, 122.4, 49.3, 26.7, 23.0, 22.5 (2x).

HRMS (ESI+, *m/z*): calcd for $C_{15}H_{15}BrN^+$ [M^+]: 288.0382, found: 288.0380.

10-Fluoro-2,4-dimethyl-6,7-dihydropyrido[2,1-a]isoquinolin-5-ium diacetate (**8h⁺**)

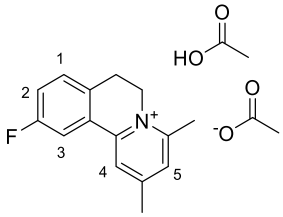

8h⁺

Reaction was performed starting from 7-fluoro-**THIQ** (0.5 mmol) and acetone (10.0 mmol). Product (0.065 g, 0.187 mmol) was obtained as a black solid in 37% yield.

^{1}H NMR (400 MHz, Chloroform-*d*) δ 7.87 (s, 1H, $CH_{Ar(4)}$), 7.60 (dd, J = 9.2, 2.5 Hz, 1H, $CH_{Ar(1)}$), 7.56 (s, 1H, $CH_{Ar(5)}$), 7.38 (dd, J = 8.4, 5.3 Hz, 1H, $CH_{Ar(3)}$), 7.33 – 7.18 (m, 2H, $CH_{Ar(2)}$, O<u>H</u>O), 4.81 (t, J = 6.6 Hz, 2H, CH_2), 3.31 (t, J = 6.6 Hz, 2H, CH_2), 3.03 (s, 3H, CH_3), 2.63 (s, 3H, CH_3), 1.89 (s, 6H, CH_3).

^{19}F NMR (376 MHz, Chloroform-*d*) δ -112.01.

^{13}C NMR (101 MHz, Chloroform-*d*) δ 176.2, 162.4 (d, J = 247.6 Hz), 158.0, 155.6, 147.9 (d, J = 3.0 Hz), 131.9 (d, J = 3.0 Hz), 130.4 (d, J = 8.1 Hz), 129.3, 128.2 (d, J = 8.1 Hz), 123.1, 120.5 (d, J = 21.6 Hz), 113.7 (d, J = 24.6 Hz), 49.3, 26.2, 23.3, 22.2, 22.1.

HRMS (ESI+, *m/z*): calcd for $C_{15}H_{15}FN^+$ [M^+]: 228.1183, found: 228.1180.

2,4-Dimethyl-10-(trifluoromethyl)-6,7-dihydropyrido[2,1-a]isoquinolin-5-ium diacetate (**8i**$^+$)

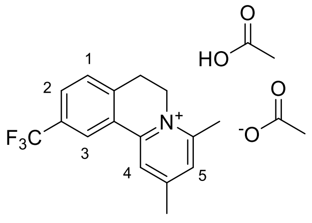

8i$^+$

Reaction was performed starting from 7-trifluoromethyl-**THIQ** (96 mg, 0.477 mmol) and acetone (0.70 mL, 9.54 mmol). Product (74 mg, 0.187 mmol) was obtained as a black solid in 39% yield.

^{1}H NMR (400 MHz, Chloroform-*d*) δ 8.09 (s, 1H, $CH_{Ar(3)}$), 7.94 (s, 1H, $CH_{Ar(4)}$), 7.80 (d, *J* = 7.9 Hz, 1H, $CH_{Ar(2)}$), 7.62 – 7.54 (m, 2H, $CH_{Ar(1+5)}$), 6.48 (bs, 1H, O<u>H</u>O), 4.88 (t, *J* = 6.2 Hz, 2H, CH_2), 3.46 (t, *J* = 6.2 Hz 2H, CH_2), 3.06 (s, 3H, CH_3), 2.66 (s, 3H, CH_3), 1.88 (s, 6H, CH_3).

^{13}C NMR (101 MHz, Chloroform-*d*) δ 175.9, 158.3, 155.6, 147.5, 140.0, 131.2 (q, *J* = 34 Hz), 129.6 (q, *J* = 3.5 Hz), 129.5, 129.4, 127.5, 123.8 (q, *J* = 4.8 Hz), 123.5 (q, *J* = 271.3 Hz), 123.2, 48.7, 26.8, 22.8, 22.1, 22.0.

^{19}F NMR (376 MHz, Chloroform-*d*) δ -62.72.

HRMS (ESI+, *m/z*): calcd for $C_{16}H_{15}F_3N^+$ [M^+]: 278.1151, found: 278.1152.

2,4-Dimethyl-10-nitro-6,7-dihydropyrido[2,1-a]isoquinolin-5-ium diacetate (**8j**$^+$)

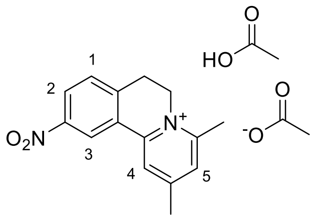

8j$^+$

Reaction was performed starting from 7-nitro-**THIQ** (89 mg, 0.5 mmol) and acetone (0.74 mL, 10.0 mmol). Product (76 mg, 0.204 mmol) was obtained as a black solid in 41% yield.

^{1}H NMR (400 MHz, Chloroform-*d*) δ 8.74 (s, 1H, $CH_{Ar(3)}$), 8.34 (d, *J* =10.6 Hz, 1H, $CH_{Ar(2)}$), 8.09 (s, 1H, $CH_{Ar(4)}$), 7.64-7.59 (m, 2H, $CH_{Ar(1,5)}$), 4.82 (t, *J* = 6.1 Hz, 2H, CH_2), 3.47 (t, *J* = 6.1 Hz, 2H, CH_2), 2.98 (s, 3H, CH_3), 2.66 (s, 3H, CH_3), 1.91 (s, 6H, CH_3).

^{13}C NMR (101 MHz, Chloroform-*d*) δ 175.04, 158.8, 155.7, 148.0, 146.8, 142.9, 130.0, 129.9, 128.1, 127.3, 123.7, 122.1, 53.6, 48.5, 26.9, 22.2, 21.9.

HRMS (ESI+, *m/z*): calcd for $C_{15}H_{15}N_2O_2^+$ [M$^+$]: 255.1128, found: 255.1126.

2-Ethyl-3,4-dimethyl-6,7-dihydropyrido[2,1-a]isoquinolin-5-ium diacetate (α) + 1,2,3,4-tetramethyl-6,7-dihydropyrido[2,1-a]isoquinolin-5-ium diacetate (β) (**8k$^+$**)

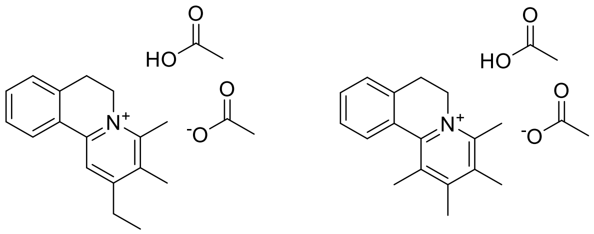

Major (α) **8k$^+$** Minor (β)

Reaction was performed starting from **THIQ** (266 mg, 2.0 mmol) and methyl ethyl ketone (3.6 mL, 40.0 mmol). Product (330 mg, 0.926 mmol) was obtained as a brown oil in 47% yield.

^{1}H NMR (400 MHz, Chloroform-*d*, α/β; 2:1) δ 7.82 – 7.73 (m, 2H$_{α+β}$, CH$_{Ar}$), 7.51 – 7.21 (m, 9H$_{α+β}$, CH$_{Ar}$ + O$\underline{H}$O), 4.63 (t, *J* = 6.6 Hz, 2H$_α$, CH$_2$), 4.46 (t, *J* = 6.1 Hz, 2H$_β$, CH$_2$), 3.14 (t, *J* = 6.6 Hz, 2H$_α$, CH$_2$), 3.02 (t, *J* = 6.1 Hz, 2H$_β$, CH$_2$), 2.82 – 2.70 (m, 8H$_{α+β}$, C$\underline{H}_2$CH$_3$ + CH$_3$ + CH$_3$), 2.46 (s, 3H$_β$, CH$_3$), 2.39 (s, 3H$_β$, CH$_3$), 2.35 – 2.29 (m, 6H$_{α+β}$, CH$_3$ + CH$_3$), 1.73 (s, 12H$_{α+β}$, CH$_3$), 1.20 (t, *J* = 7.6 Hz, 3H$_α$, CH$_2$C$\underline{H}_3$).

^{13}C NMR (151 MHz, Chloroform-*d*, α) δ 175.9 (α+β), 160.8, 153.4, 146.5, 135.1, 134.3, 132.7, 131.9, 128.4, 127.9, 126.4, 120.9, 49.4, 27.6, 26.6, 23.1 (α+β), 18.1, 15.5, 12.8.
^{13}C NMR (151 MHz, Chloroform-*d*, β) δ 175.9, 156.1, 150.9, 146.1, 137.3, 133.9, 131.7, 130.1, 127.2, 126.9, 126.7, 126.6, 50.9, 27.5, 23.1, 19.8, 18.5, 18.3, 16.7.

HRMS (ESI+, *m/z*): calcd for $C_{17}H_{20}N^+$ [M$^+$]: 238.1588, found: 238.1590.

3-Methoxy-2-(methoxymethyl)-4-methyl-6,7-dihydropyrido[2,1-a]isoquinolin-5-ium hydroxide (**8l**$^+$)

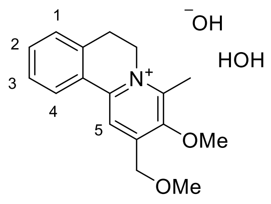

8l$^+$

Reaction was performed starting from **THIQ** (0.5 mmol) and methoxyacetone (10.0 mmol). After thorough drying of the crude, the diacetate counterion disappeared. From the NMR spectra it is presumed that a hydroxide replaced the diacetate. For calculating the yield, it was assumed that one hydroxide and one water molecule act as the counterion. Product (0.054 g, 0.178 mmol) was obtained as a brown oil in 35% yield.

^{1}H NMR (400 MHz, DMSO-*d6*) δ 8.49 (s, 1H, $CH_{Ar(5)}$), 8.14 (d, *J* = 8.0 Hz, 1H, $CH_{Ar(4)}$), 7.62 (dd, *J* = 8.0, 7.3 Hz, 1H, $CH_{Ar(3)}$), 7.59 – 7.49 (m, 2H, $CH_{Ar(1+2)}$), 4.61 (t, *J* = 6.5 Hz, 2H, CH_2), 3.86 (bs, 3H, CH_3), 3.47 (s, 2H, $C\underline{H}_2OCH_3$), 3.33 (bs, 3H, $O\underline{H}$), 3.26 – 3.20 (m, 2H, CH_2), 2.76 (s, 3H, $OC\underline{H}_3$), 2.58 (s, 3H, $CH_2OC\underline{H}_3$).

^{13}C NMR (151 MHz, DMSO-*d6*) δ 153.81, 150.37, 149.92, 144.08, 135.31, 132.45, 128.16, 128.07, 126.90, 126.67, 123.92, 61.40, 49.56, 49.13, 25.78, 16.58, 14.29.

HRMS (ESI+, *m/z*): calcd for $C_{17}H_{20}NO_2^+$ [M$^+$]: 270.1489, found: 270.1492.

7,9-Dimethyl-4,5-dihydrothieno[3,2-a]quinolizin-6-ium diacetate (**8m**$^+$)

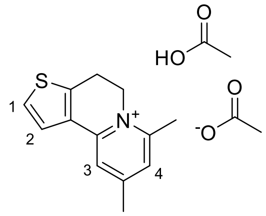

8m$^+$

Reaction was performed starting from 4,5,6,7-tetrahydrothieno[3,2-c]pyridine (2.0 mmol, CAS: 54903-50-3) and acetone (40.0 mmol). Product (0.307 g, 0.780 mmol) was obtained as a brown oil in 39% yield.

^{1}H NMR (400 MHz, Chloroform-*d*) δ 8.32 (bs, 1H, $O\underline{H}O$), 7.67 (s, 1H, $CH_{Ar(3)}$), 7.44 (d, *J* = 5.4 Hz, 1H, $CH_{Ar(2)}$), 7.38 (s, 1H, $CH_{Ar(4)}$), 7.34 (d, *J* = 5.4 Hz, 1H, $CH_{Ar(1)}$), 4.94 (t, *J* = 7.1 Hz, 2H, CH_2), 3.51 (t, *J* = 7.1 Hz, 2H, CH_2), 3.00 (s, 3H, CH_3), 2.57 (s, 3H, CH_3), 1.93 (s, 6H, CH_3).

^{13}C NMR (101 MHz, Chloroform-*d*) δ 176.0, 158.0, 154.0, 145.7, 143.6, 128.7, 127.6, 126.7, 124.0, 121.5, 49.8, 23.0 (2x), 22.4, 22.0.

HRMS (ESI+, *m/z*): calcd for $C_{13}H_{14}NS^+$ [M^+]: 216.0842, found: 216.0839.

2,4-Dimethyl-7,12-dihydro-6H-indolo[2,3-a]quinolizin-5-ium diacetate (**8n**$^+$)

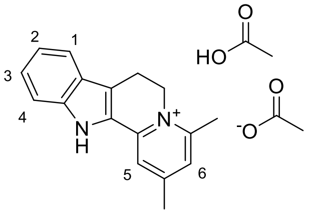

8n$^+$

Reaction was performed starting from Noreleagnine (2.0 mmol, CAS: 16502-01-5) and acetone (40.0 mmol). Product (0.278 g, 0.76 mmol) was obtained as a red solid in 38% yield.

^{1}H NMR (600 MHz, DMSO-*d6*) δ 8.26 (s, 1H, $CH_{Ar(5)}$), 7.70 (d, *J* = 8.0 Hz, 1H, $CH_{Ar(4)}$), 7.58 (s, 1H, $CH_{Ar(6)}$), 7.55 (d, *J* = 8.4 Hz, 1H, $CH_{Ar(1)}$), 7.32 (dd, *J* = 8.0 Hz, *J* = 7.6 Hz, 1H, $CH_{Ar(3)}$), 7.13 (dd, *J* = 8.4 Hz, *J* = 7.6 Hz, 1H, $CH_{Ar(2)}$), 4.68 (t, *J* = 7.4 Hz, 2H, CH_2), 3.32 (t, *J* = 7.4 Hz, 2H, CH_2), 2.79 (s, 3H, CH_3), 2.53 (s, 3H, CH_3), 1.82 (s, 6H, CH_3).

^{13}C NMR (151 MHz, DMSO-*d6*) δ 172.8, 156.6, 154.1, 142.8, 139.7, 126.1, 125.9, 125.7, 124.5, 120.4, 120.3, 119.6, 116.3, 112.9, 49.3, 22.6, 21.2, 21.1, 18.8.

HRMS (ESI+, *m/z*): calcd for $C_{17}H_{17}N_2^+$ [M^+]: 249.1386, found: 249.1385.

Synthesis and characterization of pyrrole product **12**

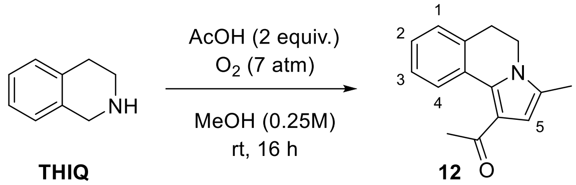

In a pressure vessel equipped with a Teflon coated stirring bar, **THIQ** (27 mg, 0.2 mmol, 1.0 equiv.), 2,5-hexanedione (1.9 mL, 16 mmol, 80 equiv.) and AcOH (24 mg, 0.4 mmol, 2 equiv.) were dissolved in anhydrous methanol (0.8 mL, 0.25M). The vessel was charged with 7 bars oxygen overpressure and was stirred overnight at room temperature. Methanol and AcOH were removed under vacuum, after which 2,5-hexanedione was removed by Kugelrohr distillation. The residue was purified by column chromatography (SiO_2, pentane/EtOAc, 9:1). Product (12.3 mg, 0.055 mmol) was obtained as a brown solid in 27% yield.

^{1}H NMR (400 MHz, Chloroform-*d*) δ 8.50 (d, *J* = 7.8 Hz, 1H, $CH_{Ar(4)}$), 7.32 – 7.27 (m, 1H, $CH\text{-}_{Ar(1)}$), 7.24 – 7.15 (m, 2H, $CH_{Ar(2,3)}$), 6.38 (s, 1H, $CH_{Ar(5)}$), 3.88 (t, *J* = 6.4 Hz, 2H, CH_2), 3.00 (d, *J* = 6.4 Hz, 2H, CH_2), 2.50 (s, 3H, CH_3), 2.27 (s, 3H, CH_3).

^{13}C NMR (101 MHz, Chloroform-*d*) δ 194.4, 132.3, 131.4, 128.4, 127.6, 127.4, 127.2, 127.0, 126.9, 120.3, 110.9, 40.7, 29.5, 29.3, 11.8.

HRMS (ESI+, *m/z*): calcd for $C_{15}H_{15}NOH^+$ $[M+H]^+$: 226.1225, found: 226.1226.

X-ray diffraction:
Deposition number 2359485 contains the supplementary crystallographic data for compound **12** in this paper.

These data are provided free of charge by The Cambridge Crystallographic Data Centre and Fachinformations-zentrum Karlsruhe Access Structures service at www.ccdc.cam.ac.uk/structures.

A single crystal of **12** was mounted on the cryoloop (273 K) of a Bruker-D8 Venture diffractometer. Data collection and reduction was done using the Bruker software suite APEX3. The final unit cell was obtained from the xyz centroids of 9971 reflections after integration. A multiscan absorption correction was applied, based on the intensities of symmetry-related reflections measured at different angular settings (*SADABS*). The structures were solved by direct methods using *SHELXT*[11] and refinement of the structure was performed using *SHLELXL*.[12] The hydrogen atoms were generated by geometrical considerations, constrained to idealized geometries and allowed to ride on their carrier atoms with an isotropic displacement parameter related to the equivalent displacement parameter of their carrier atoms. Crystal data and details on collection and refinement are presented in the following table.

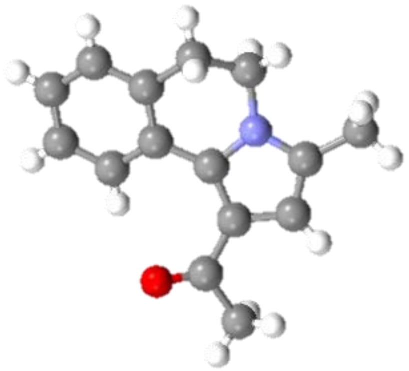

Chemical formula	$C_{15}H_{15}NO$
M_r	225.28
cryst syst	Orthorhombic
color, habit	orange,block
space group	$Pca2_1$
a (Å)	18.336(3)
b (Å)	8.4988(12)
c (Å)	7.7709(11)
α, °	90
β, °	90
γ, °	90

V (Å^3)	1211.0(3)
Z	4
ρ_{calc}, g•cm^{-3}	1.236
μ(Cu K$_\alpha$), cm^{-1}	0.606
F(000)	480
temp (K)	273(2)
ϑ range (°)	4.823 - 72.369
data collected (h,k,l)	-22:22, -10:10, -9:9
no. of rflns collected	28840
no. of indpndt reflns	2387
observed reflns ($F_o \geq 2$ $\sigma(F_o)$)	2280
R(F) (%)	3.75
wR(F^2) (%)	9.77
GooF	1.101
weighting a,b	0.0584, 0.0518
params refined	156
restraints	1
min, max resid dens	-0.248, 0.180
Flack x	0.06(12)

Table S5. Crystallographic data for pyrrole product **12**

NMR spectra

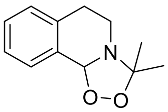

5

^{1}H NMR

400 MHz

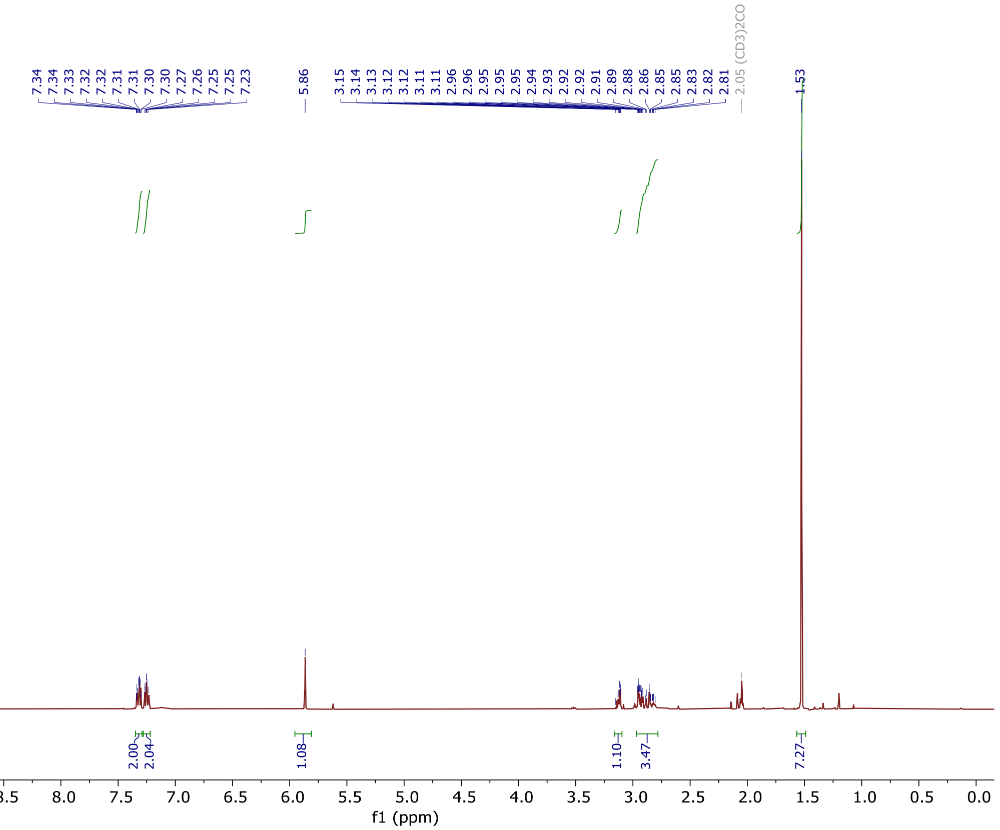

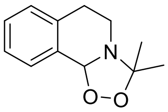

5

^{13}C NMR

101 MHz

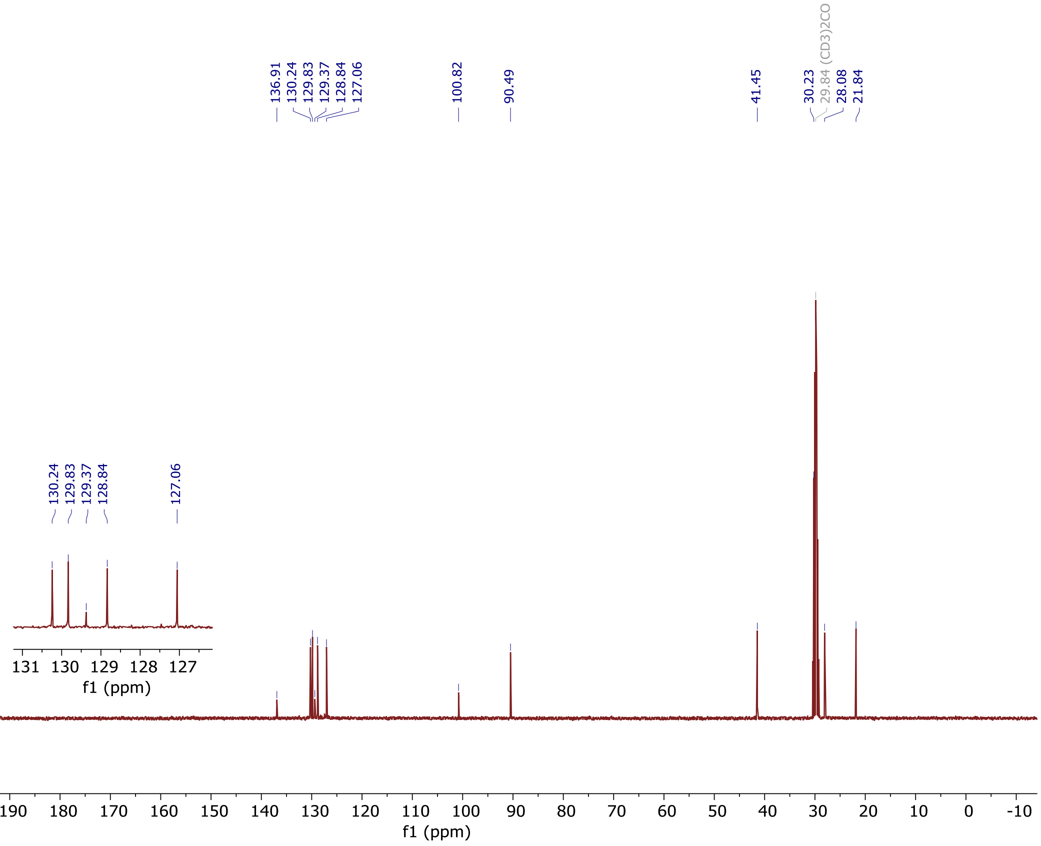

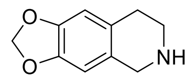

THIQe
^{1}H NMR
400 MHz

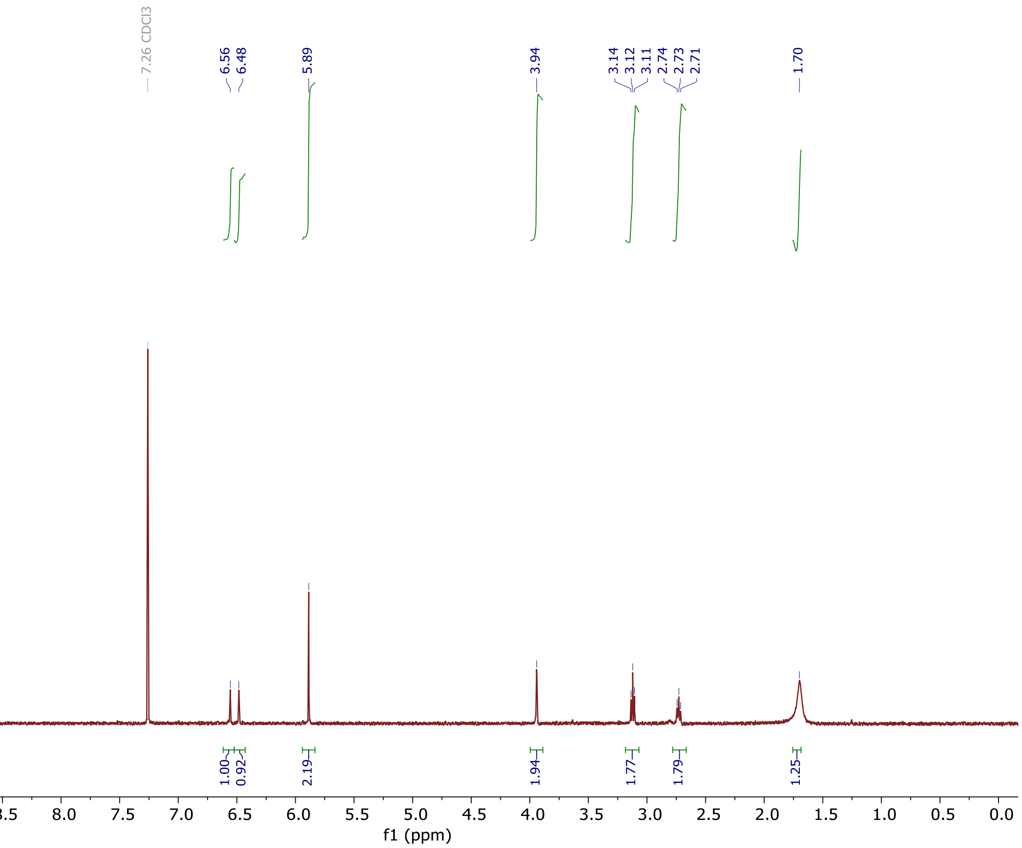

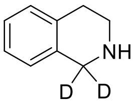

THIQ-*d2*
^{1}H NMR
600 MHz

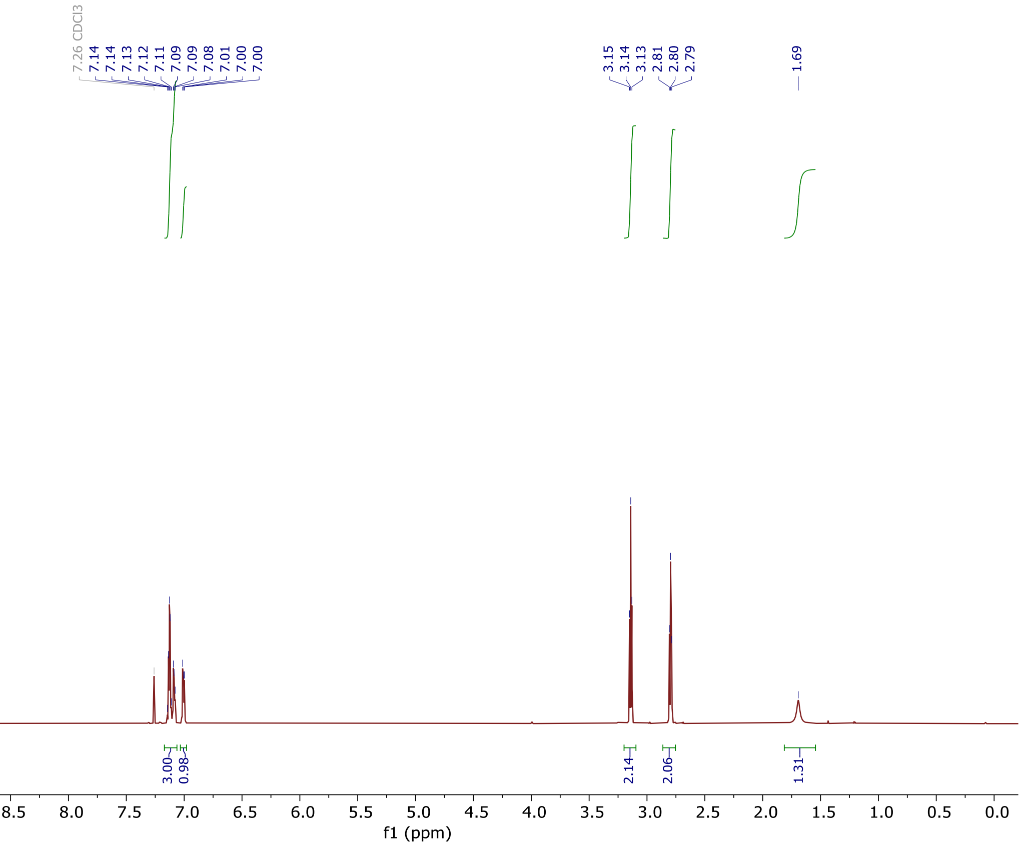

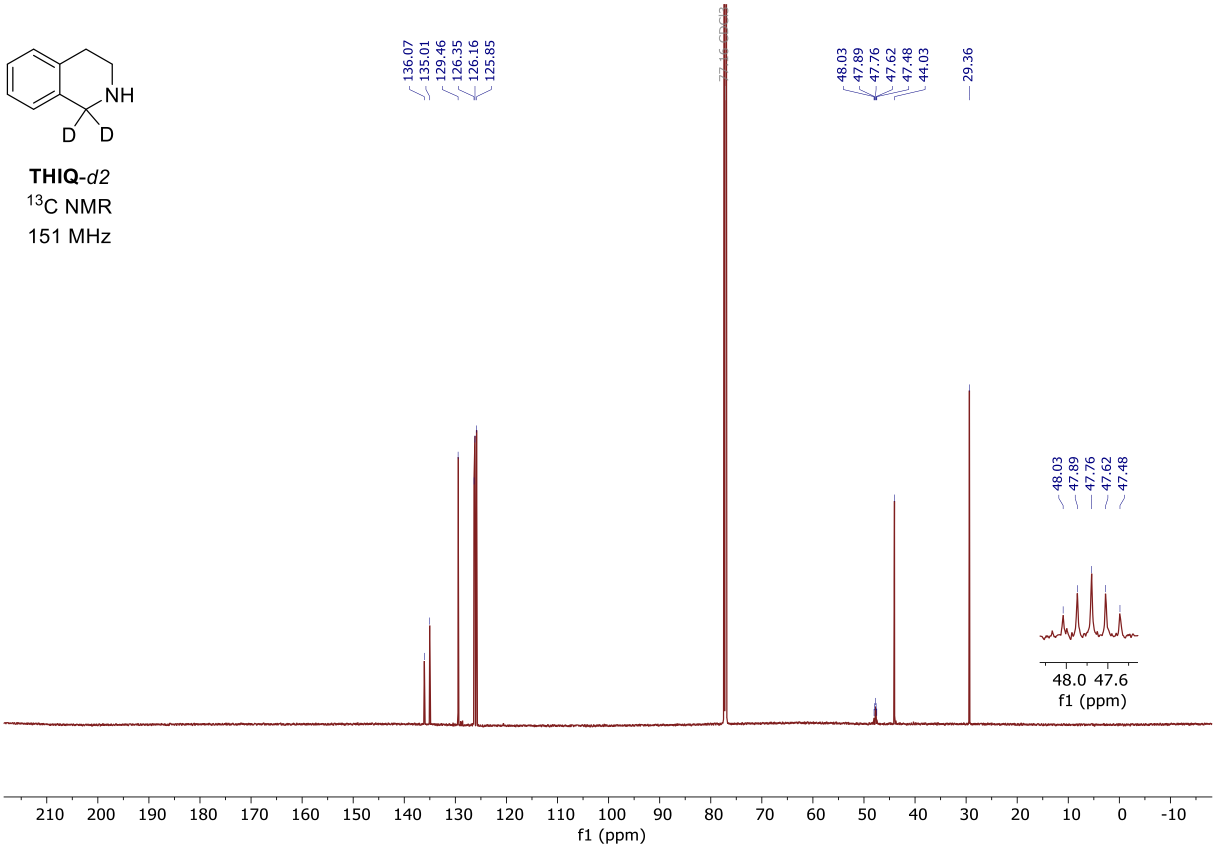
NH
D D
THIQ-$d2$
^{13}C NMR
151 MHz
136.07
135.01
129.46
126.35
126.16
125.85
77.16 CDCl3
48.03
47.89
47.76
47.62
47.48
44.03
29.36
48.0 47.6
f1 (ppm)
210 200 190 180 170 160 150 140 130 120 110 100 90 80 70 60 50 40 30 20 10 0 -10
f1 (ppm)

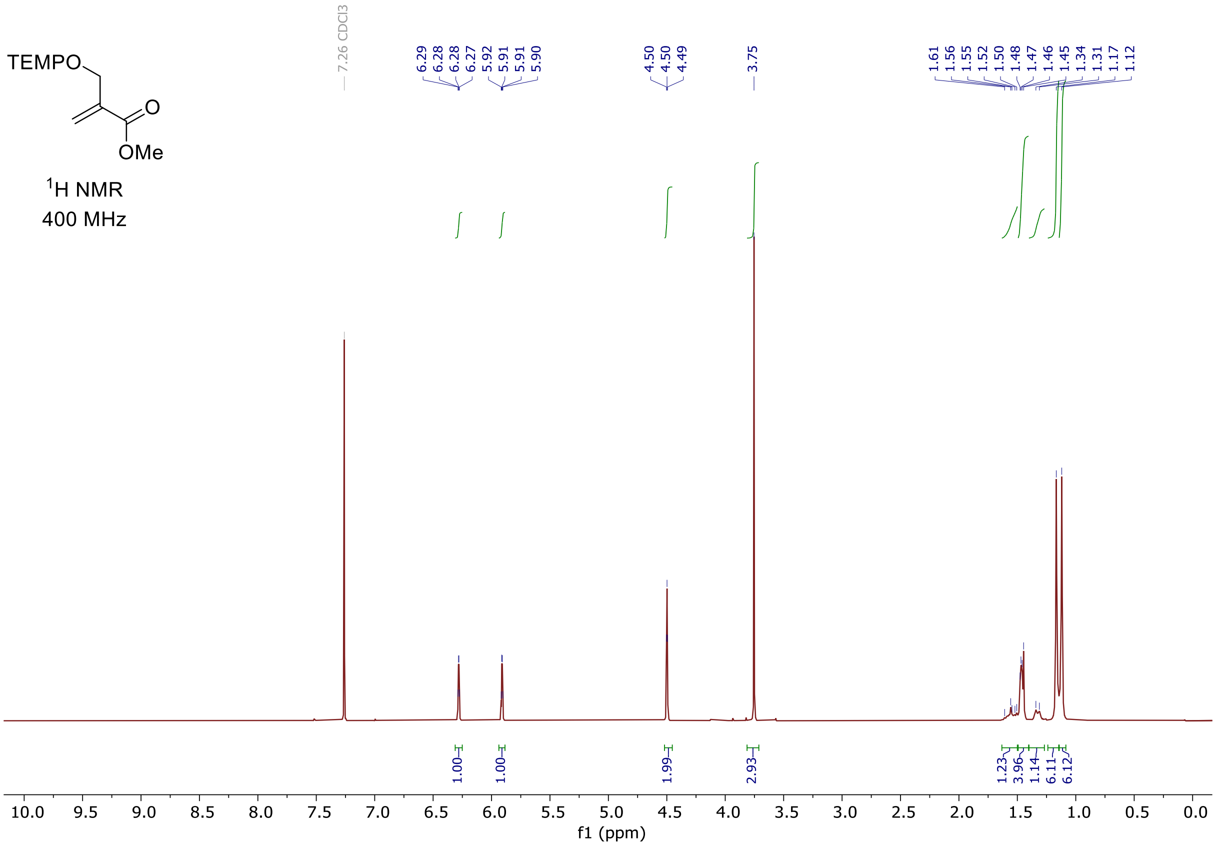
TEMPO
O
OMe
^{1}H NMR
400 MHz
7.26 CDCl3
6.29
6.28
6.28
6.27
5.92
5.91
5.91
5.90
4.50
4.50
4.49
3.75
1.61
1.56
1.55
1.52
1.50
1.48
1.47
1.46
1.45
1.34
1.31
1.17
1.12
1.00
1.00
1.99
2.93
1.23
3.96
1.14
6.11
6.12
10.0 9.5 9.0 8.5 8.0 7.5 7.0 6.5 6.0 5.5 5.0 4.5 4.0 3.5 3.0 2.5 2.0 1.5 1.0 0.5 0.0
f1 (ppm)

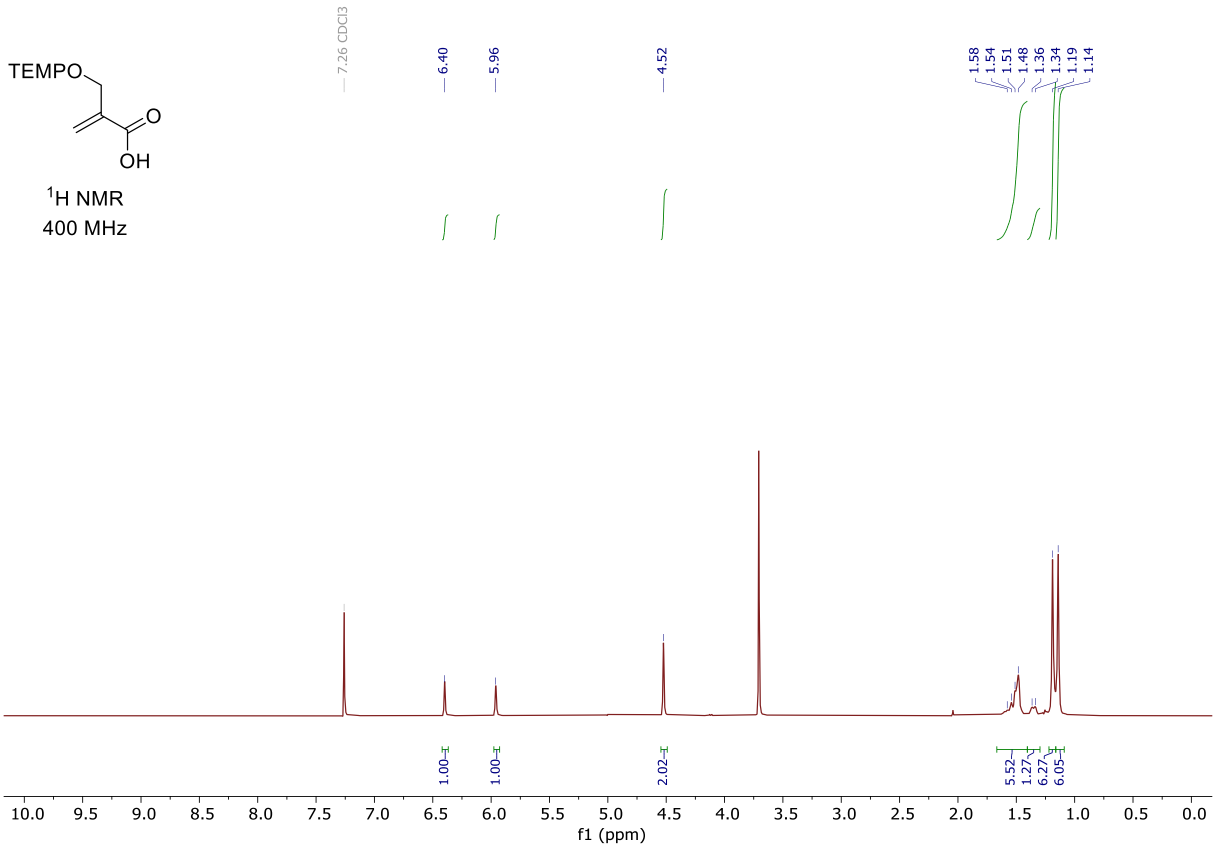
TEMPO
O
OH
^{1}H NMR
400 MHz
7.26 CDCl3
6.40
5.96
4.52
1.58
1.54
1.51
1.48
1.36
1.34
1.19
1.14
1.00
1.00
2.02
5.52
1.27
6.27
6.05
f1 (ppm)

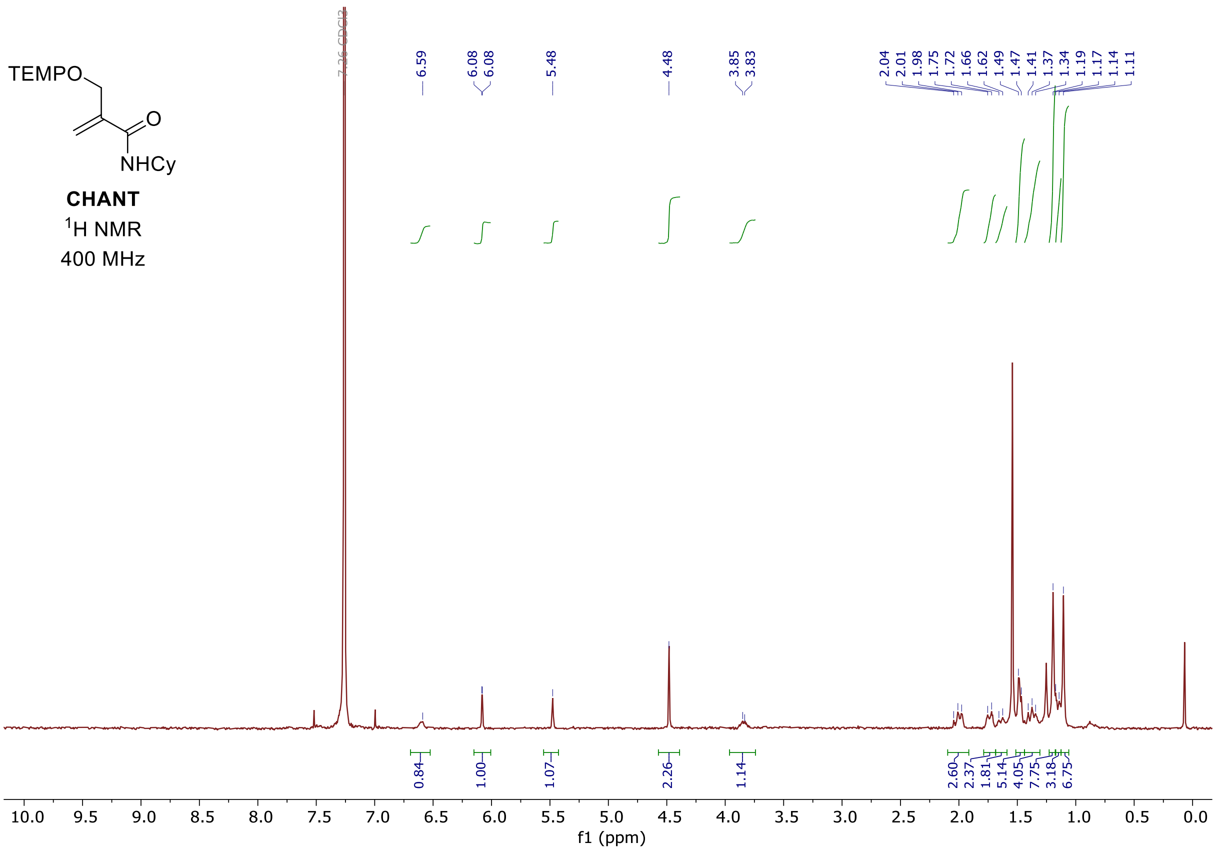
TEMPO
O
NHCy
CHANT
^{1}H NMR
400 MHz
6.59
6.08
6.08
5.48
4.48
3.85
3.83
2.04
2.01
1.98
1.75
1.72
1.66
1.62
1.49
1.47
1.41
1.37
1.34
1.19
1.17
1.14
1.11
0.84
1.00
1.07
2.26
1.14
2.60
2.37
1.81
5.14
4.05
7.75
3.18
6.75
f1 (ppm)

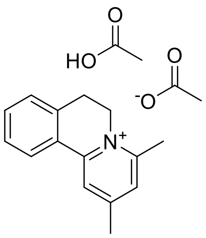

8a⁺

^{1}H NMR

400 MHz

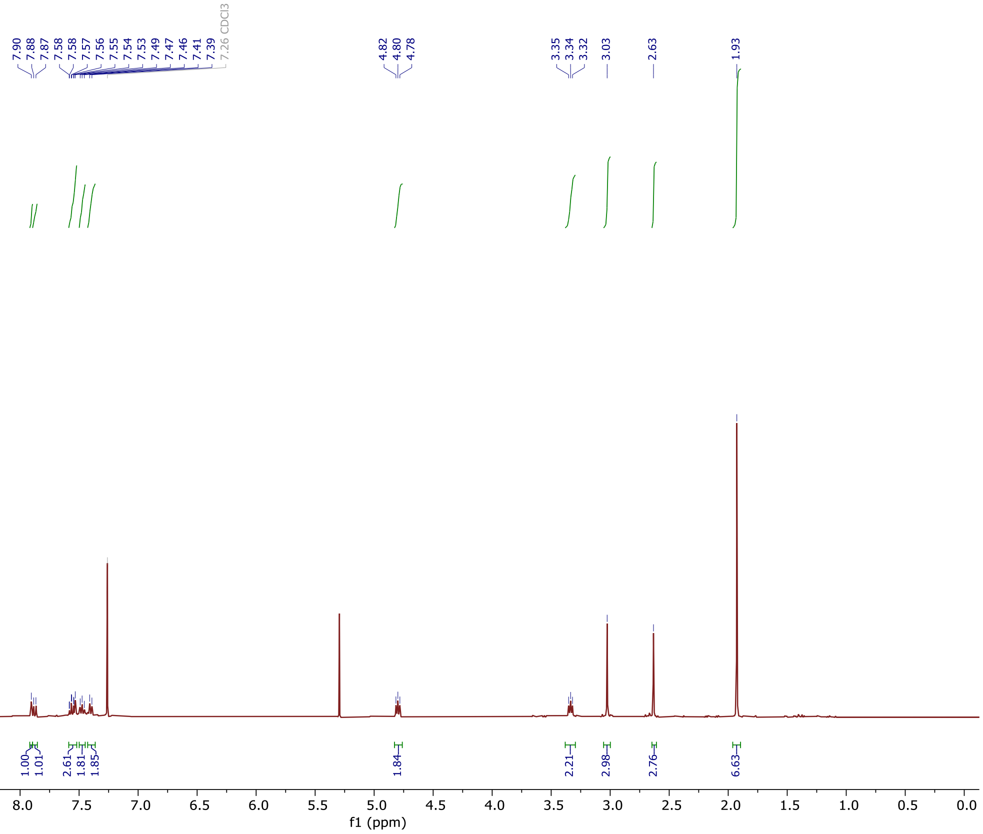

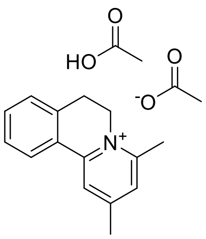

8a⁺

^{13}C NMR

101 MHz

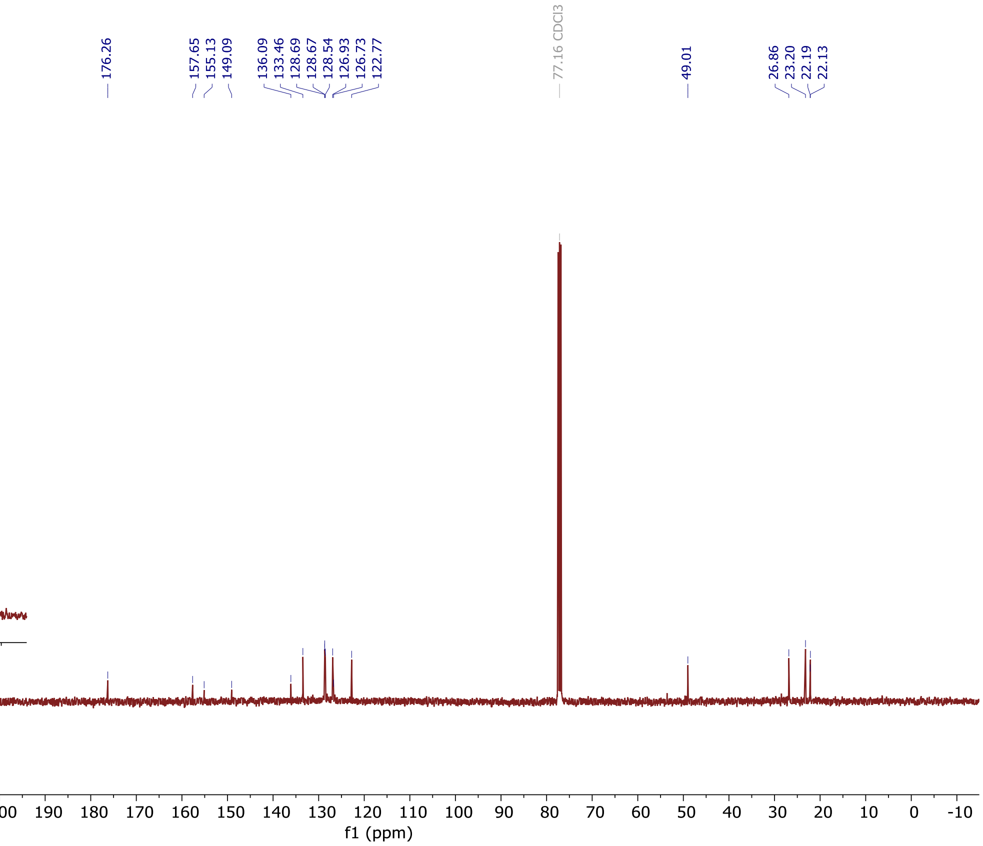

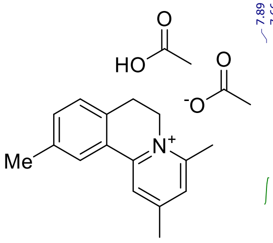

8b+
^{1}H NMR
400 MHz

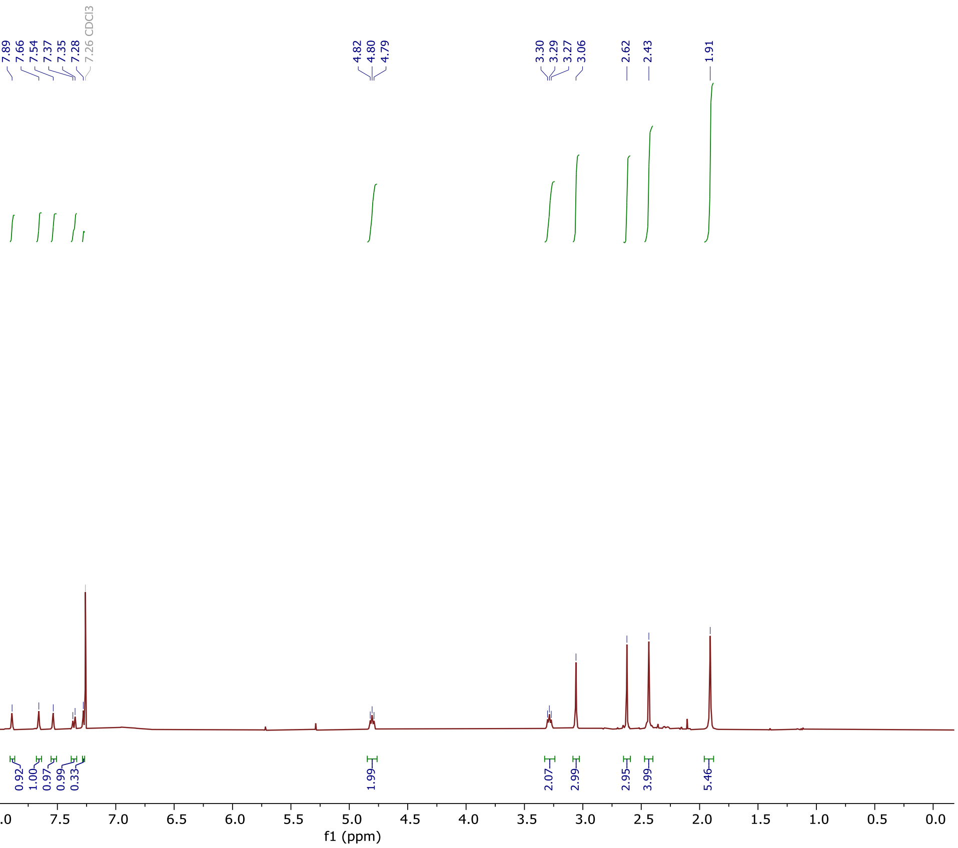

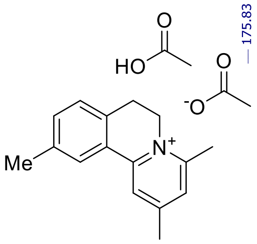

8b+
^{13}C NMR
101 MHz

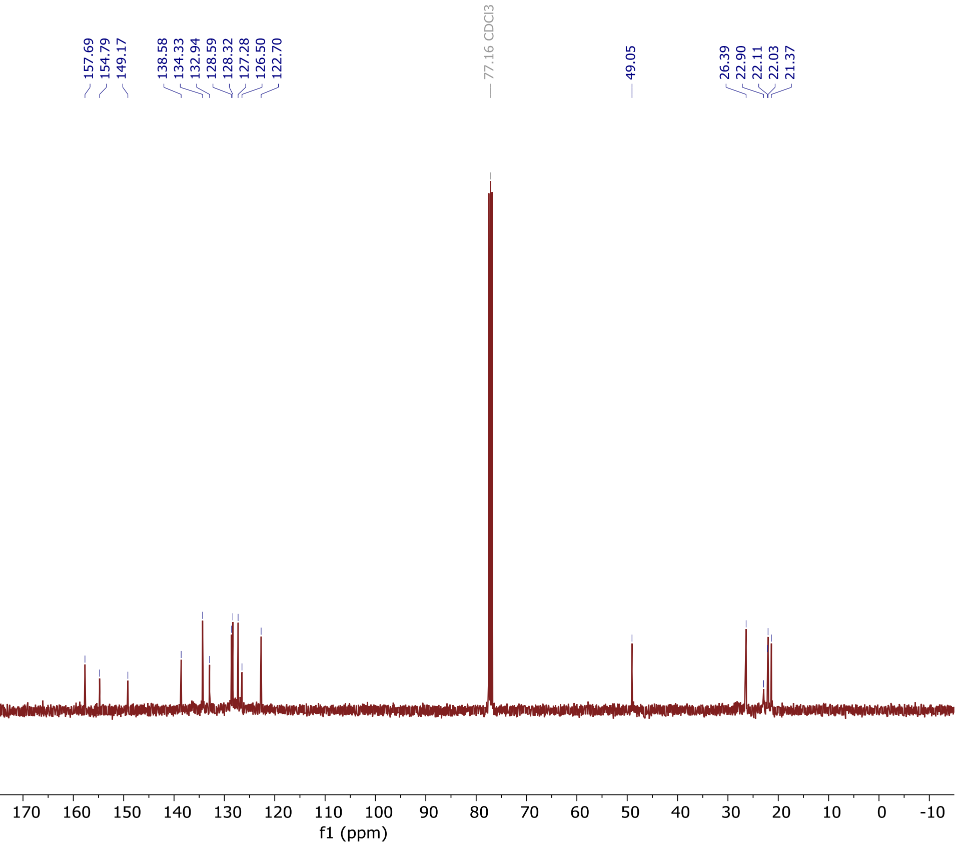

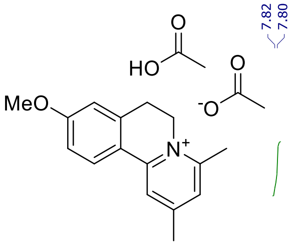

8c⁺

^{1}H NMR

400 MHz

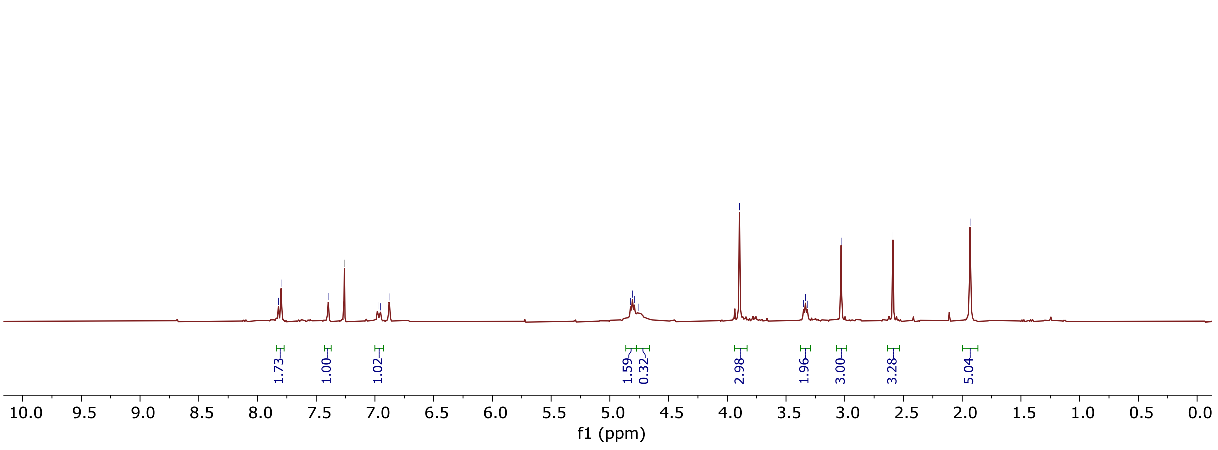

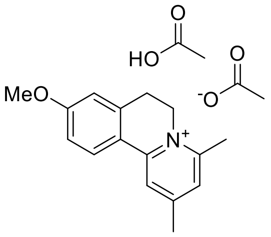

8c⁺

^{13}C NMR

151 MHz

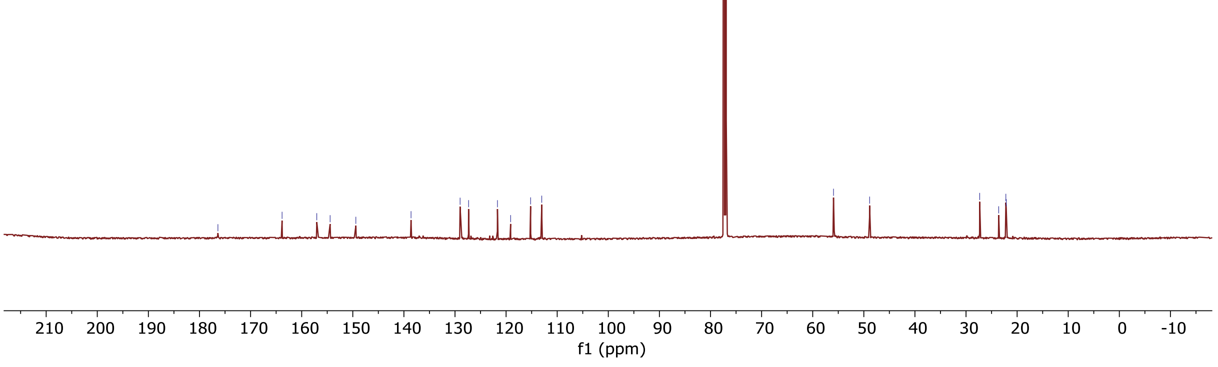

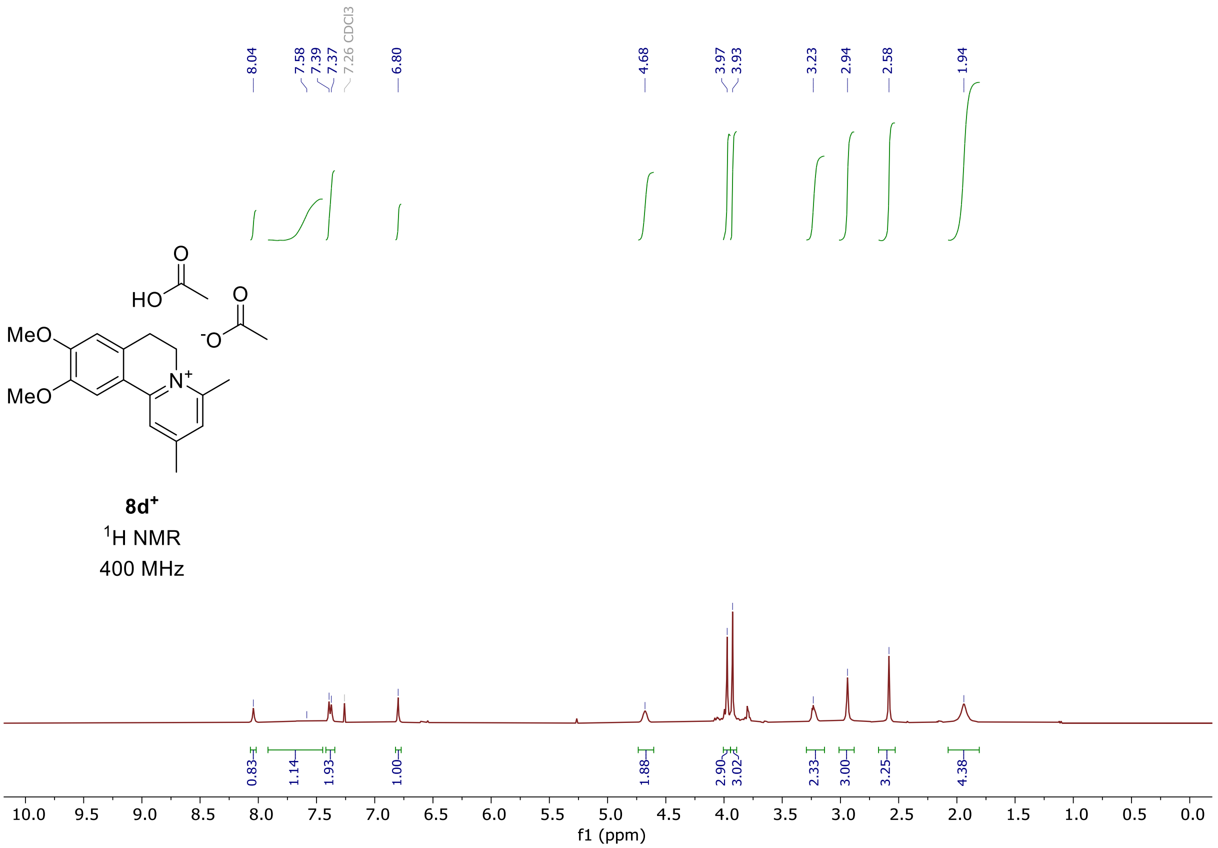
8d+
1H NMR
400 MHz
8.04
7.58
7.39
7.37
7.26 CDCl3
6.80
4.68
3.97
3.93
3.23
2.94
2.58
1.94
0.83
1.14
1.93
1.00
1.88
2.90
3.02
2.33
3.00
3.25
4.38
f1 (ppm)

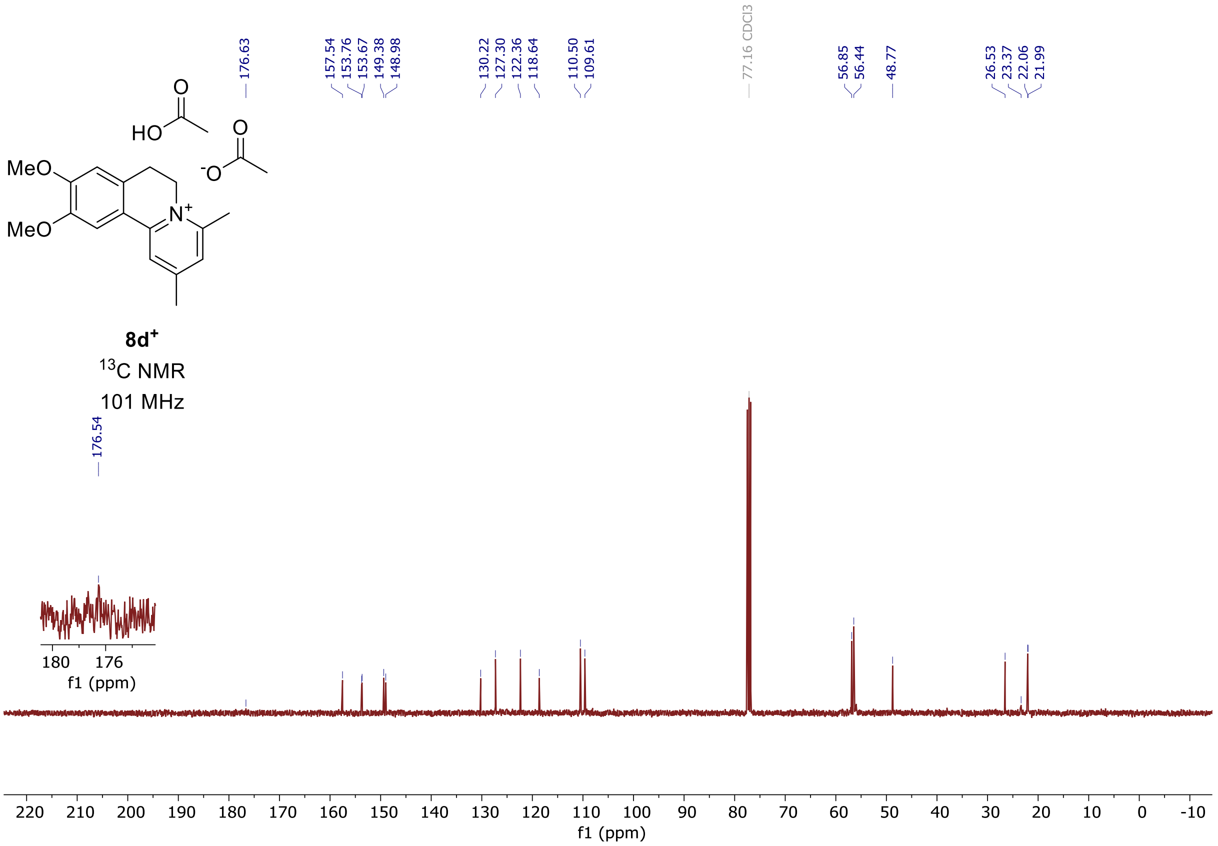
8d+
13C NMR
101 MHz
176.63
157.54
153.76
153.67
149.38
148.98
130.22
127.30
122.36
118.64
110.50
109.61
77.16 CDCl3
56.85
56.44
48.77
26.53
23.37
22.06
21.99
176.54
f1 (ppm)

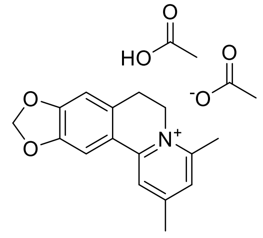

8e⁺

^{1}H NMR

400 MHz

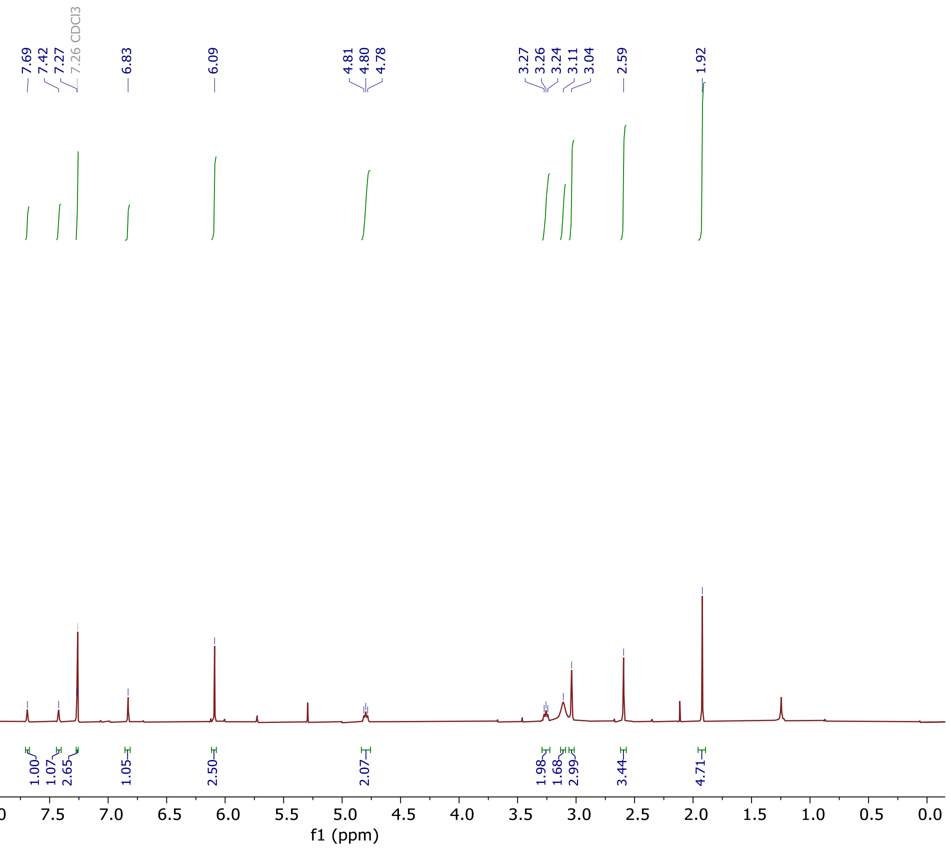

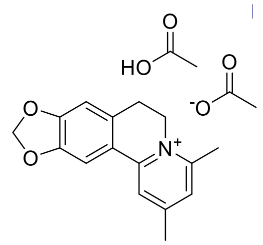

8e⁺

^{13}C NMR

101 MHz

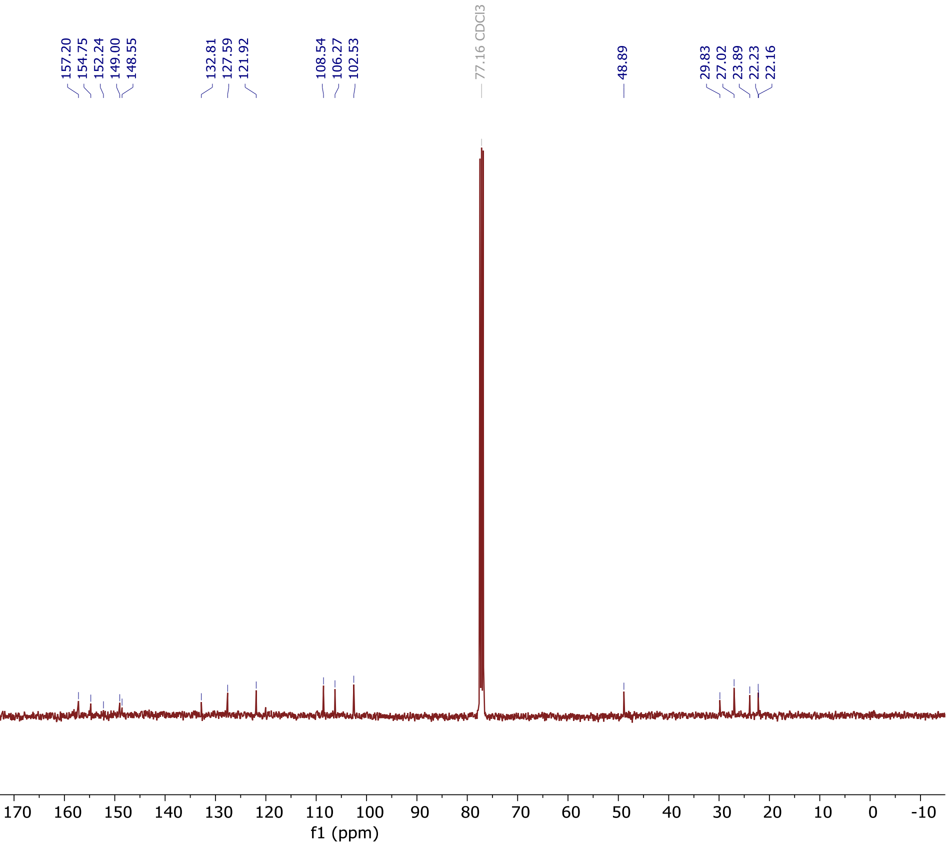

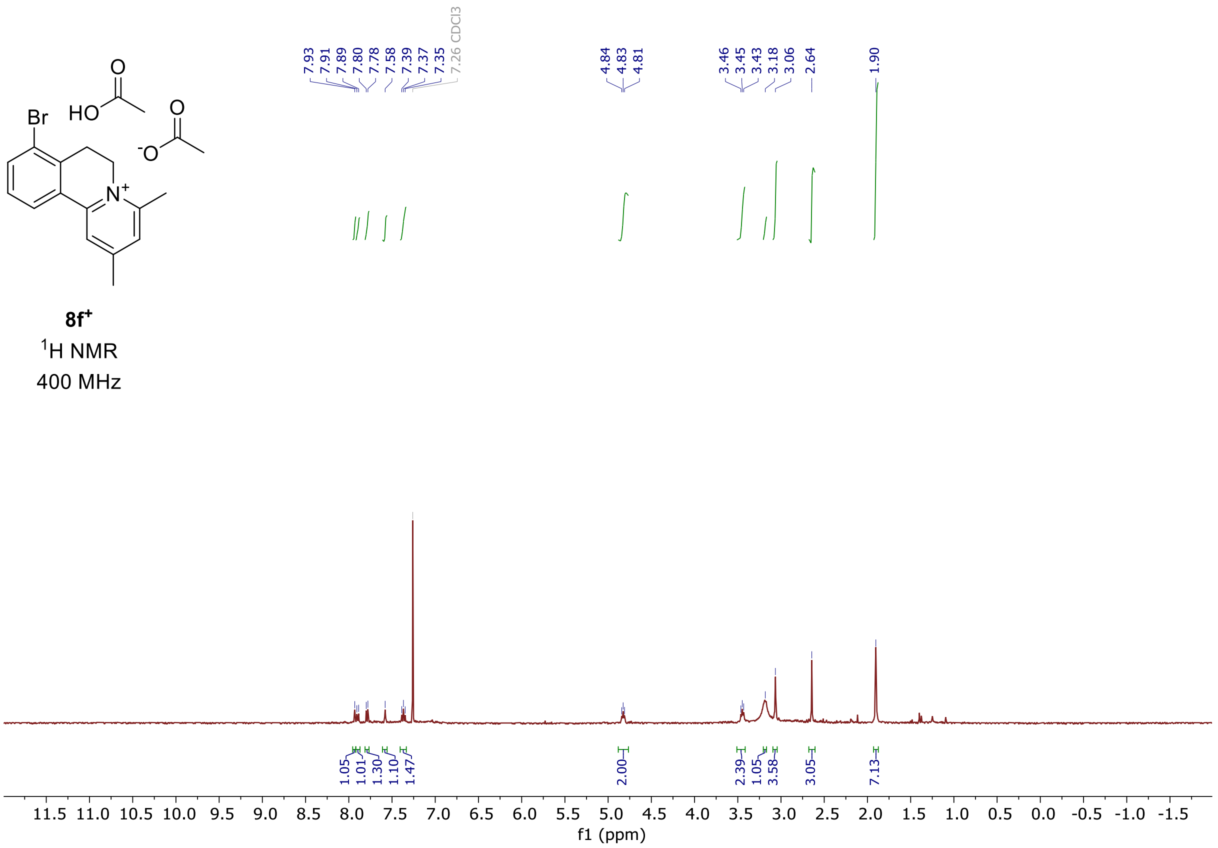

Br
HO
O
O
-O
N+
8f+
1H NMR
400 MHz
7.93
7.91
7.89
7.80
7.78
7.58
7.39
7.37
7.35
7.26 CDCl3
4.84
4.83
4.81
3.46
3.45
3.43
3.18
3.06
2.64
1.90
1.05
1.01
1.30
1.10
1.47
2.00
2.39
1.05
3.58
3.05
7.13
11.5 11.0 10.5 10.0 9.5 9.0 8.5 8.0 7.5 7.0 6.5 6.0 5.5 5.0 4.5 4.0 3.5 3.0 2.5 2.0 1.5 1.0 0.5 0.0 -0.5 -1.0 -1.5
f1 (ppm)

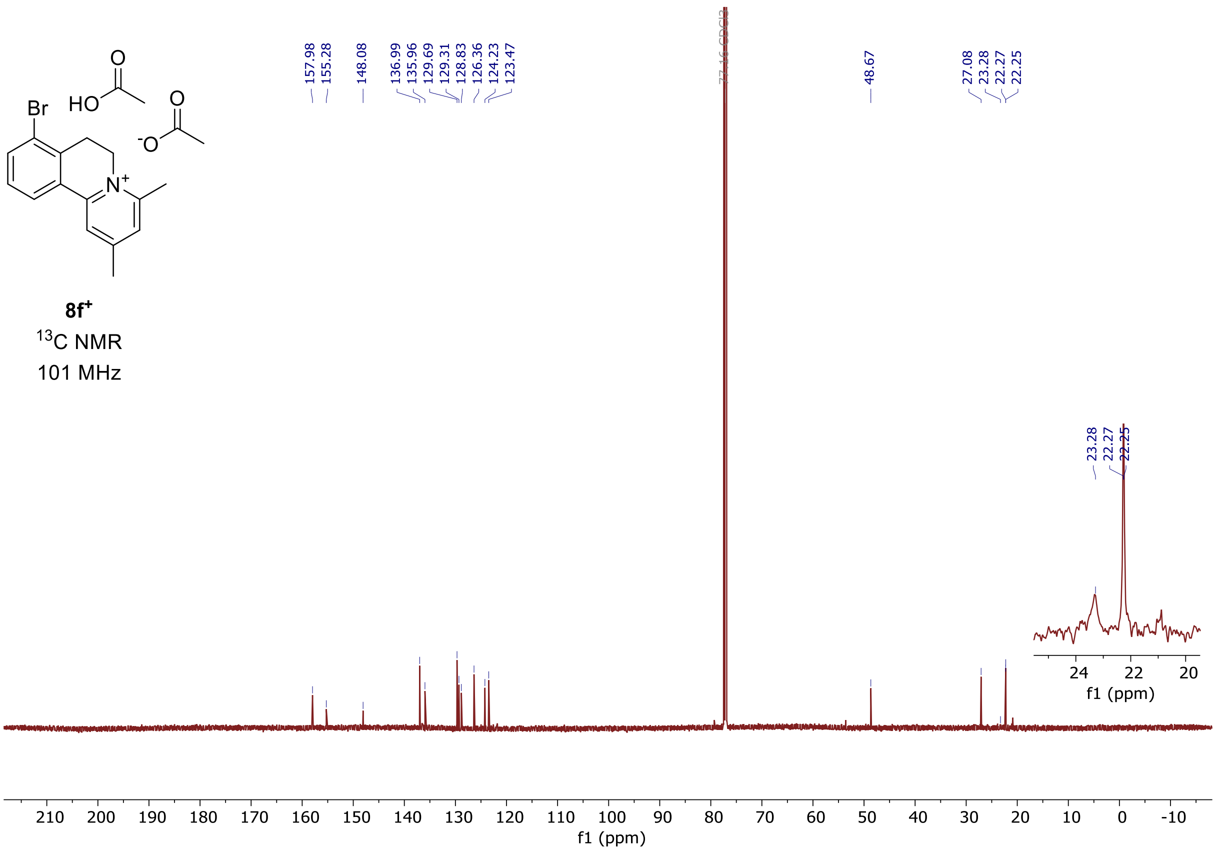

Br
HO
O
O
-O
N+
8f+
13C NMR
101 MHz
157.98
155.28
148.08
136.99
135.96
129.69
129.31
128.83
126.36
124.23
123.47
48.67
27.08
23.28
22.27
22.25
23.28
22.27
22.25
24 22 20
f1 (ppm)
210 200 190 180 170 160 150 140 130 120 110 100 90 80 70 60 50 40 30 20 10 0 -10
f1 (ppm)

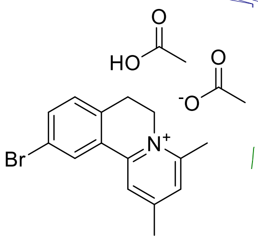

8g+
^{1}H NMR
400 MHz

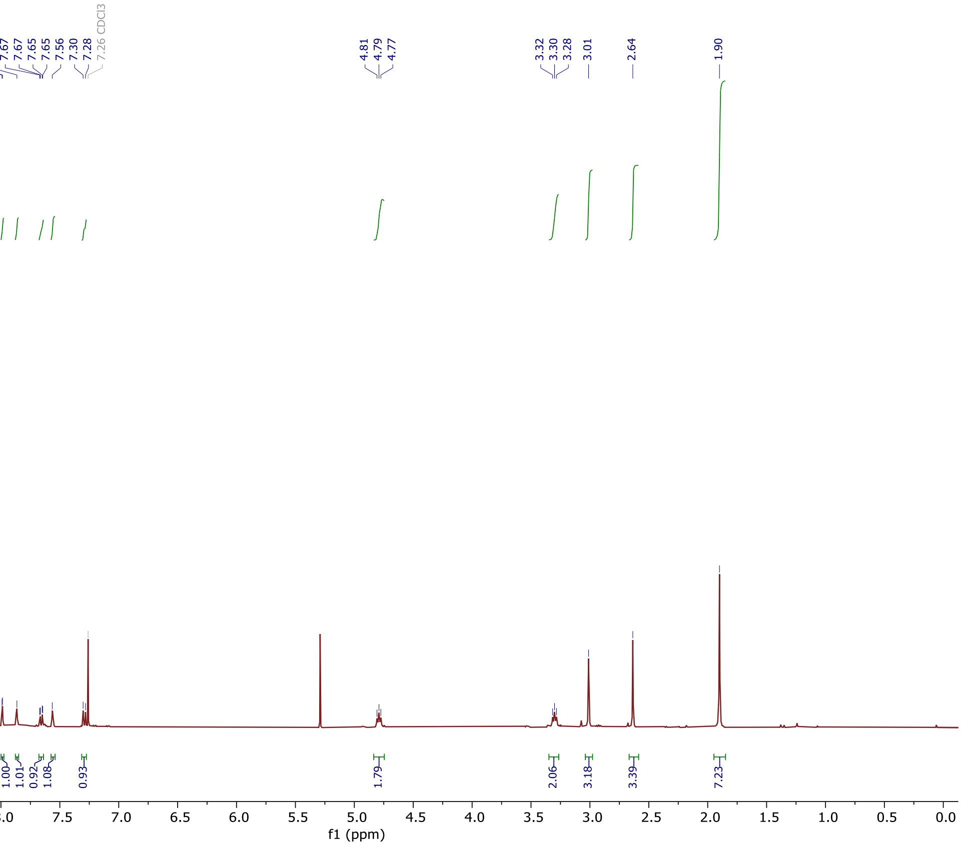

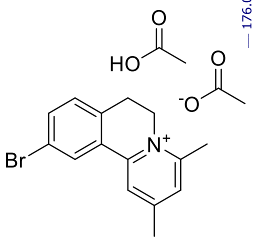

8g+
^{13}C NMR
101 MHz

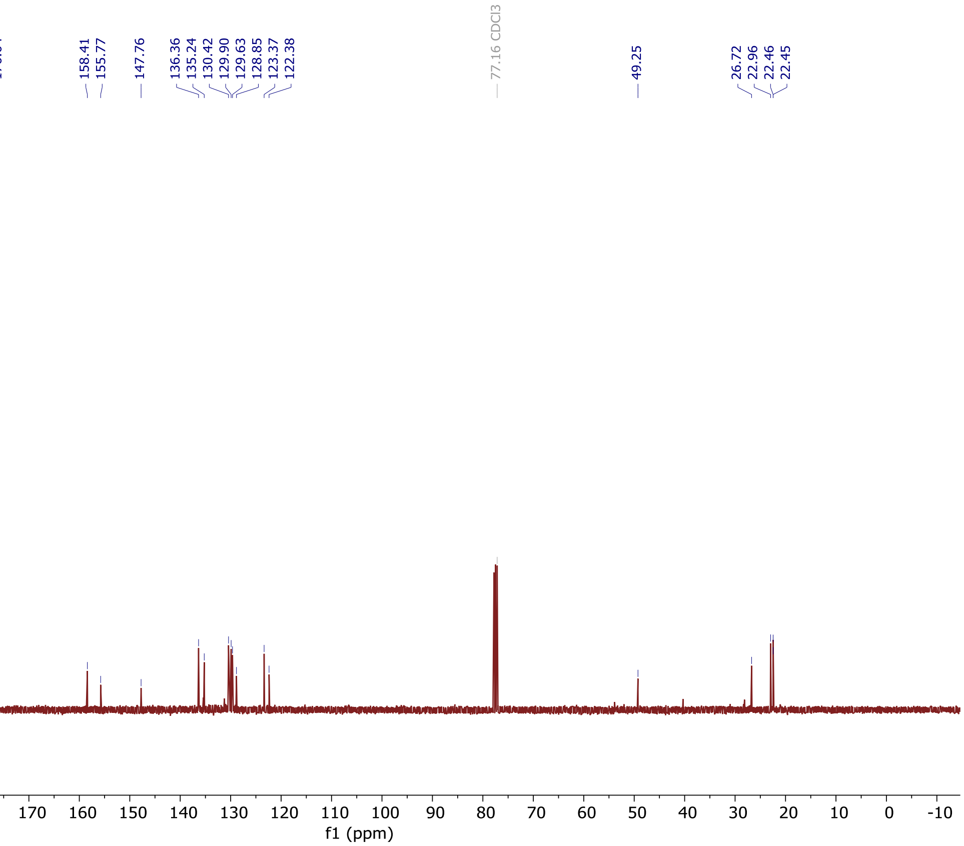

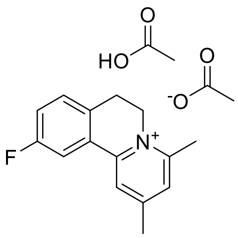

8h⁺

^{1}H NMR

400 MHz

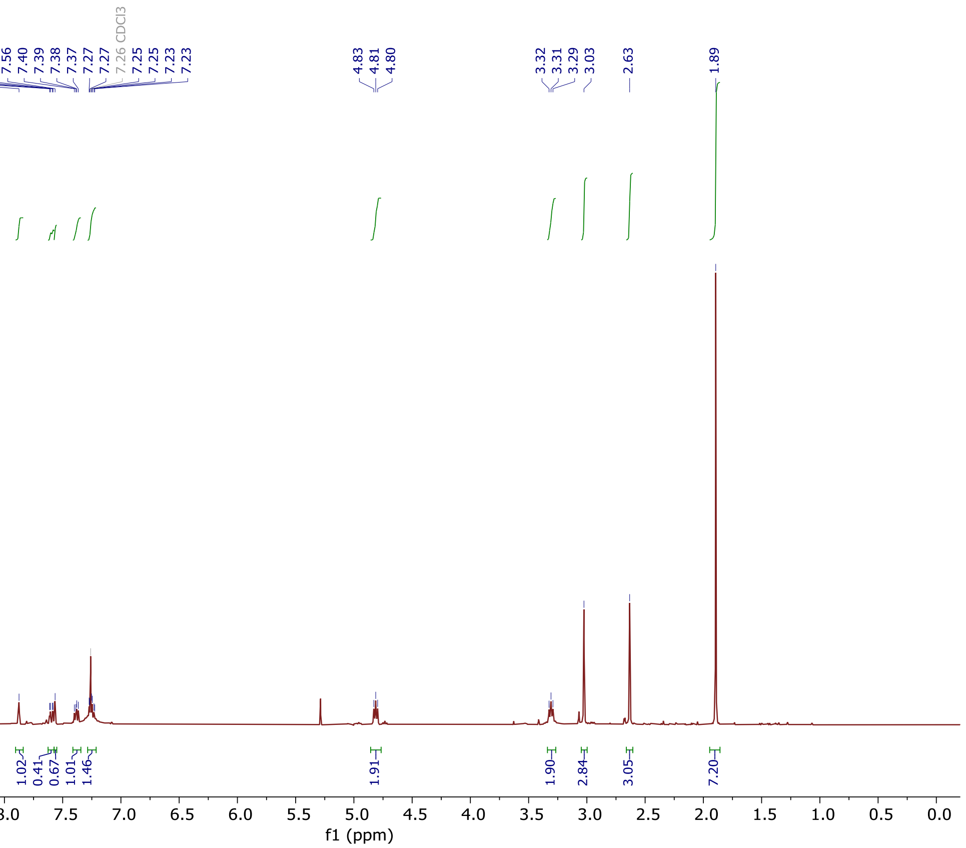

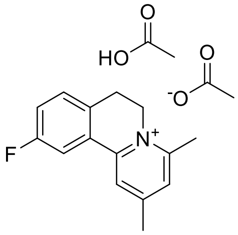

8h⁺

^{19}F NMR

376 MHz

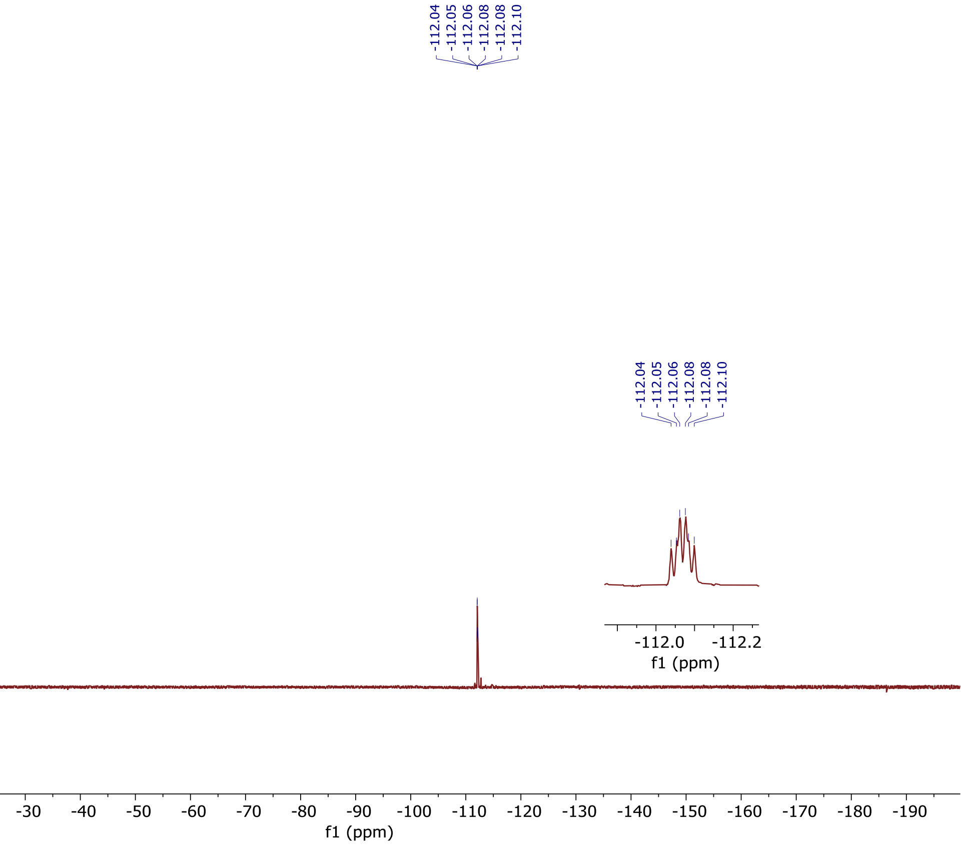

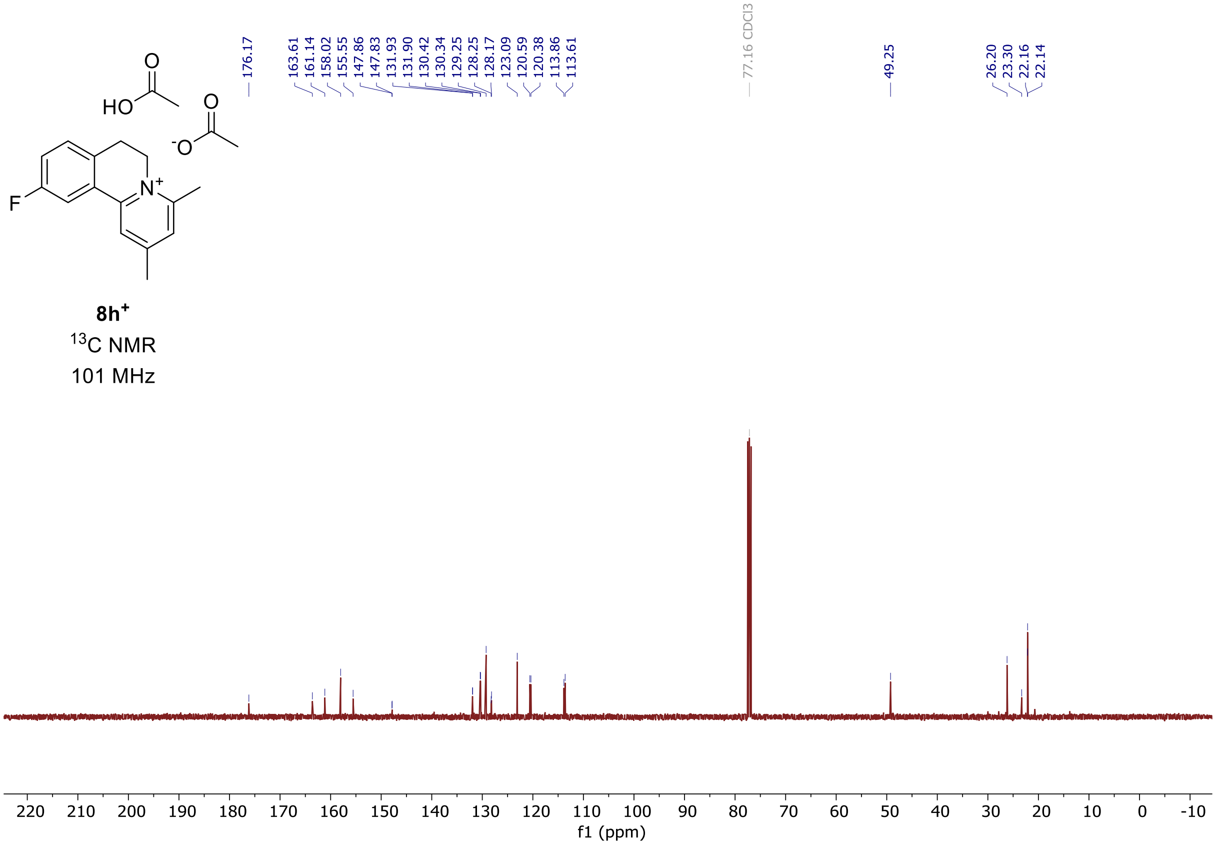

8h⁺

^{13}C NMR

101 MHz

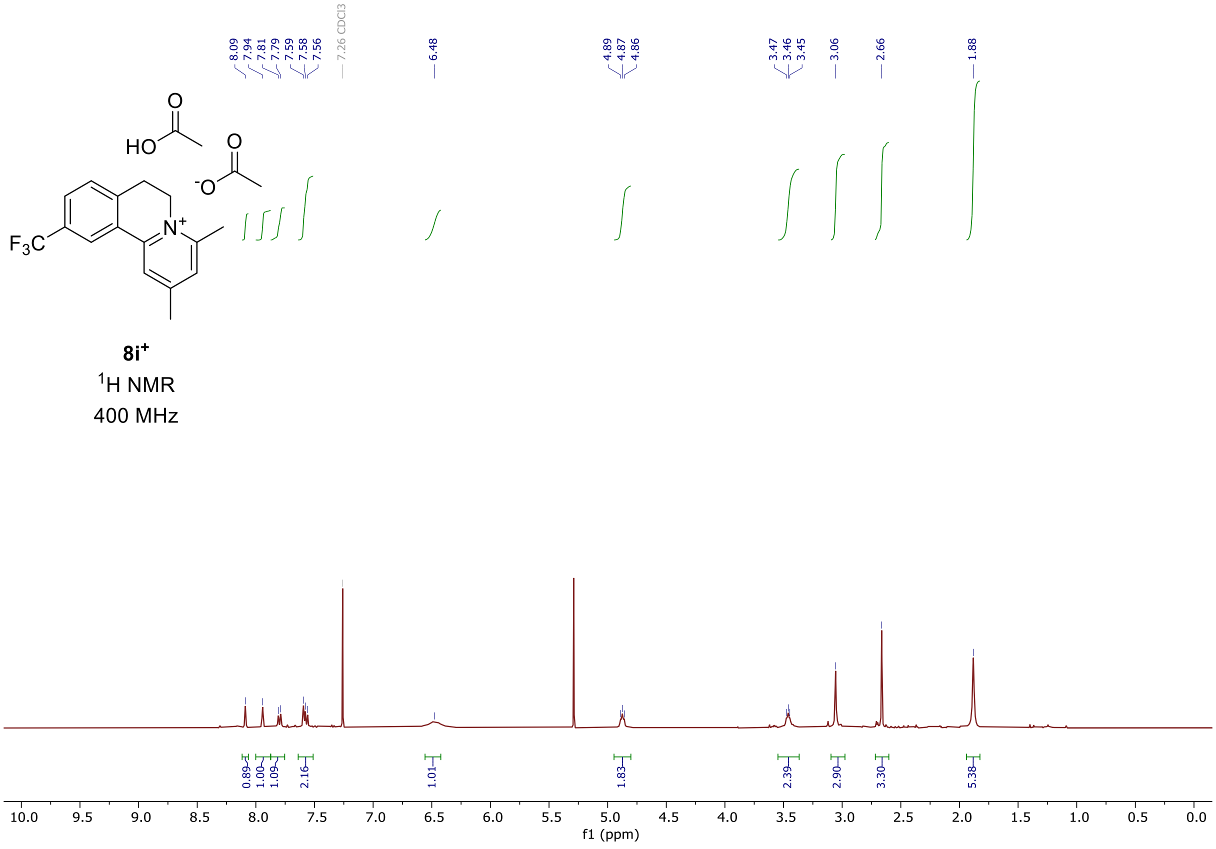

8i⁺

^{1}H NMR

400 MHz

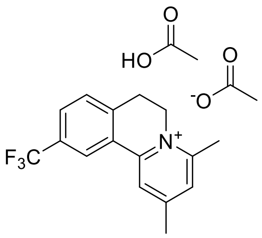

8i⁺

^{19}F NMR

376 MHz

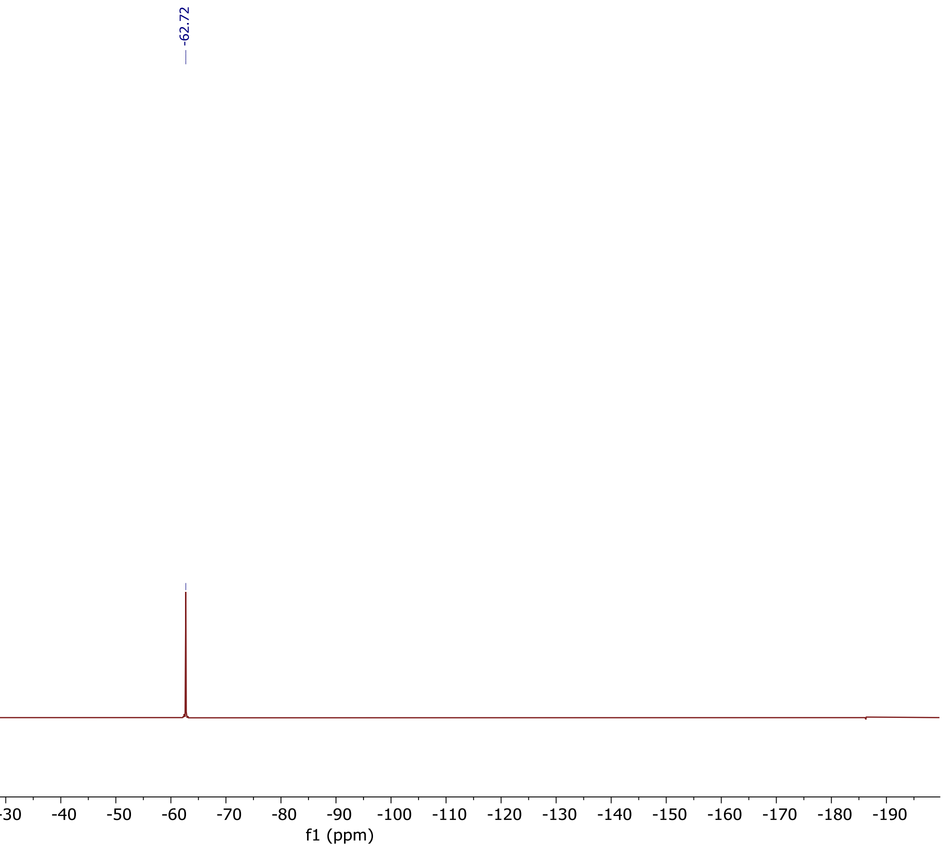

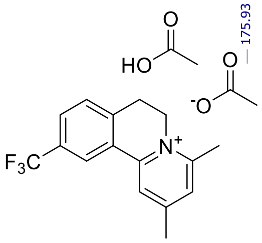

8i⁺

^{13}C NMR

101 MHz

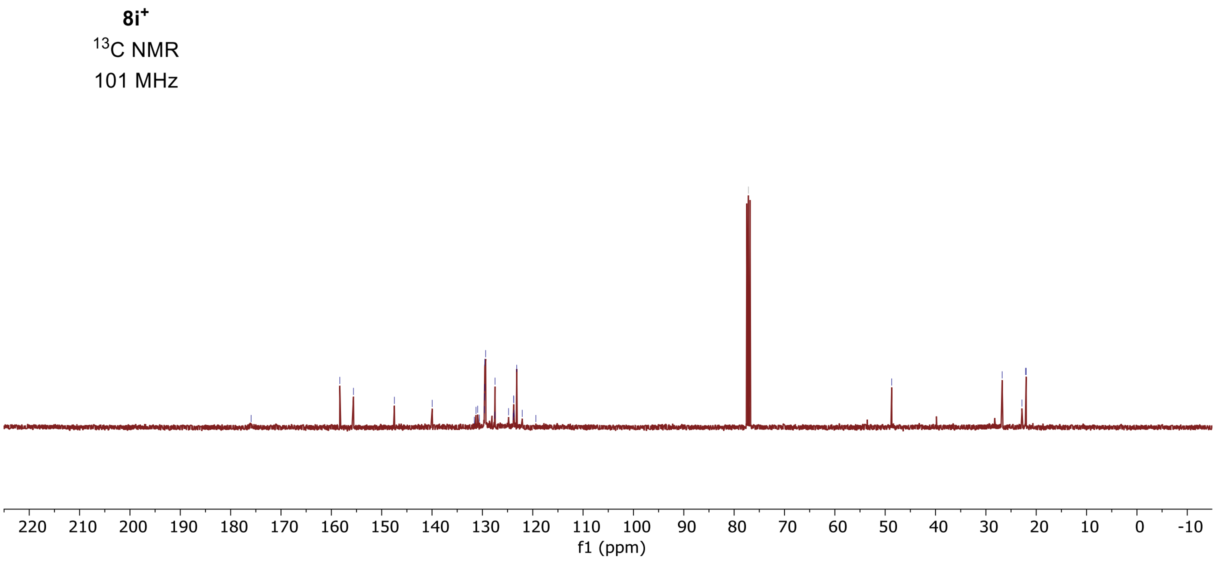

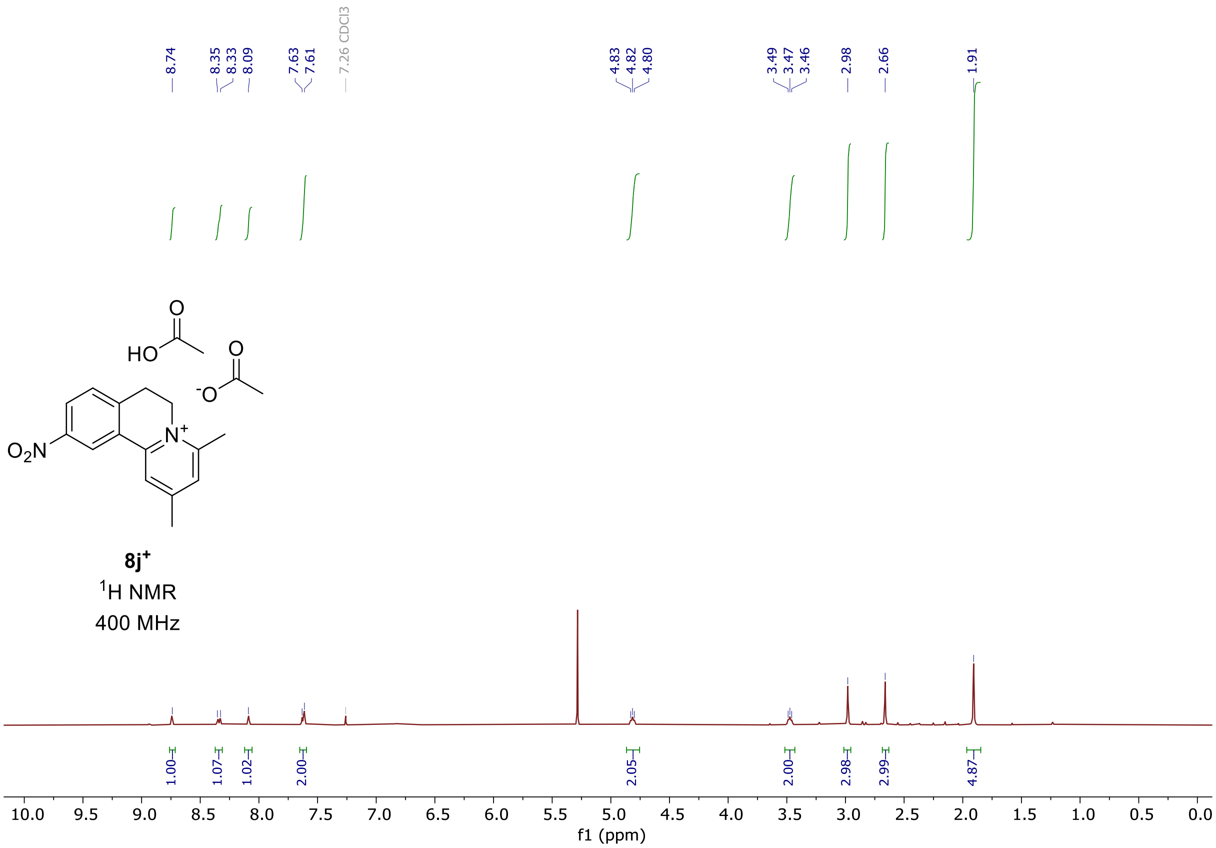
8.74
8.35
8.33
8.09
7.63
7.61
7.26 CDCl3
4.83
4.82
4.80
3.49
3.47
3.46
2.98
2.66
1.91
HO
O
$^{-}$O
O
O_2N
N^+
8j+
1H NMR
400 MHz
1.00
1.07
1.02
2.00
2.05
2.00
2.98
2.99
4.87
f1 (ppm)

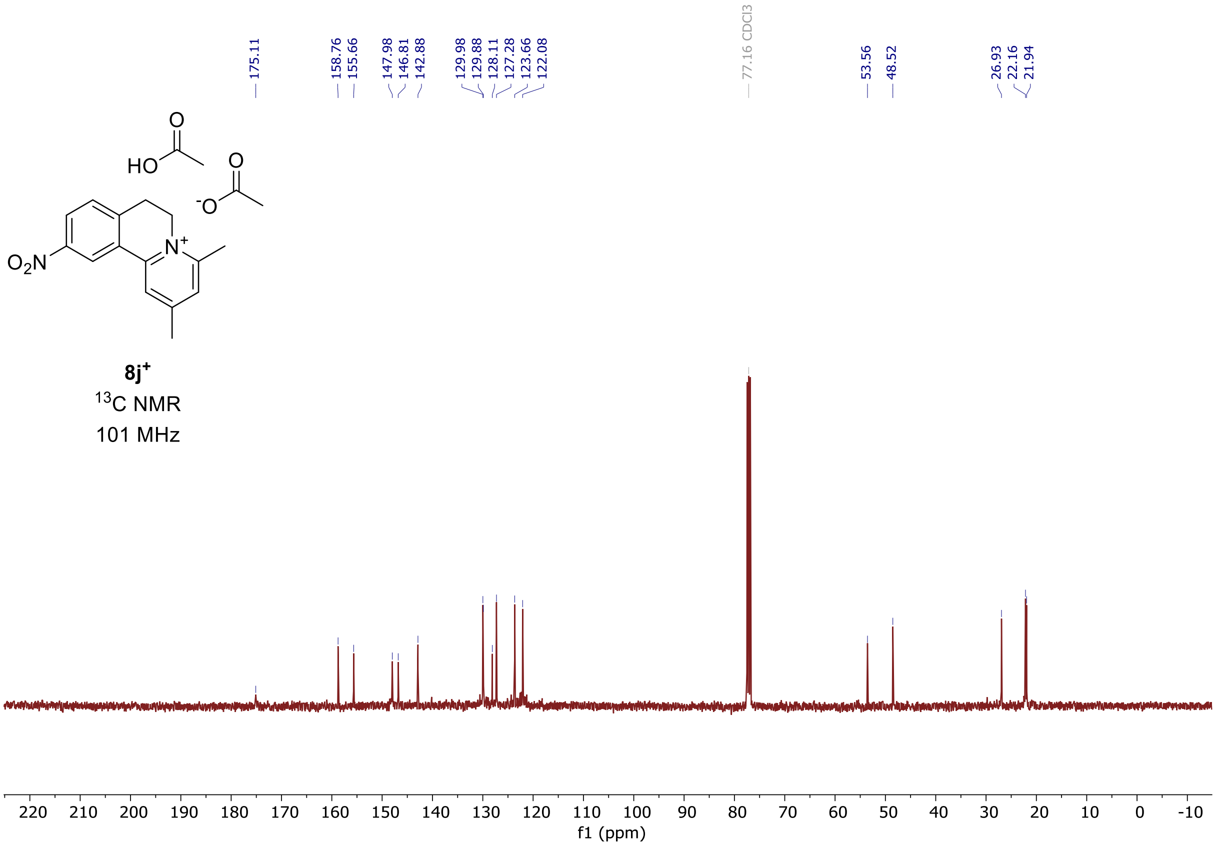
175.11
158.76
155.66
147.98
146.81
142.88
129.98
129.88
128.11
127.28
123.66
122.08
77.16 CDCl3
53.56
48.52
26.93
22.16
21.94
HO
O
$^{-}$O
O
O_2N
N^+
8j+
13C NMR
101 MHz
f1 (ppm)

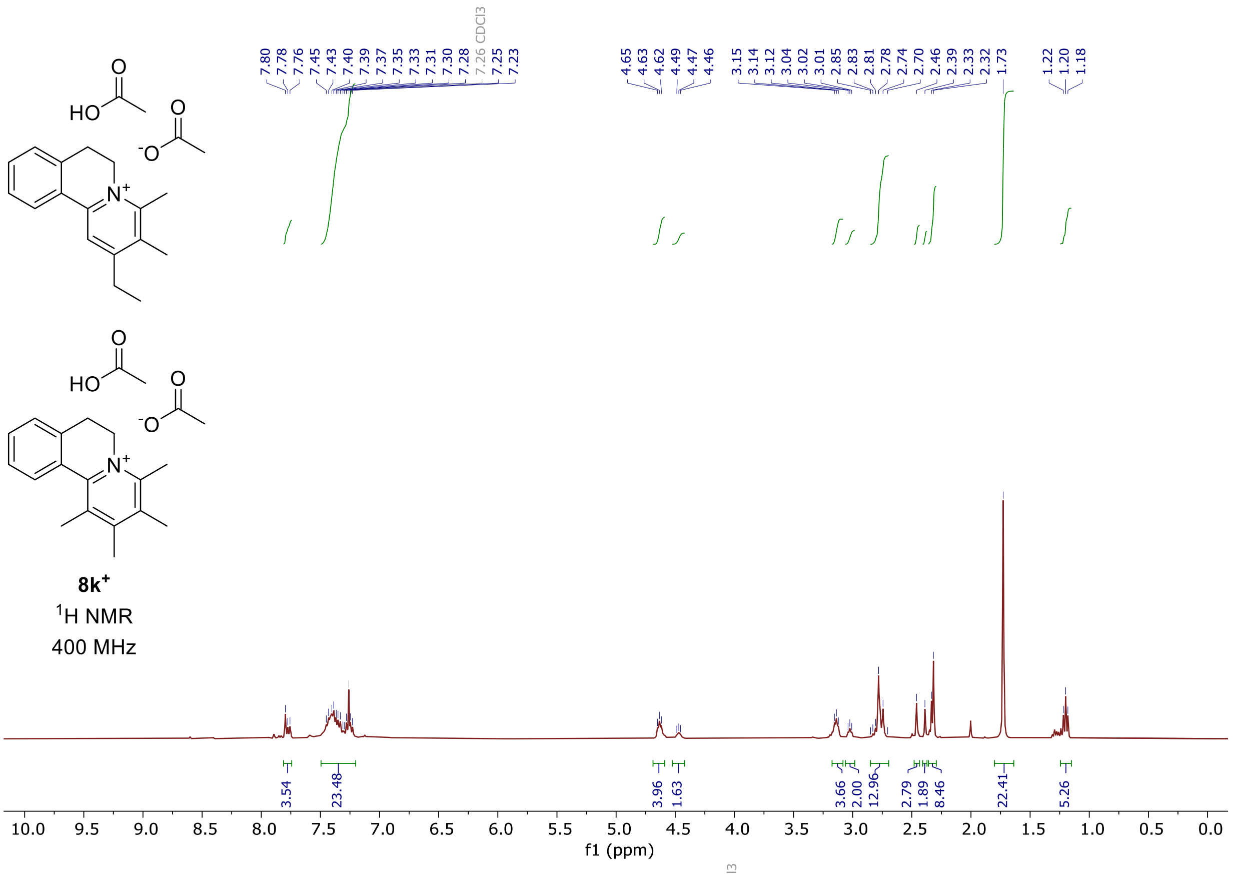
7.80 7.78 7.76 7.45 7.43 7.40 7.39 7.37 7.35 7.33 7.31 7.30 7.28 7.26 CDCl3 7.25 7.23
4.65 4.63 4.62 4.49 4.47 4.46
3.15 3.14 3.12 3.04 3.02 3.01 2.85 2.83 2.81 2.78 2.74 2.70 2.46 2.39 2.33 2.32 1.73
1.22 1.20 1.18
HO
O
-O
N+
8k+
1H NMR
400 MHz
3.54 23.48 3.96 1.63 3.66 2.00 12.96 2.79 1.89 8.46 22.41 5.26
10.0 9.5 9.0 8.5 8.0 7.5 7.0 6.5 6.0 5.5 5.0 4.5 4.0 3.5 3.0 2.5 2.0 1.5 1.0 0.5 0.0
f1 (ppm)

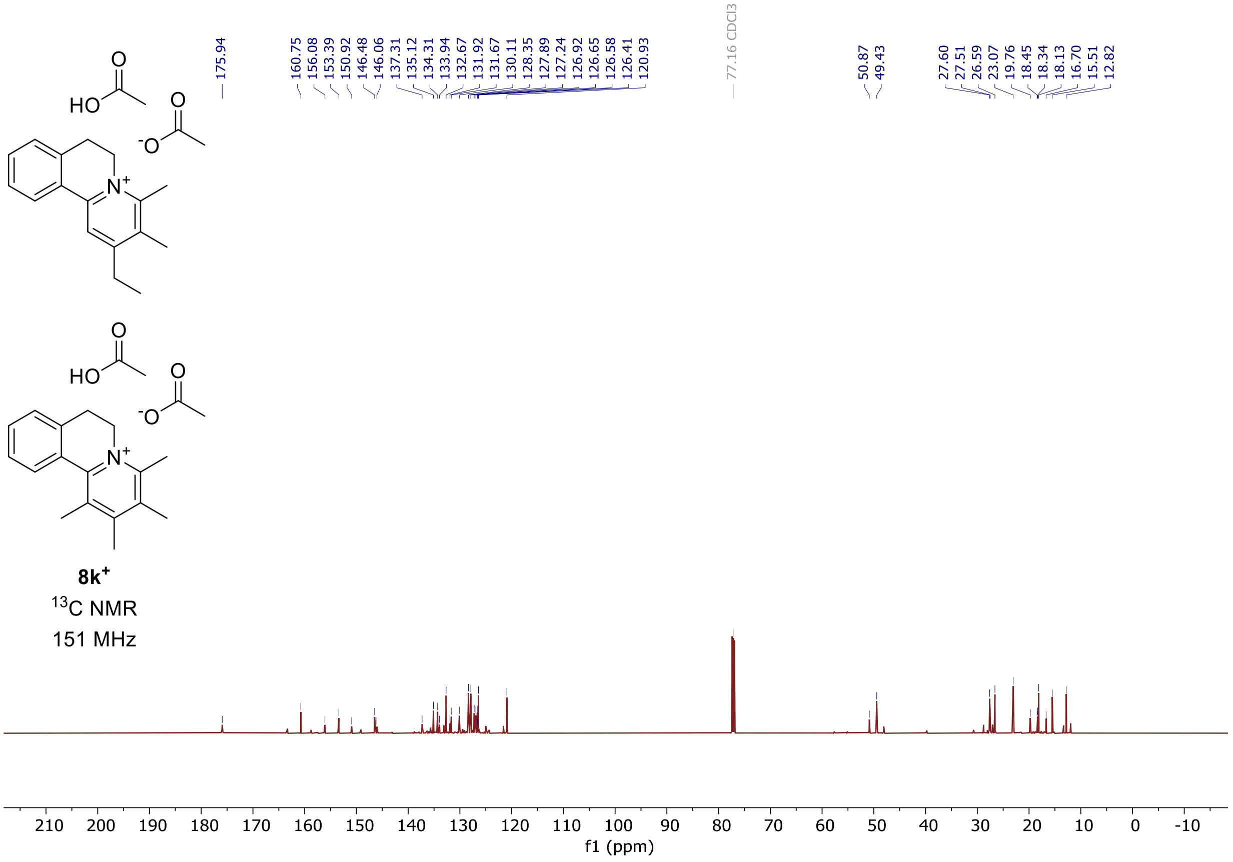
175.94
160.75 156.08 153.39 150.92 146.48 146.06 137.31 135.12 134.31 133.94 132.67 131.92 131.67 130.11 128.35 127.89 127.24 126.92 126.65 126.58 126.41 120.93
77.16 CDCl3
50.87 49.43
27.60 27.51 26.59 23.07 19.76 18.45 18.34 18.13 16.70 15.51 12.82
HO
O
-O
N+
8k+
13C NMR
151 MHz
210 200 190 180 170 160 150 140 130 120 110 100 90 80 70 60 50 40 30 20 10 0 -10
f1 (ppm)

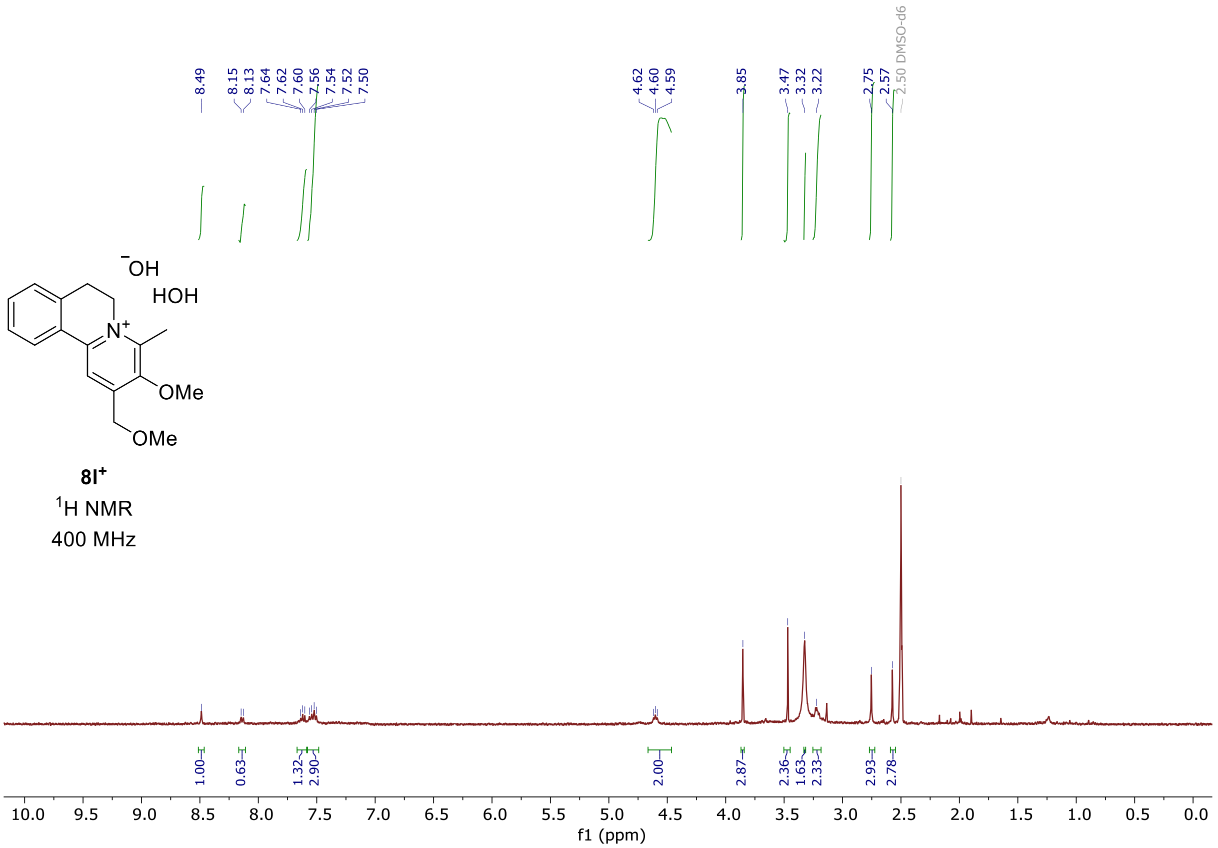

8l+

^{1}H NMR

400 MHz

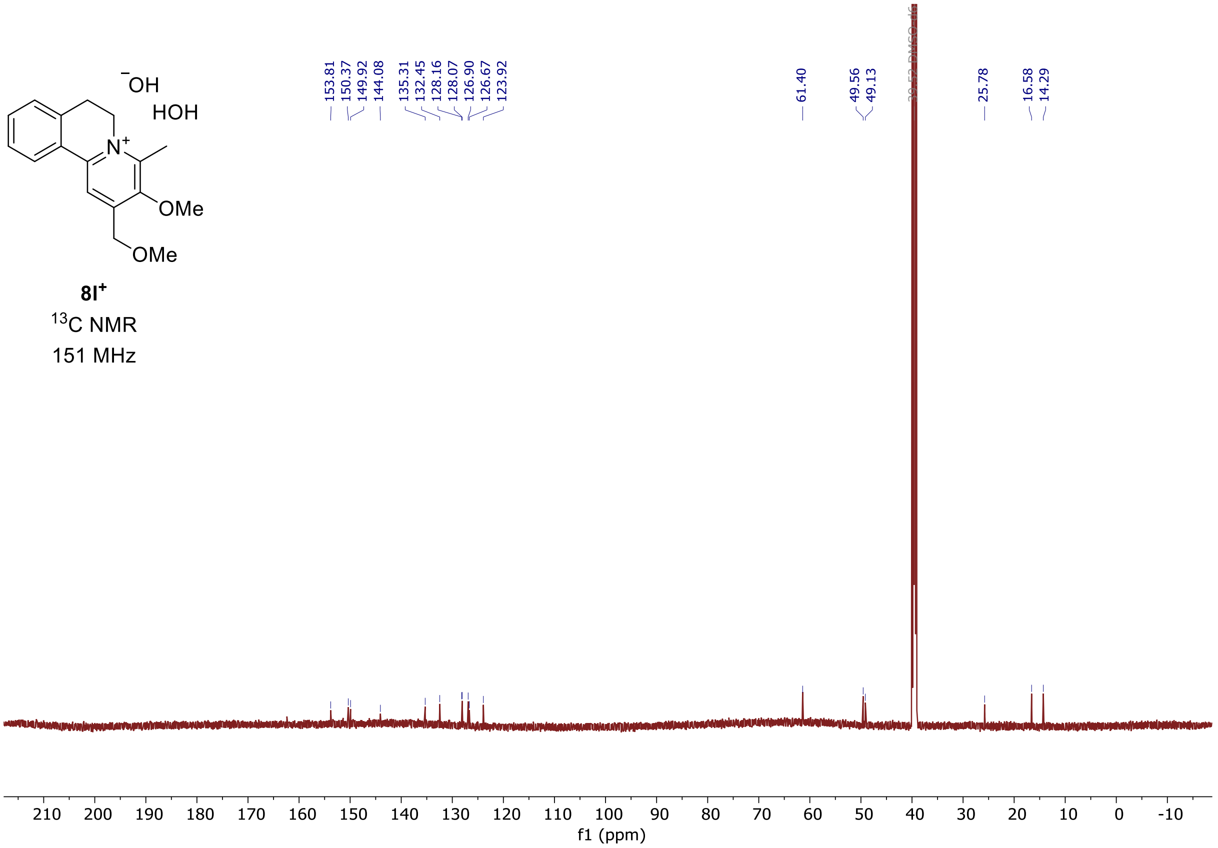

8l+

^{13}C NMR

151 MHz

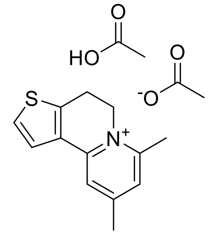

8m+
^{1}H NMR
400 MHz

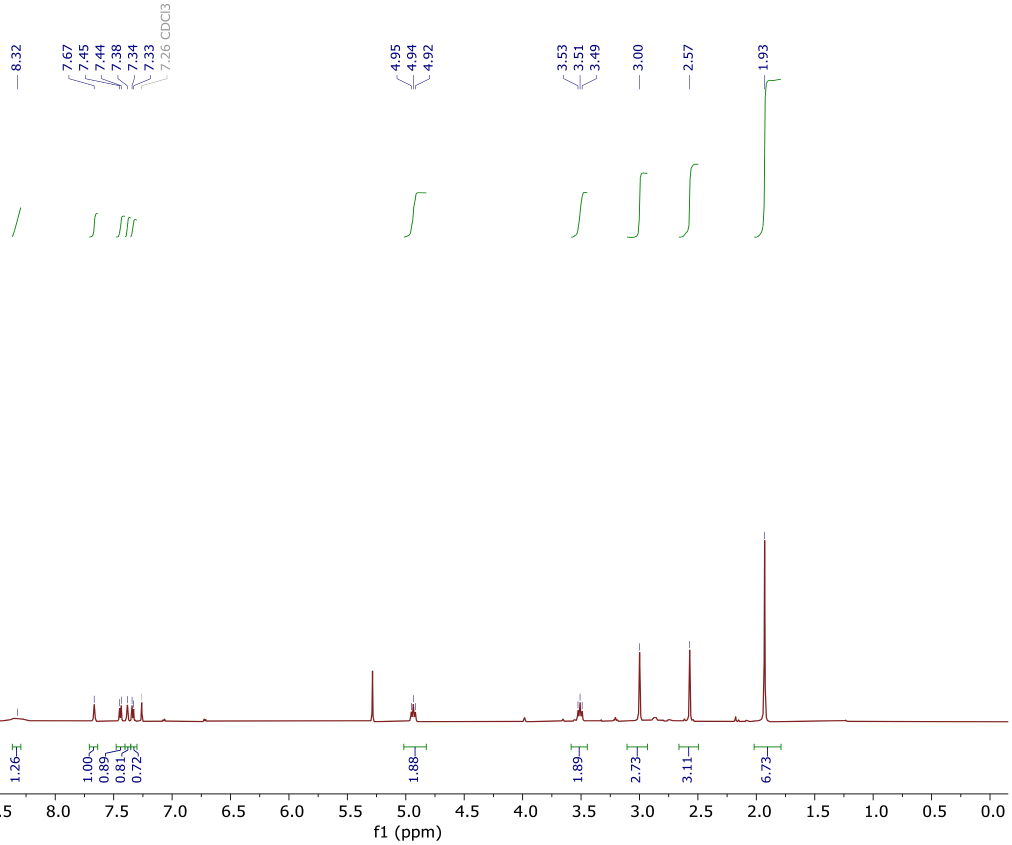

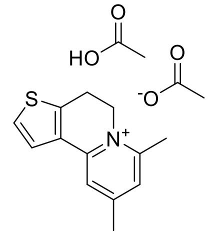

8m+
^{13}C NMR
101 MHz

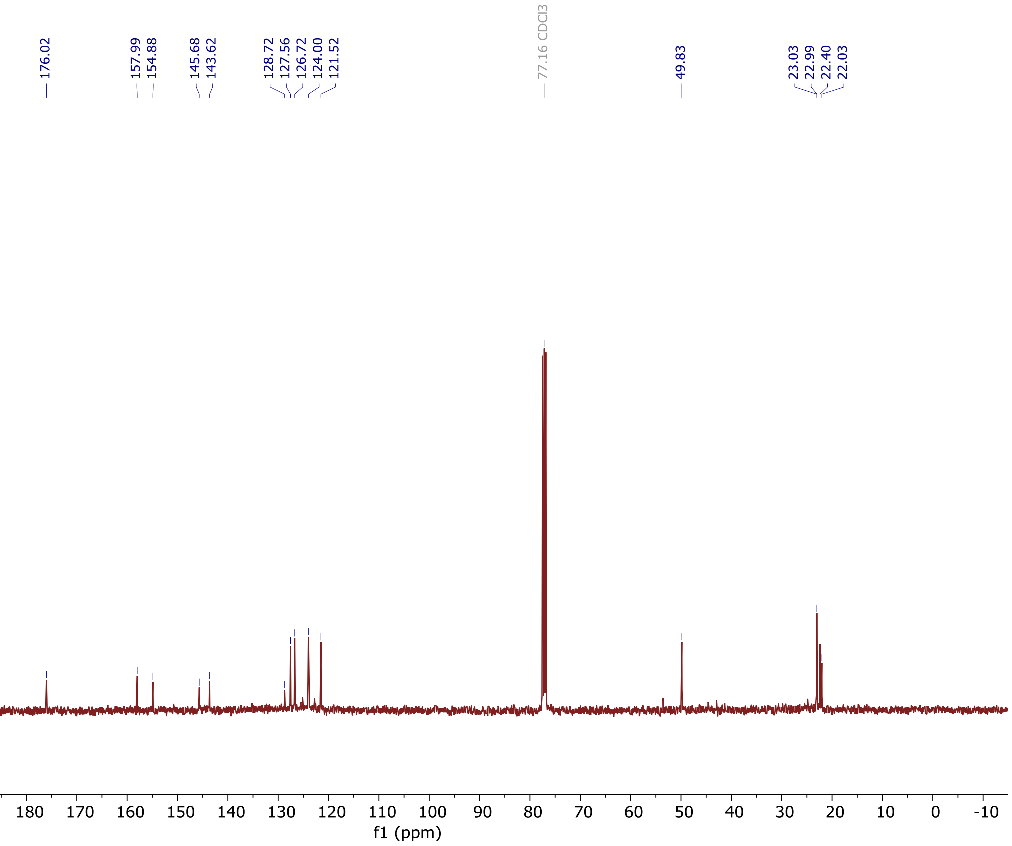

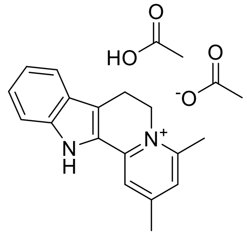

8n$^+$

^{1}H NMR

600 MHz

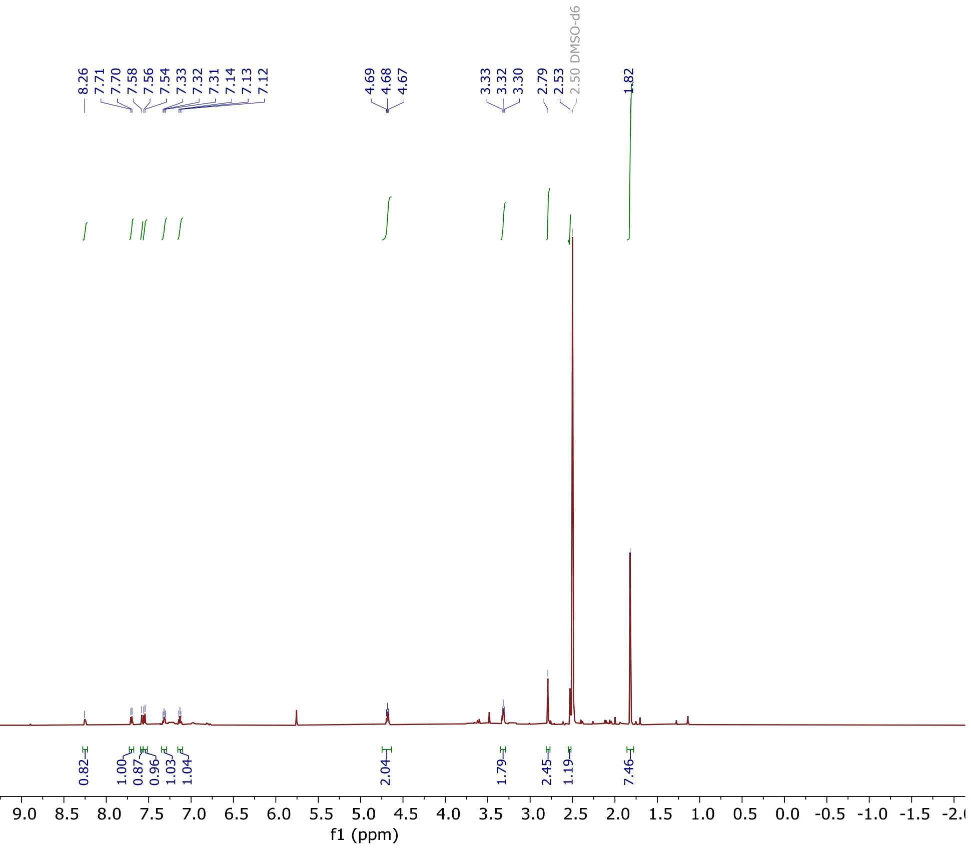

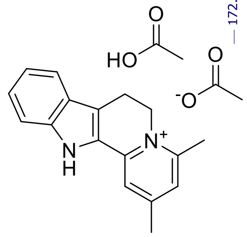

8n$^+$

^{13}C NMR

151 MHz

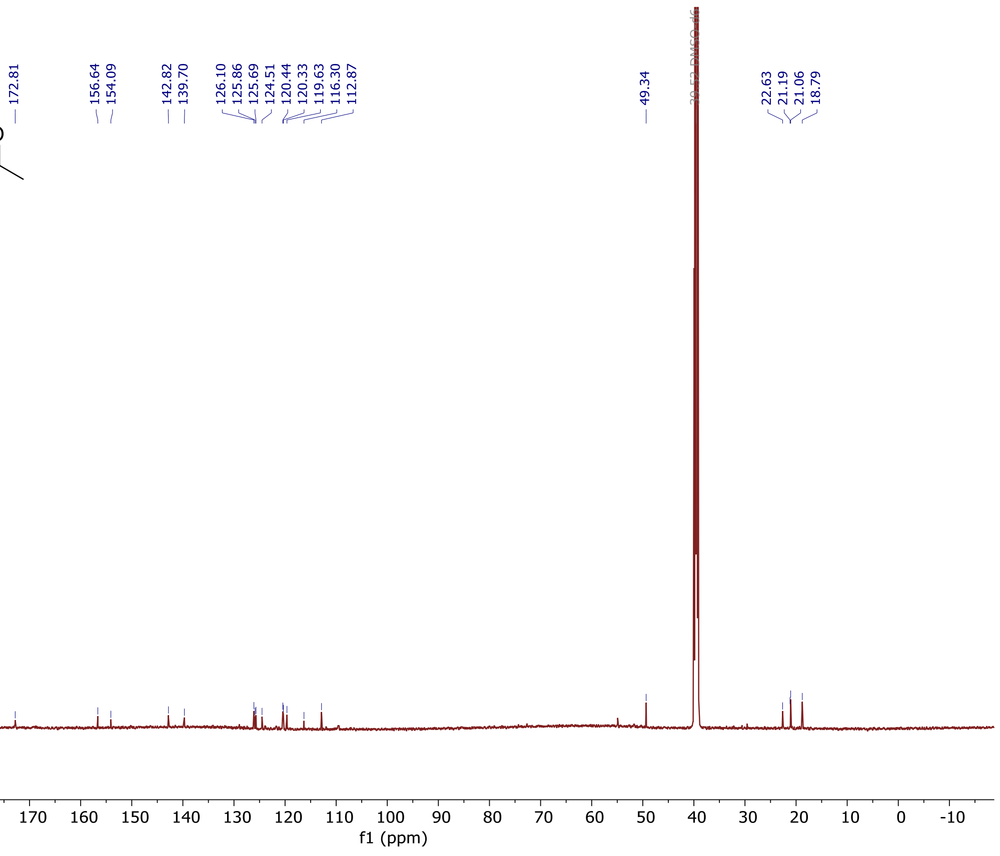

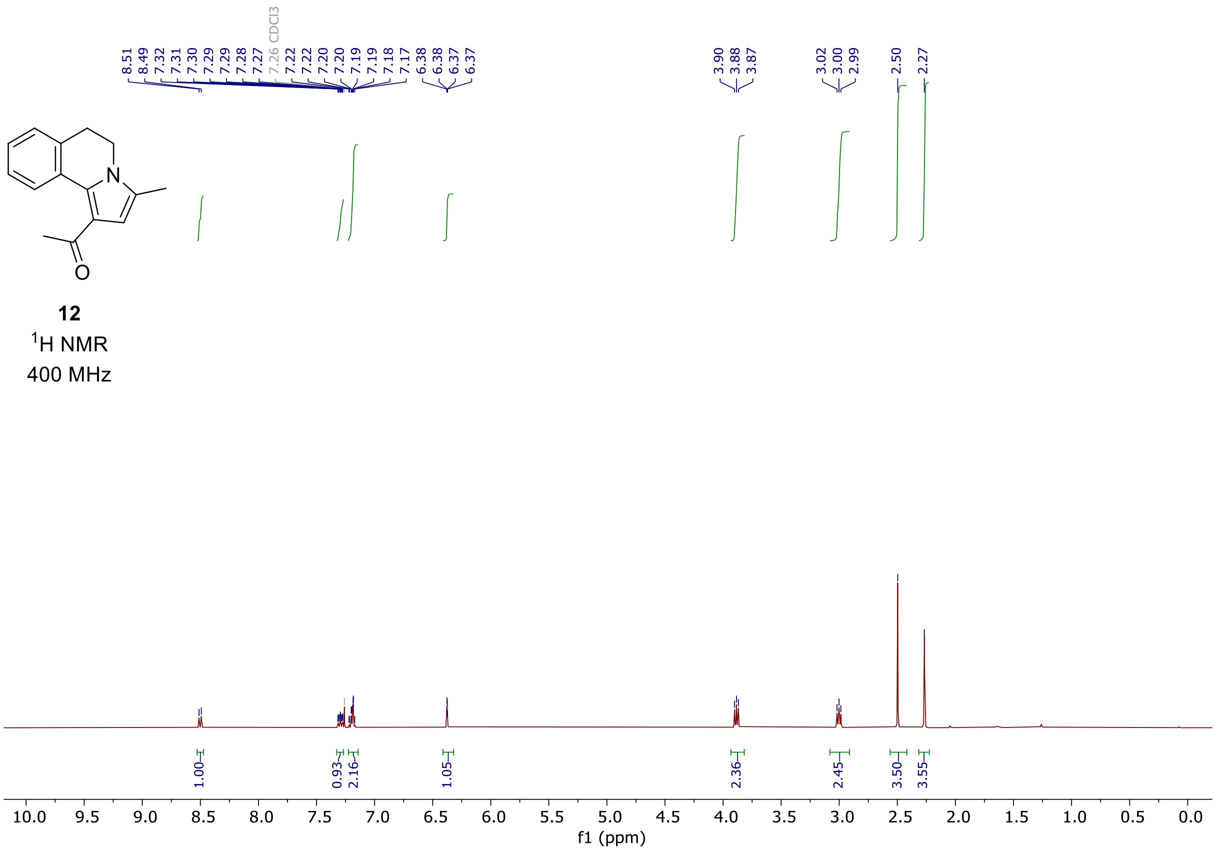

12

^{1}H NMR

400 MHz

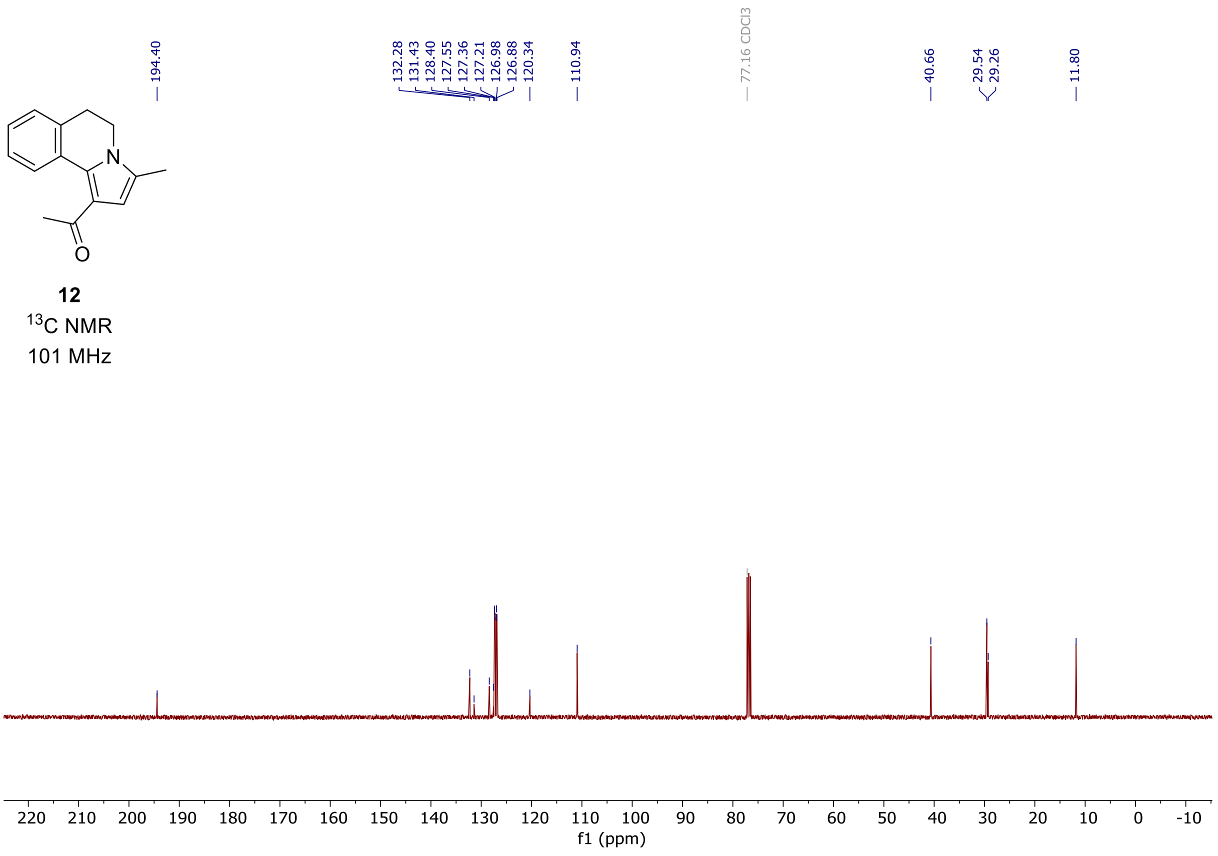

12

^{13}C NMR

101 MHz

XYZ-coordinates

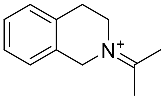

structure **4$^+$**, zero point energy = -521.379245

C	-0.380034000	-0.974618000	0.852070000
C	0.972979000	-0.525200000	0.331653000
C	1.216668000	0.803801000	-0.030200000
C	0.119246000	1.842995000	0.041225000
C	-1.045534000	1.368703000	0.904512000
N	-1.430198000	0.000293000	0.477941000
C	1.991780000	-1.476843000	0.240998000
C	3.255730000	-1.111344000	-0.204411000
C	3.504682000	0.211352000	-0.569420000
C	2.490672000	1.157167000	-0.483610000
H	1.796637000	-2.506409000	0.522177000
H	4.041315000	-1.853636000	-0.269749000
H	4.487264000	0.503312000	-0.918728000
H	2.686922000	2.185267000	-0.767455000
H	0.502296000	2.775535000	0.462537000
H	-0.245117000	2.079073000	-0.965087000
H	-1.900686000	2.033676000	0.861730000
H	-0.741309000	1.279428000	1.949142000
C	-2.489111000	-0.303604000	-0.204172000
H	-0.628554000	-1.953667000	0.456958000
H	-0.386196000	-1.039914000	1.945044000
C	-2.786250000	-1.724399000	-0.579380000
C	-3.474933000	0.717470000	-0.662739000
H	-3.146137000	1.748050000	-0.581616000
H	-3.726309000	0.507289000	-1.705561000
H	-4.402274000	0.590036000	-0.092267000
H	-3.792609000	-1.799609000	-0.986435000
H	-2.085946000	-2.064544000	-1.349589000
H	-2.703274000	-2.398822000	0.274167000

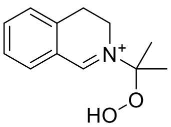

structure **5H$^+$**, zero point energy = -671.785718

O	-2.416615000	-0.675930000	1.818012000
C	0.057129000	0.887086000	0.005622000
N	-0.876391000	0.040596000	-0.307967000
C	-2.355319000	0.364108000	-0.260413000
O	-2.959685000	-0.687707000	0.465082000

C	1.450846000	0.557821000	0.006085000
C	1.832463000	-0.801595000	-0.035224000
C	0.730199000	-1.827416000	-0.026113000
C	-0.491745000	-1.319199000	-0.777367000
C	2.415275000	1.577700000	0.099122000
C	3.760340000	1.246297000	0.116290000
C	4.138342000	-0.097749000	0.066461000
C	3.182205000	-1.115301000	0.001606000
C	-2.941908000	0.239032000	-1.665204000
C	-2.628853000	1.726078000	0.359393000
H	2.103558000	2.615149000	0.144372000
H	4.513117000	2.021690000	0.174114000
H	5.190013000	-0.357853000	0.085277000
H	3.497435000	-2.151893000	-0.017445000
H	1.058640000	-2.762763000	-0.481952000
H	0.457545000	-2.053696000	1.011342000
H	-1.347310000	-1.969851000	-0.622601000
H	-0.289608000	-1.239019000	-1.849855000
H	-0.244813000	1.890024000	0.278352000
H	-2.259406000	2.535139000	-0.272588000
H	-3.710832000	1.836070000	0.433390000
H	-2.209677000	1.811403000	1.360841000
H	-4.008901000	0.456647000	-1.626556000
H	-2.460081000	0.959309000	-2.328052000
H	-2.811499000	-0.764326000	-2.068588000
H	-3.226897000	-0.822285000	2.329409000

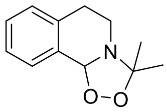

structure **5**, zero point energy = -671.399937

C	-2.843047000	1.178240000	0.343226000
C	-1.512037000	0.831786000	0.122710000
C	-1.200130000	-0.486521000	-0.234024000
C	-2.213841000	-1.432541000	-0.373697000
C	-3.541727000	-1.081156000	-0.143879000
C	-3.854410000	0.226392000	0.220331000
C	-0.380815000	1.825416000	0.167754000
C	0.598356000	1.460391000	-0.953233000
N	1.168243000	0.109946000	-0.827939000
C	0.235867000	-0.911177000	-0.369423000
C	2.351544000	-0.080693000	0.019771000
O	1.791037000	-0.414402000	1.309683000
O	0.732287000	-1.343590000	0.960459000
C	3.209238000	1.152575000	0.252722000
C	3.190436000	-1.246221000	-0.509601000

H	-1.960260000	-2.449280000	-0.654619000
H	-4.326069000	-1.821166000	-0.249867000
H	-4.885840000	0.509259000	0.396684000
H	-3.090837000	2.201676000	0.604821000
H	-0.758888000	2.842271000	0.028464000
H	0.128147000	1.786148000	1.136295000
H	1.415798000	2.173921000	-1.025160000
H	0.064819000	1.502535000	-1.907695000
H	0.307995000	-1.818738000	-0.975851000
H	3.618468000	-0.980795000	-1.478060000
H	3.995815000	-1.472970000	0.191402000
H	2.583717000	-2.142823000	-0.631651000
H	4.055594000	0.875385000	0.882182000
H	3.597392000	1.528096000	-0.695661000
H	2.653259000	1.941843000	0.757212000

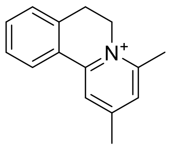

structure **8**$^{+}$, zero point energy = -635.752526

C	4.032636000	0.528317000	-0.008724000
C	3.295973000	1.651743000	-0.385008000
C	1.910537000	1.594188000	-0.414180000
C	1.244915000	0.406182000	-0.070816000
C	1.993004000	-0.731991000	0.279158000
C	3.381087000	-0.658540000	0.316563000
C	-0.220866000	0.347872000	-0.032599000
N	-0.843826000	-0.870944000	-0.137529000
C	0.014494000	-2.078987000	-0.303682000
C	1.234247000	-1.988934000	0.596615000
C	-1.006361000	1.485442000	0.130463000
C	-2.393692000	1.416454000	0.146828000
C	-2.978424000	0.152463000	-0.000765000
C	-2.209462000	-0.984492000	-0.141074000
C	-3.242997000	2.638455000	0.319459000
C	-2.848277000	-2.330883000	-0.304205000
H	5.114481000	0.573702000	0.016455000
H	3.802816000	2.567436000	-0.661725000
H	1.352135000	2.465129000	-0.731498000
H	3.955506000	-1.534846000	0.593786000
H	-0.578866000	-2.953939000	-0.067009000
H	0.308600000	-2.128290000	-1.355964000
H	0.923007000	-2.001207000	1.648071000
H	1.854826000	-2.871982000	0.435632000
H	-0.512810000	2.436278000	0.266864000

H	-4.055281000	0.046842000	-0.000401000
H	-3.899808000	2.528216000	1.186573000
H	-2.639395000	3.535372000	0.453003000
H	-3.885101000	2.779777000	-0.554688000
H	-2.620582000	-2.991739000	0.536902000
H	-3.928988000	-2.211747000	-0.348945000
H	-2.524646000	-2.826123000	-1.222549000

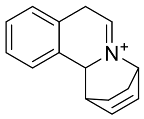

structure **A**, zero point energy = -635.486301

C	3.794545000	-0.478715000	0.284859000
C	3.049644000	-1.639681000	0.077656000
C	1.693251000	-1.557073000	-0.239399000
C	1.076585000	-0.307778000	-0.353736000
C	1.831181000	0.857712000	-0.155874000
C	3.184459000	0.770684000	0.172539000
C	-0.376241000	-0.139157000	-0.764979000
N	-0.965807000	1.046465000	-0.043770000
C	-0.279983000	2.113810000	0.148717000
C	1.138345000	2.201445000	-0.300183000
C	-1.369670000	-1.310639000	-0.539216000
C	-2.690339000	-0.880586000	-1.143031000
C	-3.190409000	0.246004000	-0.626662000
C	-2.356027000	0.823222000	0.491863000
C	-1.574304000	-1.525572000	0.988395000
C	-2.187837000	-0.240667000	1.608191000
H	4.848131000	-0.543938000	0.535340000
H	3.521541000	-2.612700000	0.164633000
H	1.129288000	-2.469381000	-0.399856000
H	3.762224000	1.675697000	0.336178000
H	-0.412669000	0.136803000	-1.827620000
H	-0.763208000	2.939226000	0.668293000
H	1.131411000	2.527828000	-1.355284000
H	1.647944000	2.993187000	0.254791000
H	-0.975513000	-2.207240000	-1.019220000
H	-3.167340000	-1.447604000	-1.935424000
H	-4.119003000	0.718300000	-0.925878000
H	-2.719523000	1.778541000	0.872859000
H	-2.229793000	-2.384746000	1.142140000
H	-0.616240000	-1.758779000	1.459580000
H	-1.558808000	0.160036000	2.408795000
H	-3.176983000	-0.428549000	2.032222000

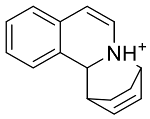

structure **B**, zero point energy = -635.466492

C	-3.793765000	-0.278812000	0.316657000
C	-3.192433000	-1.476320000	-0.070745000
C	-1.843905000	-1.502437000	-0.436495000
C	-1.079961000	-0.334349000	-0.416276000
C	-1.692407000	0.877328000	-0.047570000
C	-3.045012000	0.894539000	0.321822000
C	0.376307000	-0.408358000	-0.847397000
N	1.142683000	0.950935000	-0.755416000
C	0.367115000	2.161966000	-0.434754000
C	-0.919391000	2.109221000	-0.093124000
C	1.222035000	-1.449672000	-0.047134000
C	2.638688000	-1.431464000	-0.571058000
C	3.260431000	-0.253345000	-0.443252000
C	2.404925000	0.809729000	0.182636000
C	1.252587000	-1.033872000	1.447819000
C	1.886280000	0.376508000	1.562545000
H	-4.840021000	-0.258345000	0.601992000
H	-3.770137000	-2.394402000	-0.094831000
H	-1.402435000	-2.444033000	-0.748455000
H	-3.505039000	1.835983000	0.607143000
H	0.415371000	-0.666877000	-1.908871000
H	1.544818000	1.096077000	-1.683956000
H	0.955883000	3.068435000	-0.496292000
H	-1.418718000	3.044704000	0.141065000
H	0.754105000	-2.427119000	-0.169959000
H	3.099773000	-2.315066000	-1.000410000
H	4.282175000	-0.047885000	-0.742691000
H	2.861629000	1.800489000	0.194570000
H	0.238576000	-1.038857000	1.854544000
H	1.831719000	-1.771612000	2.006643000
H	2.735020000	0.384027000	2.250208000
H	1.169541000	1.113970000	1.935166000

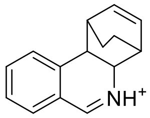

structure **C**, zero point energy = -635.494491

C	3.270699000	-1.464300000	-0.081232000
C	3.856038000	-0.251520000	0.302097000
C	3.077715000	0.894952000	0.325546000
C	1.712685000	0.821037000	-0.026224000
C	1.108824000	-0.404863000	-0.390625000

C	1.915830000	-1.539431000	-0.423286000
C	0.936480000	2.014686000	-0.074046000
N	-0.299086000	2.041510000	-0.479776000
C	-1.120145000	0.870157000	-0.863961000
C	-0.370571000	-0.494009000	-0.715841000
C	-2.414529000	0.862247000	0.009449000
C	-2.009770000	0.586500000	1.438689000
C	-1.341053000	-0.561051000	1.604429000
C	-1.155508000	-1.362788000	0.338311000
C	-2.558900000	-1.651892000	-0.261216000
C	-3.293899000	-0.308110000	-0.512322000
H	3.876601000	-2.364414000	-0.115817000
H	4.906278000	-0.209059000	0.567698000
H	3.510844000	1.851800000	0.602792000
H	1.493640000	-2.495426000	-0.717679000
H	1.381792000	2.967228000	0.204280000
H	-0.777002000	2.937324000	-0.502239000
H	-1.396056000	1.041505000	-1.910189000
H	-0.452418000	-1.014015000	-1.676659000
H	-2.941541000	1.815245000	-0.107583000
H	-2.261139000	1.268699000	2.244358000
H	-0.984422000	-0.920955000	2.564172000
H	-0.613971000	-2.294906000	0.512406000
H	-2.454214000	-2.221911000	-1.190538000
H	-3.118935000	-2.278363000	0.436630000
H	-4.254557000	-0.280090000	0.005991000
H	-3.504671000	-0.162920000	-1.577791000

References

1. Motloch, P.; Jašík, J.; Roithová, J. Gold(I) and Silver(I) π-Complexes with Unsaturated Hydrocarbons. *Organometallics* **2021,** *40,* 1492–1502.

2. Mehara, J.; Roithová, J. Copper(II)-TEMPO Interaction. *Isr. J. Chem.* **2023,** *63,* 1–10.

3. Thomas, G. T.; Donnecke, S.; Chagunda, I. C.; McIndoe, J. S. Pressurized Sample Infusion. *Chemistry-Methods* **2022,** *2,* 1–15.

4. Bütikofer, A.; Chen, P. Cyclopentadienone Iron Complex-Catalyzed Hydrogenation of Ketones: An Operando Spectrometric Study Using Pressurized Sample Infusion-Electrospray Ionization-Mass Spectrometry. *Organometallics* **2022,** *41,* 2349–2364.

5. Roithová, J.; Gray, A.; Andris, E.; Jašík, J.; Gerlich, D. Helium Tagging Infrared Photodissociation Spectroscopy of Reactive Ions. *Acc. Chem. Res.* **2016,** *49,* 223–230.

6. Frisch, M. J.; Trucks, G. W.; Schlegel, H. B.; Scuseria, G. E.; Robb, M. A.; Cheeseman, J. R.; Scalmani, G.; Barone, V.; Petersson, G. A.; Nakatsuji, H.; et al. Gaussian 16. Gaussian, Inc., Revision C.01, 2016.

7. Alagiri, K.; Prabhu, K. R. C–H Functionalization of Tertiary Amines by Cross Dehydrogenative Coupling Reactions: Solvent-Free Synthesis of α-Aminonitriles and β-Nitroamines under Aerobic Condition. *Org. Biomol. Chem.* **2012,** *10,* 835–842.

8. Sueda, T.; Kajishima, D.; Goto, S. Mechanistic Investigations on the Reaction Between Amines or Amides and an Alkylperoxy-λ^3-Iodane. *J. Org. Chem.* **2003,** *68,* 3307–3310.

9. Williams, P. J. H.; Boustead, G. A.; Heard, D. E.; Seakins, P. W.; Rickard, A. R.; Chechik, V. New Approach to the Detection of Short-Lived Radical Intermediates. *J. Am. Chem. Soc.* **2022,** *144,* 15969–15976

10. Nielsen, C. D. T.; Burés, J. Visual Kinetic *Analysis. Chem. Sci*. **2019**, *10*, 348–353.

11. Sheldrick, G. M. A Short History of SHELX. *Acta Crystallogr., Sect. A: Found. Crystallogr.* **2008,** *64,* 112–122.

12. Sheldrick, G. M. Crystal Structure Refinement with SHELXL. *Acta Crystallogr., Sect. C: Struct. Chem.* **2015,** *71,* 3–8.
